# Global, regional, and national burden of multiple myeloma, 1990 to 2021 and predictions to 2035: an analysis of the Global Burden of Disease Study 2021

**DOI:** 10.3389/fmed.2025.1609692

**Published:** 2025-07-31

**Authors:** Xin Miao, ZhaoXian Wang, HanYu Wang, XiaoYu Zeng, JiaHao Wang, Bing Luo, Ye Yang, JiaFu Yang, Lu Zhao

**Affiliations:** ^1^School of Integrated Traditional Chinese and Western Medicine, Southwest Medical University, Luzhou, China; ^2^The Affiliated Traditional Chinese Medicine Hospital, Southwest Medical University, Luzhou, China; ^3^Clinical Medical College, Chengdu University of Traditional Chinese Medicine, Chengdu, China

**Keywords:** multiple myeloma, Global Burden of Disease 2021, incidence, mortality, disability-adjusted life years

## Abstract

**Background and objective:**

Multiple myeloma (MM) is the second most common haematologic malignancy. This study aimed to assess the global burden of multiple myeloma across 204 countries and territories from 1990 to 2021.

**Methods:**

The data for this study were obtained from the Global Burden of Disease (GBD) 2021 dataset, which provides comprehensive information on the global and regional burden of 369 diseases, injuries, and 88 risk factors across 204 countries and territories from 1990 to 2021. We analyzed the incidence, mortality, disability-adjusted life years (DALYs), and disease-related risk factors of MM from 1990 to 2021. Age-standardized incidence rate (ASIR), age-standardized mortality rate (ASMR), and age-standardized DALYs rate (ASDR) were calculated and analyzed. We also analyzed trends over time by gender and age, and assessed the impact of socio-demographic index (SDI) on disease burden. In addition, the global burden of MM from 2021 to 2035 was predicted by a Bayesian age-period-cohort (BAPC) model.

**Results:**

In 2021, there were 148,755 cases of MM globally. From 1990 to 2021, the global ASIR, ASMR, and ASDR increased (EAPC = 0.48, 0.09 and 0.06, respectively). In general, the ASIR, ASMR, and ASDR of MM and SDI levels are positive correlated, as regions with higher SDI levels normally have higher ASIR, ASMR, and ASDR. The incidence and mortality rates were higher in males than in females in all age groups, and increased with age before 90 years. We projected that the ASIR, ASMR, and ASDR of MM would obviously increase over the next dozen years through BAPC model.

**Conclusion:**

The findings in this study offer valuable insights into the global distribution and magnitude of the MM burden, which may be instructive for better making public health policy and reasonably allocating medical source.

## Introduction

Multiple myeloma (MM) is the second most common hematologic malignancy, accounting for approximately 1% of all cancers and approximately 10% of all hematologic malignancies (1–3). It usually evolves from the asymptomatic precancerous stage of clonal plasma cell proliferation which can cause anemia, osteolytic lesions, fractures, hypercalcemia, kidney injury and an increased risk of infection (4–7). According to a cancer statistic in 2020, MM is most common in people aged 65 to 74 years, with a median age of 69 years (8). MM is extremely rare in people under 30 years of age and occurs more frequently in men than women (9). Since 1990, with the development of new targeted drugs and transplantation techniques, the 5-year survival rate of MM has increased worldwide, but the incidence and mortality have been on the rise. From 1990 to 2016, the global incidence of MM increased by 126%, with an estimated 160,000 cases of MM worldwide as of 2018, of which approximately 90,000 were in men and 70,000 in women (10). However, few studies have investigated MM at a worldwide epidemiology and disease burden level in recent years.

While existing study have explored the global impact of MM using the GBD 2019 dataset (11, 12), our study aims to provide the most up-to-date and comprehensive epidemiological analysis of MM by using the latest GBD 2021 dataset and employ advanced statistical methods. In this study, we systematically described the global incidence, mortality and DALYs of MM from 1990 to 2021, focused on the burden of disease by age group, gender and SDI, and projected the temporal trends from 2021 to 2035. It provides valuable reference for formulating more effective public health strategies and distributing medical resources.

## Materials and methods

### Data acquisition and sources

The data for this study came from the GBD 2021 dataset, which provides comprehensive information on the global and regional burden of 369 diseases, injuries, and 88 risk factors in 204 countries and territories from 1990 to 2021. Specifically, we extracted data on MM, including incidence, mortality, and DALYs. In addition, we obtained the SDI data to assess the impact of socioeconomic factors on the burden of disease. All data could be downloaded via the Global Health Data Exchange (GHDx) platform.^1^

For GBD studies, the Institutional Review Board of the University of Washington reviewed and approved a waiver of informed consent.

### Burden description and analysis

In this study, global and regional maps were used to compare the burden of MM, and the study delved into the demographic variables influencing MM, explaining the distribution of the disease’s burden across different age groups and between genders. In addition, we classified countries and regions into five SDI categories to examine the relationship between MM and socioeconomic development. The SDI is known to correlate with disease incidence and mortality rates. Ranging from 0 to 1, a higher SDI indicates greater socioeconomic development (13).

### Risk factor analysis

In this study, we chose high body-mass index as a risk factor for MM to analyze the percentage of DALYs in each region, because high body-mass index has emerged as a consistent and well-established risk factor for MM (14, 15).

### BAPC model for forecasting

To predict the future burden of MM, we employ a BAPC model. The model takes into account the effects of age, period and cohort, providing a comprehensive approach to understanding future trends in disease burden. Based on the assumption that the effects of age, period, and cohort were similar in temporal proximity, Bayesian inference in the BAPC model utilized a second-order stochastic excursion to smooth the prior three aforementioned values and forecast the posterior rates (16). The age effect reflects the differences in health risks among different age groups, the period effect assesses the changes in public health at specific time points, and the cohort effect focuses on the changes in the health status of people born in different eras. By integrating these effects, the BAPC model provides more accurate long-term trend predictions (17). Previous studies have shown that BAPC has higher coverage and accuracy compared to other forecasting methods (18).

### Statistical analysis

Age-standardized rate and estimated annual percentage change (EAPC) were used to study trends in incidence, mortality, and DALYs of MM to eliminate heterogeneity. Based on a previous study, we calculated the EAPC using linear regression (19). When the upper limit of the 95% uncertainty interval (UI) below 0, it indicates a downward trend, while the lower limit exceeds 0, it indicates an upward trend. If 95% UI contains 0, there is no statistically significant change in the trend patterns. All statistical analyses and data visualizations were performed using R (version 4.4.1). For trend analyses, *p*-values <0.05 were considered statistically significant.

## Results

### Global level

From 1990 to 2021, the global incident cases of MM increased from 55,710 (95% UI, 52022.49–59687.84) to 148,755 (95% UI, 131780.43–162049.23). Similarly, the ASIR increased from 1.47 per 100,000 persons (95% UI, 1.37–1.57) in 1990 to 1.74 per 100,000 persons (95% UI, 1.54–1.89) in 2021, the EAPC was 0.48 per 100,000 persons (95% UI, 0.36–0.60). The number of deaths in 2021 remained at 116360 (95% UI: 103078.62–128470.57), with an ASMR of 1.37 per 100,000 persons (95% UI: 1.22–1.52). From 1990 to 2021, the global DALYs number of MM increased from 1,122,517 (95% UI, 1041399.48–1227728.68) to 2,595,595 (95% UI, 2270483.60–2889968.19), and the ASDR increased from 28.34 per 100,000 persons (95% UI, 26.33–30.83) in 1990 to 30.00 per 100,000 persons (95% UI, 26.22–33.37) in 2021 (Tables 1–3).

**Table 1:** Numbers and ASIR per 100,000 persons of incidence of MM in 1990 and 2021, along with EAPC per 100,000 persons from 1990 to 2021, categorized by global, SDI, and GBD regions.

	1990		2021		1990–-2021
Location	Numbers	ASIR per 100,000 persons	Numbers	Numbers	ASIR per 100,000 persons
Global	55710.10 (52022.49–59687.84)	1.47 (1.37–1.57)	148754.63 (131780.43–162049.23)	1.74 (1.54–1.89)	0.48 (0.36–0.60)
High SDI	33358.07 (31614.85–34444.99)	2.98 (2.83–3.08)	68287.53 (61342.09–72525.42)	3.16 (2.87–3.34)	0.15 (−0.03 to 0.33)
High-middle SDI	12561.71 (11834.03–13538.50)	1.27 (1.20–1.37)	34787.52 (30245.11–38625.23)	1.75 (1.52–1.95)	1.01 (0.89–1.14)
Middle SDI	5249.24 (4561.16–6753.37)	0.51 (0.45–0.66)	28497.75 (22906.09–33491.54)	1.05 (0.84–1.23)	2.15 (1.97–2.34)
Low-middle SDI	3221.81 (2332.48–4254.84)	0.54 (0.39–0.71)	13200.85 (11292.66–18482.68)	0.92 (0.79–1.30)	1.72 (1.64–1.81)
Low SDI	1243.33 (695.71–1749.92)	0.56 (0.32–0.79)	3800.95 (2523.31–5130.65)	0.77 (0.51–1.02)	0.95 (0.77–1.13)
Andean Latin America	231.68 (170.38–295.07)	1.15 (0.85–1.46)	1061.02 (824.67–1379.24)	1.80 (1.40–2.33)	1.59 (1.40–1.79)
Australasia	986.94 (920.86–1058.13)	4.16 (3.88–4.46)	2991.10 (2604.81–3385.97)	5.48 (4.77–6.21)	1.03 (0.89–1.18)
Caribbean	615.13 (568.07–677.04)	2.39 (2.20–2.63)	1711.62 (1464.17–1960.81)	3.17 (2.71–3.63)	0.98 (0.85–1.10)
Central Asia	137.45 (118.65–154.11)	0.28 (0.24–0.31)	440.24 (392.23–492.60)	0.50 (0.45–0.56)	2.42 (2.01–2.82)
Central Europe	2181.11 (2046.75–2297.25)	1.44 (1.35–1.51)	4952.49 (4505.71–5371.29)	2.21 (2.01–2.40)	1.36 (1.15–1.57)
Central Latin America	924.10 (894.63–949.68)	1.10 (1.06–1.13)	4143.86 (3699.09–4654.48)	1.63 (1.46–1.83)	1.15 (1.04–1.26)
Central Sub-Saharan Africa	80.97 (53.86–109.39)	0.36 (0.25–0.49)	239.58 (131.71–345.16)	0.44 (0.24–0.63)	0.68 (0.39–0.98)
East Asia	1918.09 (1369.97–3616.06)	0.22 (0.15–0.41)	18188.60 (11881.75–23583.13)	0.83 (0.54–1.07)	3.88 (3.23–4.54)
Eastern Europe	2943.88 (2788.63–3117.74)	1.03 (0.97–1.09)	6169.99 (5703.09–6689.94)	1.76 (1.63–1.90)	1.90 (1.63–2.17)
Eastern Sub-Saharan Africa	644.67 (351.09–923.84)	0.89 (0.50–1.26)	2059.27 (1252.58–2823.42)	1.24 (0.77–1.68)	1.06 (0.93–1.18)
High-income Asia Pacific	3969.43 (3680.48–4198.14)	1.99 (1.84–2.11)	9740.91 (8163.33–10907.09)	1.93 (1.66–2.16)	−0.04 (−0.22 to 0.15)
High-income North America	11849.70 (11190.60–12239.38)	3.34 (3.17–3.45)	20898.21 (19023.84–22011.04)	3.10 (2.83–3.26)	−0.39 (−0.55 to −0.24)
North Africa and Middle East	1370.38 (953.67–1860.84)	0.83 (0.58–1.13)	5840.45 (4386.47–8004.51)	1.30 (0.97–1.77)	1.60 (1.50–1.69)
Oceania	9.06 (5.60–13.31)	0.33 (0.21–0.48)	26.07 (14.90–36.97)	0.36 (0.21–0.50)	0.28 (0.23–0.33)
South Asia	3537.54 (2253.86–4500.74)	0.63 (0.40–0.80)	15905.44 (12551.44–21561.87)	1.09 (0.86–1.47)	1.62 (1.45–1.79)
Southeast Asia	755.65 (601.15–1226.10)	0.30 (0.24–0.48)	3507.81 (2721.45–5680.86)	0.53 (0.41–0.86)	1.80 (1.75–1.85)
Southern Latin America	1027.95 (954.38–1113.81)	2.21 (2.06–2.40)	2033.22 (1875.64–2190.13)	2.33 (2.15–2.50)	0.25 (0.07–0.42)
Southern Sub-Saharan Africa	403.34 (275.98–521.29)	1.49 (1.01–1.94)	1351.39 (890.93–1639.46)	2.30 (1.51–2.77)	1.49 (1.35–1.63)
Tropical Latin America	1200.33 (1149.39–1245.41)	1.30 (1.23–1.35)	5412.46 (5051.03–5693.06)	2.10 (1.95–2.21)	1.49 (1.31–1.67)
Western Europe	20706.26 (19518.47–21531.44)	3.54 (3.35–3.68)	41185.15 (36907.09–44033.08)	4.30 (3.91–4.57)	0.64 (0.42–0.87)
Western Sub-Saharan Africa	216.46 (112.51–300.98)	0.26 (0.13–0.36)	895.76 (366.04–1315.88)	0.48 (0.20–0.69)	2.15 (2.00–2.29)

**Table 2:** Numbers and ASMR per 100,000 persons of mortality of MM in 1990 and 2021, along with EAPC per 100,000 persons from 1990 to 2021, categorized by global, SDI, and GBD regions.

	1990		2021		1990–2021
Location	Numbers	ASMR per 100,000 persons	Numbers	ASMR per 100,000 persons	EAPC per 100,000 persons (95% UI)
Global	47568.96 (44137.51–51416.50)	1.29 (1.20–1.39)	116359.63 (103078.62–128470.57)	1.37 (1.22–1.52)	0.09 (−0.01 to 0.19)
High SDI	28142.69 (26550.76–28971.06)	2.50 (2.36–2.57)	51434.77 (45704.74–54830.47)	2.28 (2.05–2.41)	−0.43 (−0.54 to −0.31)
High-middle SDI	10133.47 (9534.96–10990.15)	1.05 (0.99–1.14)	25451.41 (22131.83–28174.45)	1.28 (1.12–1.43)	0.59 (0.47–0.71)
Middle SDI	4837.77 (4187.44–6267.88)	0.49 (0.43–0.63)	23404.77 (18799.77–27500.91)	0.88 (0.71–1.04)	1.72 (1.53–1.92)
Low-middle SDI	3155.97 (2278.87–4161.56)	0.55 (0.39–0.71)	12282.85 (10526.79–17215.46)	0.89 (0.76–1.24)	1.53 (1.46–1.61)
Low SDI	1235.17 (688.66–1741.47)	0.58 (0.33–0.81)	3648.51 (2427.37–4903.81)	0.77 (0.52–1.02)	0.87 (0.69–1.04)
Andean Latin America	223.47 (164.79–281.77)	1.13 (0.83–1.42)	883.05 (692.84–1147.24)	1.51 (1.19–1.96)	1.08 (0.90–1.26)
Australasia	670.40 (628.72–708.79)	2.82 (2.65–2.99)	1656.89 (1438.03–1854.82)	2.89 (2.52–3.23)	0.12 (0.01–0.24)
Caribbean	465.30 (433.06–517.14)	1.83 (1.70–2.03)	1109.55 (957.22–1256.43)	2.05 (1.77–2.33)	0.45 (0.36–0.55)
Central Asia	126.79 (109.98–141.87)	0.26 (0.23–0.29)	386.93 (344.40–434.15)	0.45 (0.41–0.51)	2.28 (1.90–2.66)
Central Europe	2028.90 (1908.09–2140.99)	1.35 (1.27–1.42)	4421.73 (4024.89–4787.71)	1.92 (1.75–2.08)	1.08 (0.90–1.26)
Central Latin America	843.60 (817.75–865.88)	1.03 (0.99–1.06)	3331.20 (2986.40–3732.07)	1.33 (1.19–1.49)	0.73 (0.64–0.83)
Central Sub-Saharan Africa	79.87 (53.42–107.82)	0.38 (0.26–0.50)	227.32 (123.87–329.03)	0.44 (0.23–0.63)	0.59 (0.31–0.87)
East Asia	1763.50 (1244.25–3362.14)	0.21 (0.14–0.40)	13624.87 (9018.09–17739.98)	0.63 (0.41–0.81)	2.99 (2.28–3.70)
Eastern Europe	2459.23 (2333.58–2585.32)	0.86 (0.82–0.91)	4652.41 (4295.98–5042.98)	1.31 (1.21–1.42)	1.51 (1.29–1.73)
Eastern Sub-Saharan Africa	642.69 (352.08–919.07)	0.92 (0.52–1.31)	1974.25 (1210.37–2698.97)	1.25 (0.78–1.69)	0.96 (0.85–1.07)
High-income Asia Pacific	3029.79 (2836.32–3195.03)	1.54 (1.43–1.63)	6975.35 (5830.41–7775.25)	1.28 (1.09–1.41)	−0.77 (−0.90 to −0.64)
High-income North America	11712.79 (10989.62–12106.64)	3.25 (3.06–3.36)	19376.80 (17536.36–20470.33)	2.81 (2.56–2.96)	−0.67 (−0.79 to −0.56)
North Africa and Middle East	1289.90 (897.81–1752.96)	0.81 (0.57–1.11)	4708.30 (3544.44–6487.81)	1.10 (0.83–1.51)	1.10 (1.02–1.18)
Oceania	8.38 (5.17–12.43)	0.32 (0.21–0.48)	23.81 (13.48–34.44)	0.34 (0.20–0.49)	0.21 (0.16–0.26)
South Asia	3476.21 (2220.52–4424.90)	0.64 (0.41–0.82)	14790.96 (11658.64–20033.08)	1.04 (0.83–1.41)	1.41 (1.25–1.56)
Southeast Asia	702.87 (557.91–1149.07)	0.29 (0.23–0.47)	2985.82 (2308.44–4846.57)	0.46 (0.36–0.75)	1.50 (1.44–1.55)
Southern Latin America	957.53 (890.88–1033.48)	2.08 (1.93–2.24)	1675.33 (1544.38–1791.27)	1.89 (1.75–2.02)	−0.22 (−0.39 to −0.06)
Southern Sub-Saharan Africa	385.94 (262.77–502.67)	1.47 (0.99–1.93)	1235.38 (817.14–1492.84)	2.18 (1.44–2.62)	1.35 (1.18–1.51)
Tropical Latin America	1096.87 (1047.24–1136.07)	1.22 (1.16–1.27)	4585.74 (4254.13–4822.71)	1.80 (1.66–1.89)	1.24 (1.08–1.40)
Western Europe	15385.47 (14492.51–15919.08)	2.58 (2.44–2.66)	26874.68 (23758.84–28878.23)	2.59 (2.33–2.76)	0.00 (−0.13 to 0.12)
Western Sub-Saharan Africa	219.45 (113.46–305.77)	0.27 (0.14–0.38)	859.26 (363.20–1242.56)	0.48 (0.20–0.69)	2.01 (1.89–2.14)

**Table 3:** Numbers and ASDR per 100,000 persons of DALYs of MM in 1990 and 2021, along with EAPC per 100,000 persons from 1990 to 2021, categorized by global, SDI, and GBD regions.

	1990		2021		1990–2021
Location	Numbers	ASDR per 100,000 persons	Numbers	ASDR per 100,000 persons	EAPC per 100,000 persons (95% UI)
Global	1122517.31 (1041399.48–1227728.68)	28.34 (26.33–30.83)	2595594.99 (2270483.60–2889968.19)	30.00 (26.22–33.37)	0.06 (−0.04 to 0.15)
High SDI	609781.35 (585948.41–625027.66)	55.37 (53.27–56.75)	976932.53 (896756.87–1033833.11)	47.33 (44.00–49.82)	−0.64 (−0.76 to −0.51)
High-middle SDI	254005.86 (239764.50–277110.15)	25.02 (23.59–27.25)	583311.88 (504141.16–652354.98)	29.65 (25.53–33.21)	0.47 (0.37–0.57)
Middle SDI	135857.94 (117165.68–175791.40)	12.23 (10.59–15.76)	609118.62 (487413.38–714664.77)	21.85 (17.50–25.56)	1.68 (1.48–1.88)
Low-middle SDI	87031.83 (62726.59–115403.00)	13.45 (9.70–17.75)	323359.00 (274506.55–449769.08)	21.51 (18.34–29.96)	1.49 (1.41–1.56)
Low SDI	34292.99 (18917.44–48485.48)	14.26 (7.97–20.10)	99828.04 (66163.61–136137.06)	18.43 (12.25–24.91)	0.75 (0.59–0.92)
Andean Latin America	5896.05 (4316.82–7408.15)	27.75 (20.35–34.85)	22215.57 (17273.28–29040.78)	36.98 (28.79–48.20)	1.04 (0.85–1.22)
Australasia	14960.76 (14101.45–15747.58)	63.56 (59.99–66.95)	32239.51 (28598.20–35867.69)	60.65 (54.09–67.37)	−0.08 (−0.18 to 0.02)
Caribbean	11307.32 (10445.89–12749.79)	43.02 (39.75–48.42)	26898.78 (23074.04–30706.47)	49.86 (42.79–56.92)	0.52 (0.42–0.62)
Central Asia	4070.64 (3526.78–4563.27)	7.81 (6.78–8.78)	11936.87 (10635.39–13424.19)	13.06 (11.63–14.64)	2.06 (1.72–2.41)
Central Europe	50815.08 (47917.29–53407.43)	33.22 (31.32–34.95)	95122.76 (86993.37–103200.32)	43.87 (40.13–47.69)	0.83 (0.65–1.01)
Central Latin America	23525.52 (22810.00–24147.51)	26.40 (25.60–27.11)	88782.20 (79138.44–99663.71)	34.39 (30.68–38.61)	0.73 (0.64–0.83)
Central Sub-Saharan Africa	2296.35 (1506.41–3140.62)	9.38 (6.27–12.67)	6648.03 (3617.55–9691.08)	10.89 (5.93–15.83)	0.58 (0.31–0.85)
East Asia	51846.36 (37024.82–98327.43)	5.36 (3.80–10.25)	354332.88 (228713.83–464089.74)	16.31 (10.44–21.37)	3.03 (2.35–3.70)
Eastern Europe	69119.71 (65361.75–72840.36)	24.23 (22.90–25.55)	118762.58 (109042.83–128799.37)	34.51 (31.74–37.42)	1.19 (1.00–1.38)
Eastern Sub-Saharan Africa	17754.32 (9542.87–25618.02)	22.37 (12.26–32.03)	55541.39 (33194.22–77262.03)	30.11 (18.31–41.16)	0.94 (0.83–1.05)
High-income Asia Pacific	69220.36 (65580.42–72653.70)	33.90 (32.00–35.60)	118265.86 (101403.55–130303.06)	25.57 (22.47–27.98)	−1.05 (−1.20 to −0.90)
High-income North America	252724.92 (242624.07–259512.53)	73.46 (70.75–75.37)	374036.80 (348987.11–390777.23)	57.28 (53.80–59.76)	−1.04 (−1.16 to −0.92)
North Africa and Middle East	35663.81 (24579.60–48760.99)	19.80 (13.69–26.95)	124880.86 (93507.46–171852.77)	26.02 (19.48–35.88)	0.96 (0.89–1.04)
Oceania	247.93 (149.73–369.32)	7.76 (4.81–11.52)	697.43 (384.42–1013.44)	8.43 (4.77–12.16)	0.29 (0.23–0.34)
South Asia	97101.04 (61059.67–123438.46)	15.82 (10.10–20.14)	383531.77 (302576.41–511789.75)	24.96 (19.65–33.42)	1.32 (1.17–1.47)
Southeast Asia	19905.61 (15810.49–32175.95)	7.24 (5.76–11.77)	80580.27 (62894.12–128420.15)	11.53 (8.96–18.56)	1.42 (1.36–1.48)
Southern Latin America	23126.63 (21634.76–24929.33)	49.35 (46.14–53.18)	37800.09 (35378.24–40287.46)	43.97 (41.24–46.90)	−0.31 (−0.47 to −0.14)
Southern Sub-Saharan Africa	10830.11 (7509.70–13641.38)	37.35 (25.56–47.90)	34716.78 (23139.65–42583.37)	55.55 (36.80–67.46)	1.38 (1.22–1.54)
Tropical Latin America	30472.11 (29402.68–31534.48)	31.23 (30.02–32.36)	113479.18 (107328.86–118279.68)	43.43 (41.00–45.28)	0.98 (0.81–1.14)
Western Europe	326015.44 (311080.87–336759.66)	57.28 (54.88–59.05)	492170.35 (447363.56–523560.57)	53.56 (49.55–56.61)	−0.25 (−0.39 to −0.11)
Western Sub-Saharan Africa	5617.23 (2902.39–7816.80)	6.23 (3.22–8.66)	22955.02 (9456.92–33911.46)	11.10 (4.68–16.12)	2.04 (1.91–2.18)

### SDI regional level

**Figure 1:**
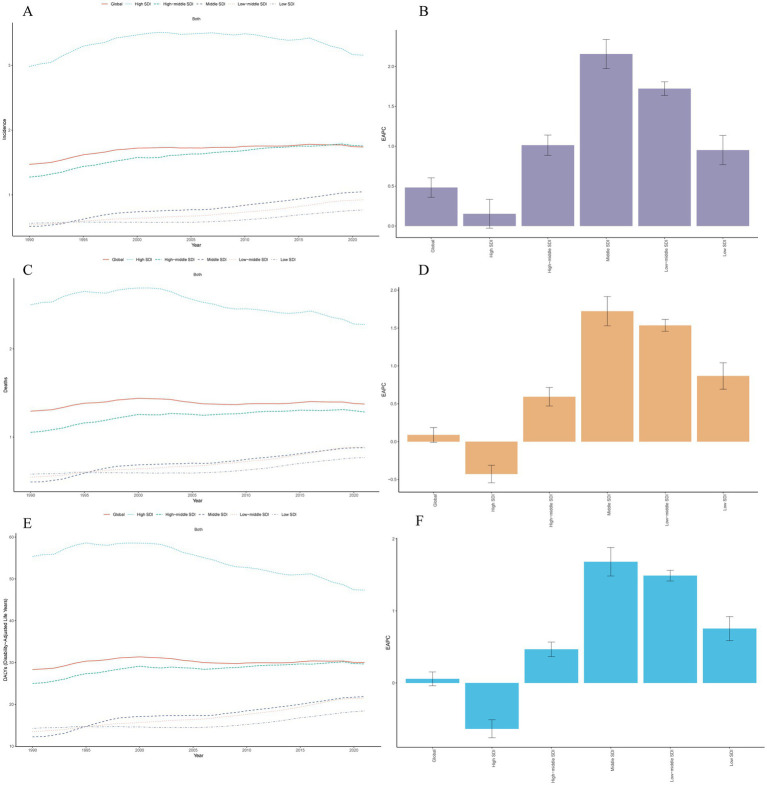
Trends of multiple myeloma in different SDI regions from 1990 to 2021. **(A)** Trends of age-standardized incidence rates. **(B)** EAPC for age-standardized incidence rates. **(C)** Trends of age-standardized mortality rates. **(D)** EAPC for age-standardized mortality rates. **(E)** Trends of age-standardized disability-adjusted life years rates. **(F)** EAPC for age-standardized disability-adjusted life years rates. EAPC, estimated annual percentage change.

The high SDI region had the most incident cases of MM in 2021 (68,288; 95% UI, 61342.09–72525.42). By contrast, the low SDI regions demonstrated significantly lower incident cases (3,801; 95% UI, 2523.31–5130.65), and the ASIR was 0.77 per 100,000 persons (95% UI, 0.51–1.02). But from 1990 to 2021, the greatest increase in the incidence of MM occurred in the middle SDI region (EAPC, 2.15 per 100,000 persons; 95% UI, 1.97–2.34) (Table 1 and Figure 1).

In 2021, the ASMR of MM was found to be highest in regions with a high SDI, recorded at 2.28 per 100,000 persons (95% UI: 2.05–2.41), and lowest in low SDI regions at 0.77 per 100,000 (95% UI: 0.52–1.02). From 1990 to 2021, the significant decline in the ASMR was observed in high SDI regions, with a reduction of 0.43 per 100,000 persons (EAPC, −0.43 per 100,000 persons; 95% UI: −0.54 to −0.31). In contrast, other regions exhibited upward trends (Table 2 and Figure 1).

In 2021, the regions with the highest ASDR were high SDI at 47.33 per 100,000 persons (95% UI: 44.00–49.82), and lowest in low SDI regions at 18.43 per 100,000 (95% UI: 12.25–24.91). It is noteworthy that from 1990 to 2021, the middle SDI experienced the most significant upward trend in ASDR, increasing to 1.68 per 100,000 persons (95% UI: 1.48–1.88), and only one region exhibited downward trend, with high SDI (EAPC, −0.64 per 100,000 persons; 95% UI: −0.76 to −0.51) (Table 3 and Figure 1).

### Geographic regional level

**Figure 2:**
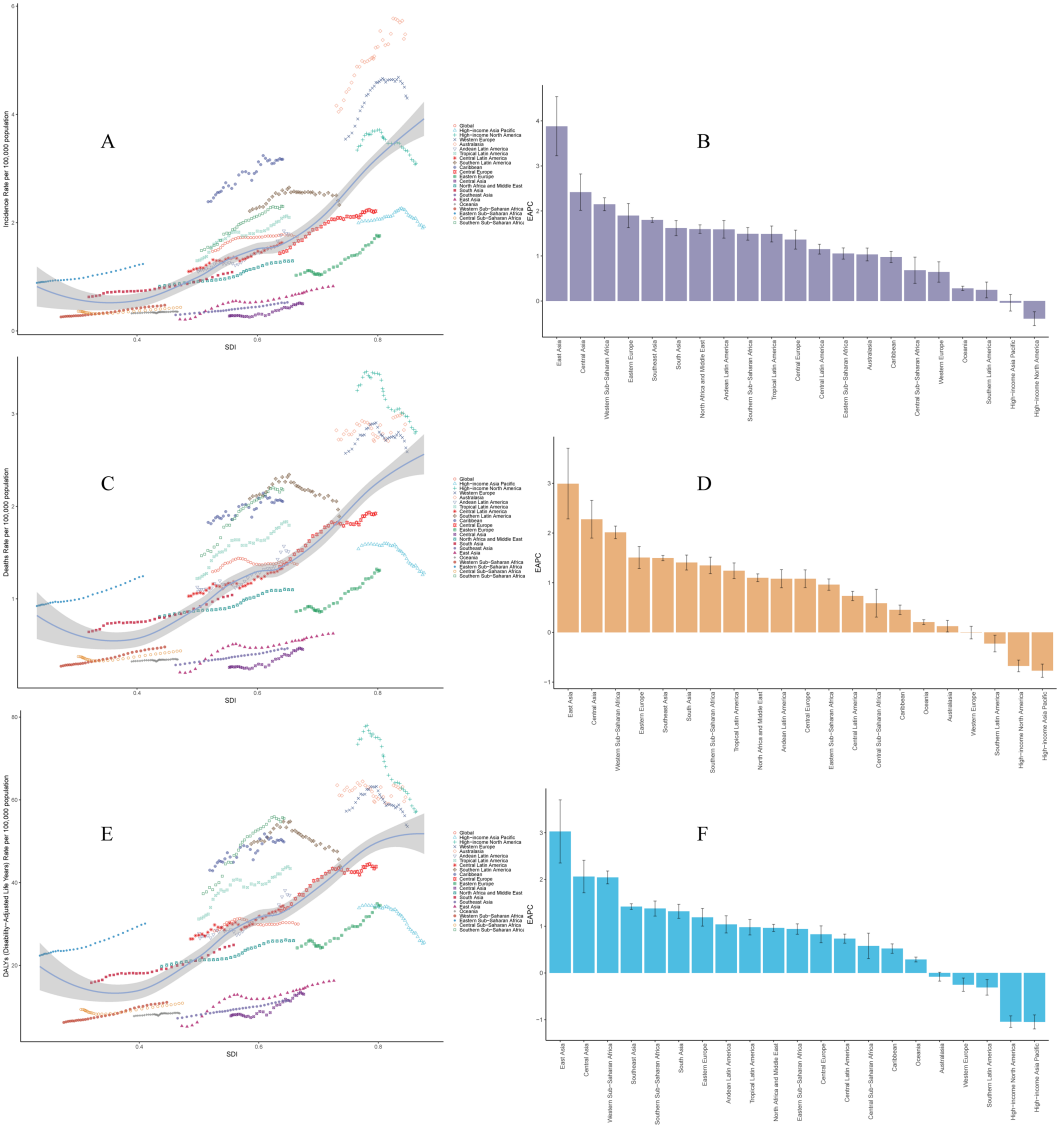
Trends of multiple myeloma in 21 geographic regions from 1990 to 2021. **(A)** Trends of age-standardized incidence rates. **(B)** EAPC for age-standardized incidence rates. **(C)** Trends of age-standardized mortality rates. **(D)** EAPC for age-standardized mortality rates. **(E)** Trends of disability-adjusted life years rates. **(F)** EAPC for age-standardized disability-adjusted life years rates. EAPC, estimated annual percentage change.

Among 21 geographic regions, Western Europe had the most incident cases of MM in 2021 (41,185; 95% UI, 36907.09–44033.08), whereas Oceania had the fewest (26; 95% UI, 14.90–36.97). The ASIR of MM was highest in Australasia (5.48 per 100,000 persons; 95% UI, 4.77–6.21). In contrast, the ASIR of MM was lowest in Oceania (0.36 per 100,000 persons; 95% UI, 0.21–0.50). From 1990 to 2021, East Asia had the largest increase in the incidence of MM (EAPC, 3.88 per 100,000 persons; 95% UI, 3.23–4.54), whereas High-income North America had the largest decrease (EAPC, −0.39 per 100,000 persons; 95% UI, −0.55 to −0.24). In general, the ASIR of MM and SDI levels are positive correlated, as regions with higher SDI levels normally have higher ASIR. From 1990 to 2021, the ASIR of MM in the low SDI regions and middle SDI showed a slight increase, while the high SDI regions such as Western Europe and High-income North America showed an obvious upward trend in the past 10 years, and a significant decline in recent years. However, Australasia was an exception, with much higher than expected levels, and showing a significant increase (Table 1 and Figure 2).

In 2021, Western Europe had the highest number of deaths (26,875; 95% UI, 23758.84–28878.23), whereas Oceania had the lowest number (24; 95% UI, 13.48–34.44). Australasia had the highest ASMR (2.89 per 100,000 persons; 95% UI, 2.52–3.23); Central Sub-Saharan Africa had the lowest ASMR (0.44 per 100,000 persons; 95% UI, 0.23–0.63). From 1990 to 2021, High-income Asia Pacific had the largest decrease in the ASMR (EAPC, −0.77 per 100,000 persons; 95% UI, −0.90 to −0.64); East Asia had the largest increase (EAPC, 2.99 per 100,000 persons; 95% UI, 2.28–3.70). In general, the ASMR of MM and SDI levels are positive correlated, as regions with higher SDI levels normally have higher ASMR (Table 2 and Figure 2). For more information about DALYs, see Table 3 and Figure 2.

### National level

**Figure 3:**
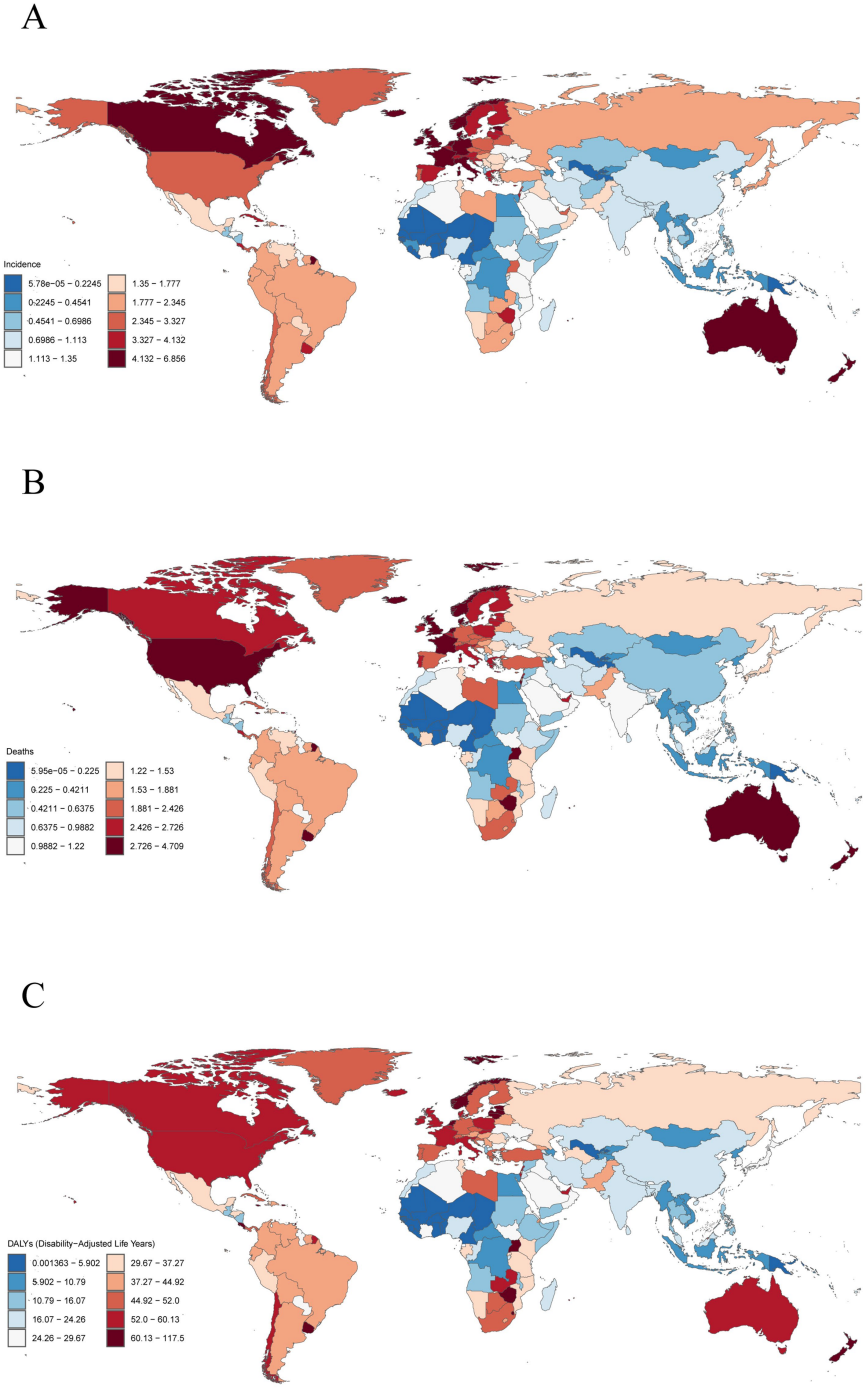
Global distribution of multiple myeloma disease burden in 2021. **(A)** Age-standardized incidence rates. **(B)** Age-standardized mortality rates. **(C)** Age-standardized disability-adjusted life years rates.

In 2021, among 204 countries, Principality of Monaco (6.86 per 100,000 persons; 95% UI: 3.49–10.95), Commonwealth of the Bahamas (6.55 per 100,000 persons; 95% UI: 5.23–8.18) and New Zealand (6.00 per 100,000 persons; 95% UI: 5.19–6.74) have the highest ASIR (Figure 3 and Supplementary Table S1). Notably, the ASIR of Republic of Mali in 2021 was 0 (Figure 3 and Supplementary Table S1). In addition, Republic of Palau (0.05 per 100,000 persons; 95% UI: 0.03–0.08), Republic of the Niger (0.06 per 100,000 persons; 95% UI: 0.02–0.11) and Republic of Kiribati (0.07 per 100,000 persons; 95% UI: 0.04–0.10) exhibited the lowest ASIR (Figure 3 and Supplementary Table S1). From 1990 to 2021, variations in the change of the ASIR were observed across countries (Supplementary Figure S1 and Supplementary Table S2). Georgia (EAPC, 6.20 per 100,000 persons; 95% UI: 5.42–7.00), Turkmenistan (EAPC, 6.12 per 100,000 persons; 95% UI: 5.49–6.74), and Republic of Ghana (EAPC, 4.98 per 100,000 persons; 95% UI: 4.75–5.22) experienced the most substantial relative increases in ASIR (Supplementary Figure S1 and Supplementary Table S2). In contrast, the most pronounced downward trends were observed in Northern Mariana Islands (EAPC, −1.35 per 100,000 persons; 95% UI: −1.61 to −1.08), Republic of Burundi (EAPC, −1.19 per 100,000 persons; 95% UI: −1.40 to −0.98), and Republic of Tajikistan (EAPC, −0.66 per 100,000 persons; 95% UI: −0.93 to −0.38) (Supplementary Figure S1 and Supplementary Table S2). The country-specific distribution of ASMR and ASDR are detailed in Figure 3; Supplementary Figure S1 and Supplementary Tables S3–S6.

### Age and sex differences

**Figure 4:**
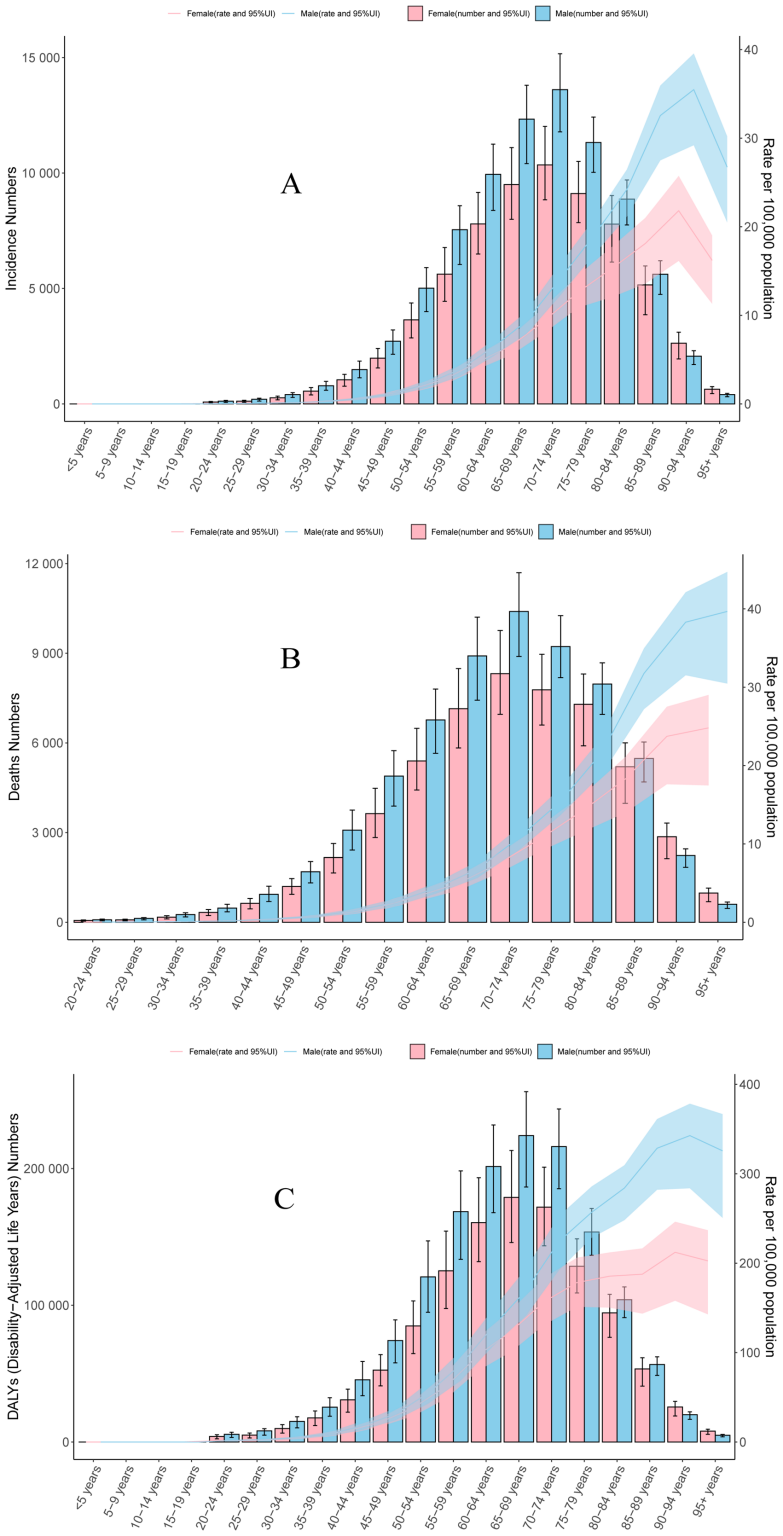
Global distribution of multiple myeloma disease burden by sex and age in 2021. **(A)** Incidence. **(B)** Mortality. **(C)** Disability-adjusted life years.

In 2021, the highest global number of incidence and deaths in MM were observed in people aged 70–74 years. It was worth noting that the incidence and mortality rates were higher in males than in females in all age groups except aged 0–19 years, because the incidence and mortality rates of MM were 0 in people aged 0–19 years. Both men and women had the highest incidence rate of MM in people aged 90–94 years. Incidence rate of MM increased with age before 90 years, and mortality rate increased with age. The DALYs rate analysis revealed a trend similar to that of the incidence, both men and women had the highest DALYs rate of MM in people aged 90–94 years. But the highest global number of DALYs in MM were observed in people aged 65–69 years. Detailed information is provided in Figure 4 and Supplementary Table S7.

Among 21 geographic regions, the incidence rate was higher in males than in females except Caribbean, Western Europe, Southern Sub-Saharan Africa, Central Asia, Oceania, and Western Sub-Saharan Africa, and the mortality rate was higher in males than in females except Eastern Europe, Southern Sub-Saharan Africa, Central Asia, Oceania, and Western Sub-Saharan Africa (Supplementary Figure S2 and Supplementary Table S8). The DALYs rate was higher in males than in females except Eastern Europe, Southern Sub-Saharan Africa, Oceania, and Western Sub-Saharan Africa (Supplementary Figure S2 and Supplementary Table S8).

### Risk factors

**Figure 5:**
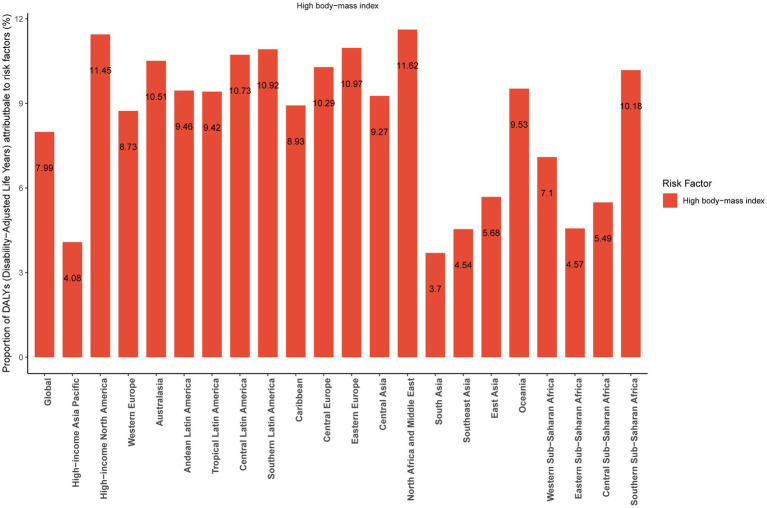
The percentage of disability-adjusted life years attributable to high body-mass index in each region, 2021.

In 2021, the percentage of DALYs attributable to high body-mass index in each region is shown in Figure 5. Globally, the percentage of DALYs attributable to high body-mass index was 7.99%. Among 21 geographic regions, high body-mass index significantly contributed to DALYs, with the highest proportion in North Africa and Middle East at 11.62%, and the lowest in South Asia at 3.7%.

### Future forecasts of global burden

**Figure 6:**
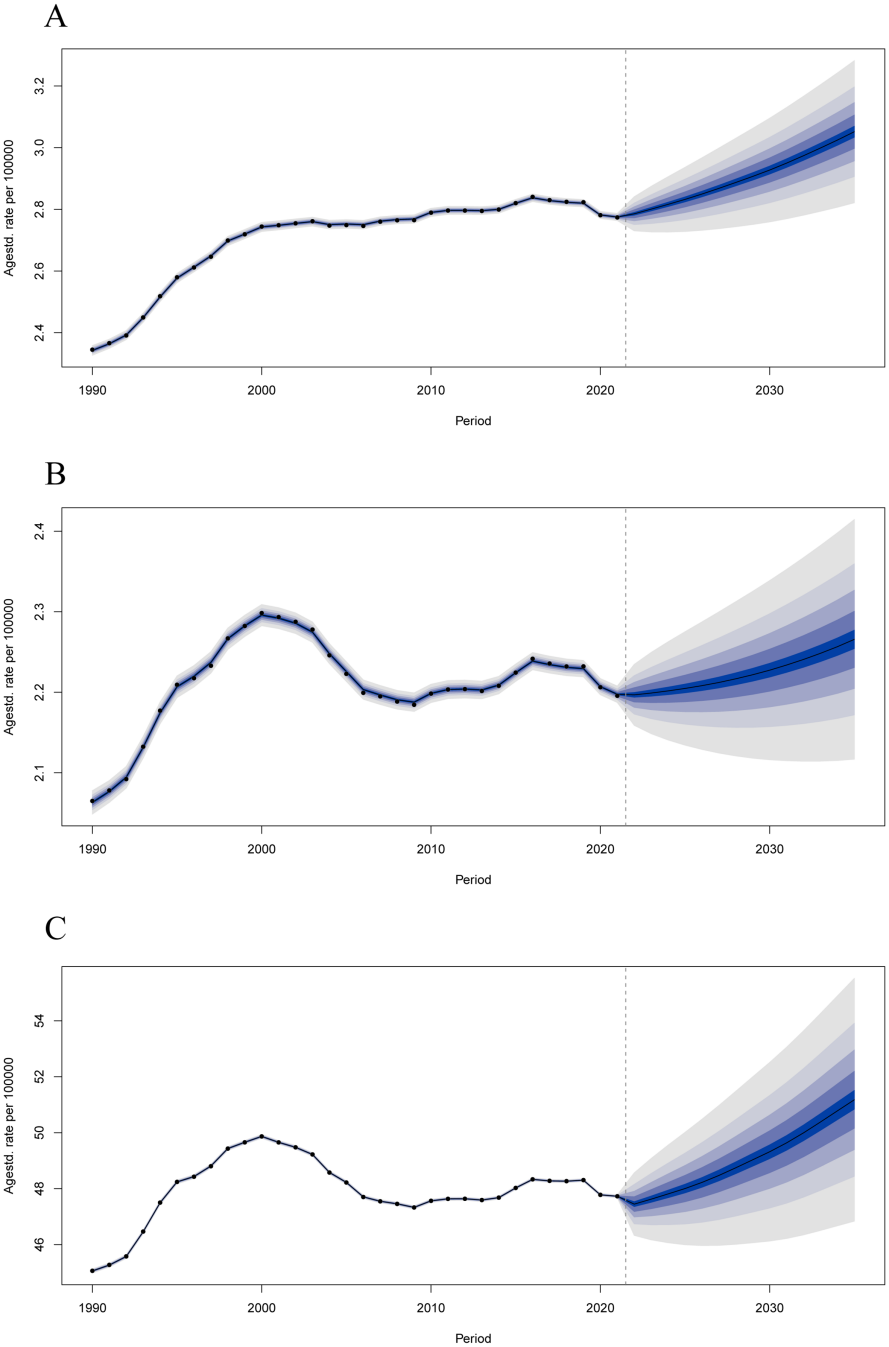
Predictions of the global burden of multiple myeloma disease by Bayesian age-period-cohort (BAPC) model in 2021–2035. **(A)** Age-standardized incidence rates. **(B)** Age-standardized mortality rates. **(C)** Age-standardized disability-adjusted life years rates.

As presented in Figure 6, we projected that the ASIR, ASMR, and ASDR of MM would obviously increase over the next dozen years. ASIR would continuous increase to 3.05 per 100,000 (95% UI 2.78 to 3.33) in 2035 (Supplementary Table S9); ASMR would continuous increase 2.27 per 100,000 (95% UI 2.09 to 2.44) in 2035 (Supplementary Table S10); and ASDR would continuous increase to 51.18 per 100,000 (95% UI 46.01 to 56.35) in 2035 (Supplementary Table S11). In addition, the ASIR (Supplementary Table S12), ASMR (Supplementary Table S13) and ASDR (Supplementary Table S14) of MM in both males and females would obviously increase over the next dozen years, with the rate higher in men than in women (Supplementary Figure S3).

## Discussion

To our knowledge, this study is the latest assessment of the global burden of MM based on GBD Study 2021. In this study, we show the most comprehensive and up-to-date information of the global burden of MM. It should be noted that for our study, we opted for age-standardized rates to compare relative burden levels, rather than crude rates. The raw absolute burden levels indicated by the crude rates are detailed in Supplementary Table S15. On a global scale, the ASIR, ASMR, and ASDR of MM increased from 1990 to 2021. These findings align with previous GBD studies (11, 12). Among the geographic regions, the highest ASIR, ASMR, and ASDR were observed in regions with high SDI, while the lowest rates were found in low SDI regions. In general, the ASIR, ASMR, and ASDR of MM and SDI levels are positive correlated, as regions with higher SDI levels normally have higher ASIR, ASMR, and ASDR. This finding highlights the persistent socioeconomic disparities in the global burden of MM (20). The high burden in areas with high SDI may be due to a combination of factors, including suboptimal risk factor management and inadequate secondary prevention strategies (21). Therefore, health policy makers should pay more attention to socio-economic related factors and take personalized measures in different SDI regions. Based on our findings, regions with high SDI levels should prioritize integrating MM screening within existing non-communicable disease programs and invest in diagnostic infrastructure, focus on strengthening access to affordable novel therapies and palliative care. It is noteworthy that low-income populations may experience earlier mortality due to delayed diagnosis and inadequate treatment, resulting in underrepresentation of recorded MM cases. This introduces potential errors in MM incidence or mortality statistics. Crucially, age standardization only adjusts for age distribution and cannot eliminate this survivorship bias. As our study relies on secondary data, results may be influenced by diagnostic completeness disparities—particularly in regions with low SDI. Future research should integrate mortality registries with pathology databases to validate and correct this bias.

In 2021, among 204 countries, Principality of Monaco, Commonwealth of the Bahamas, and New Zealand have the highest ASIR. However, the ASIR of Republic of Mali in 2021 was closed to zero, which may be significant to prevent for MM. In addition, from 1990 to 2021, Georgia, Turkmenistan, and Republic of Ghana experienced the most substantial increases in ASIR, and an in-depth understanding of the reasons for this phenomenon may be significant to explore the risk factors for MM.

The study also found that the incidence and mortality rates of MM were 0 in people aged 0–19 years. The incidence rates and mortality rates were higher in males than in females in all age groups. This was consistent with some previous reports (22–24). But we also found something different, among Southern Sub-Saharan Africa, Central Asia, Oceania, and Western Sub-Saharan Africa, the incidence and mortality rates were higher in females than in males. This may be related to local social factors. In addition, we also found that incidence and mortality rates of MM increased with age before 90 years. Based on the research results, healthcare policymakers and stakeholders should enhance the early screening and diagnosis of MM diseases in the elderly population, especially among male elderly individuals.

A pervious study showed that high body-mass index was associated with an increased risk of MM mortality independent of smoking or alcohol consumption among Asian populations (25). This study focuses specifically on high body-mass index due to its significant population attributable fraction, established causal link with multiple myeloma risk in epidemiological studies, and critical importance as a modifiable target for public health intervention aimed at reducing future disease burden. In addition, there were few risk factors associated with MM in the 2021 GBD Study, so we only chose high body-mass index as a risk factor for MM to analyze. This study show that the percentage of DALYs attributable to high body-mass index was 7.99% globally in 2021. The pathophysiological pathways linking high body-mass index to MM are very complicated and include adipocytes; inflammatory cytokines and growth factors at all (26–29). One study found that adipocytes from patients with high body-mass index significantly increased MM cell adhesion and expression of matrix metalloproteinase-2 relative to adipocytes derived from patients with normal body-mass index, thus contributing to MM growth and progression (27). North Africa and Middle East had the highest percentage of DALYs attributable to high body-mass index among 21 geographic regions. This may be related to factors such as access to cancer drugs, healthcare access and healthcare expenditures (30). Furthermore, high body mass index is more prevalent in high-income North America, Eastern Europe, and Southern Latin America. This highlights the urgent need to strengthen obesity prevention and management, and to implement targeted public health strategies to mitigate the impact of obesity on MM. However, robust population-level data on the global attributable burden for other risk factors remain limited compared to high body-mass index. Future studies incorporating multi-risk factor assessments would be valuable to provide a more comprehensive picture of preventable burden.

We had also made future projections of the global burden of MM through BAPC model. To minimize overfitting or imprecision, we ensured that each age-period stratum had sufficient sample size (*n* ≥ 5). Convergence diagnostics indicated that all chains reached stable posterior distributions (R-hat <1.05; ESS >1,000), supporting the reliability of model estimates. As a forecasting model, the rationality of BAPC model has been verified (31–33). The results showed that the ASIR, ASMR, and ASDR of MM in global would obviously increase over the next dozen years, which can help in health policy planning and resource allocation. It’s worth noting that the advent of an aging society will make matters worse. In addition, the ASIR, ASMR, and ASDR of MM in males than in females in the future. These findings provide evidence-based guidance for formulating rational MM health management strategies and allocating health resources. For example, targeting high BMI to percent incidence through population-level weight management programs, and implementing early screening, diagnosis, and treatment to decrease mortality. The forecasted demographic shifts highlight the need for health systems in aging populations to plan for increased demand for hematological oncology services and specialized geriatric care.

Although this study show the most comprehensive and up-to-date information of the global burden of MM based on GBD Study 2021, the study still has several limitations. First, all the data used in our study came from GBD 2021, which come from different countries and regions. As a result, there may be inconsistencies between these data, which can lead to bias. Second, there were few risk factors associated with MM in the 2021 GBD Study, so we only chose high body-mass index as a risk factor for MM to analyze the percentage of DALYs in each region. It is not possible to identify risk factors suitable for each region and country, which may be more important to reduce the global burden of MM.

## Conclusion

In summary, we provide the most comprehensive and up-to-date information about the global burden of MM. Globally, the total burden of MM increased from 1990 to 2021 and the ASIR, ASMR, and ASDR are predicted to continuous increase until 2035. As a result, the global burden of MM will continue to increase, and countries should adopt broader and more effective approaches to prevent and treat MM.

## Data Availability

The original contributions presented in the study are included in the article/Supplementary material, further inquiries can be directed to the corresponding authors.

## Footnotes

^1^ http://ghdx.healthdata.org/gbd-results-tool
