# Supplementary material for: Global, regional, and national burden of multiple myeloma, 1990 to 2021 and predictions to 2035: an analysis of the Global Burden of Disease Study 2021
Source: Front Med (Lausanne). 2025 Jul 31;12:1609692. doi: 10.3389/fmed.2025.1609692 (PMC12350341; doi:10.3389/fmed.2025.1609692)
Supplement: Supplementary file 1 [file Data_Sheet_1.docx]

**Supplementary Online Content**

**Supplementary Figure S1.** Global distribution of multiple myeloma disease burden from 1990 to 2021. (A)EAPC for age-standardized incidence rates; (B)EAPC for age-standardized mortality rates; (C)EAPC for age-standardized disability-adjusted life years rates; EAPC = estimated annual percentage change.

**Supplementary Figure S2.** Sex differences in multiple myeloma across 21 geographic regions in 2021. (A) Incidence rates; (B) Mortality rates; (C) Disability-adjusted life years rates.

**Supplementary Figure S3.** Prediction of the gender-specific global burden of multiple myeloma disease in 2021-2035 based on a Bayesian Age-time-cohort (BAPC) model. (A) Trends of age-standardized incidence rates in males; (B) Trends of age-standardized incidence rates in females; (C) Trends of age-standardized mortality rates in males; (D) Trends of age-standardized mortality rates in females; (E) Trends of age-standardized disability-adjusted life years rates in males; (F) Trends of age-standardized disability-adjusted life years rates in females.

**Supplementary Table S1.** Age-standardized incidence rates of multiple myeloma for both sex by Country, 2021.

**Supplementary Table S2.** Estimated annual percentage change (EAPC) in age-standardized incidence rates of multiple myeloma for both sex by Country, 1990-2021.

**Supplementary Table S3.** Age-standardized mortality rates of multiple myeloma for both sex by Country, 2021.

**Supplementary Table S4.** Estimated annual percentage change (EAPC) in age-standardized mortality rates of multiple myeloma for both sex by Country, 1990-2021.

**Supplementary Table S5.** Age-standardized disability-adjusted life years(DALYs) rates of multiple myeloma for both sex by Country, 2021.

**Supplementary Table S6.** Estimated annual percentage change (EAPC) in disability-adjusted life years(DALYs) rates of multiple myeloma for both sex by Country, 1990-2021.

**Supplementary Table S7.** Global age and sex structure of multiple myeloma incidence, mortality and disability-adjusted life years, 2021.

**Supplementary Table S8.** Sex differences of incidence, mortality and disability-adjusted life years rates in multiple myeloma across 21 geographic regions in 2021.

**Supplementary Table S9.** Predictions of the age-standardized incidence rates of multiple myeloma by Bayesian Age-Period-Cohort (BAPC) model.

**Supplementary Table S10.** Predictions of the age-standardized mortality rates of multiple myeloma by Bayesian Age-Period-Cohort (BAPC) model.

**Supplementary Table S11.** Predictions of the age-standardized disability-adjusted life years rates of multiple myeloma by Bayesian Age-Period-Cohort (BAPC) model.

**Supplementary Table S12.** Predictions of the age-standardized incidence rates of multiple myeloma in diffent sex by Bayesian Age-Period-Cohort (BAPC) model.

**Supplementary Table S13.** Predictions of the age-standardized mortality rates of multiple myeloma in diffent sex by Bayesian Age-Period-Cohort (BAPC) model.

**Supplementary Table S14.** Predictions of the age-standardized disability-adjusted life years rates of multiple myeloma in diffent sex by Bayesian Age-Period-Cohort (BAPC) model.

**Supplementary Table S15.** All ages incidence, mortality and DALYs rate per 100 000 persons of MM in 1990 and 2021, along with EAPC per 100 000 persons from 1990 to 2021, categorized by global, SDI, and GBD regions.


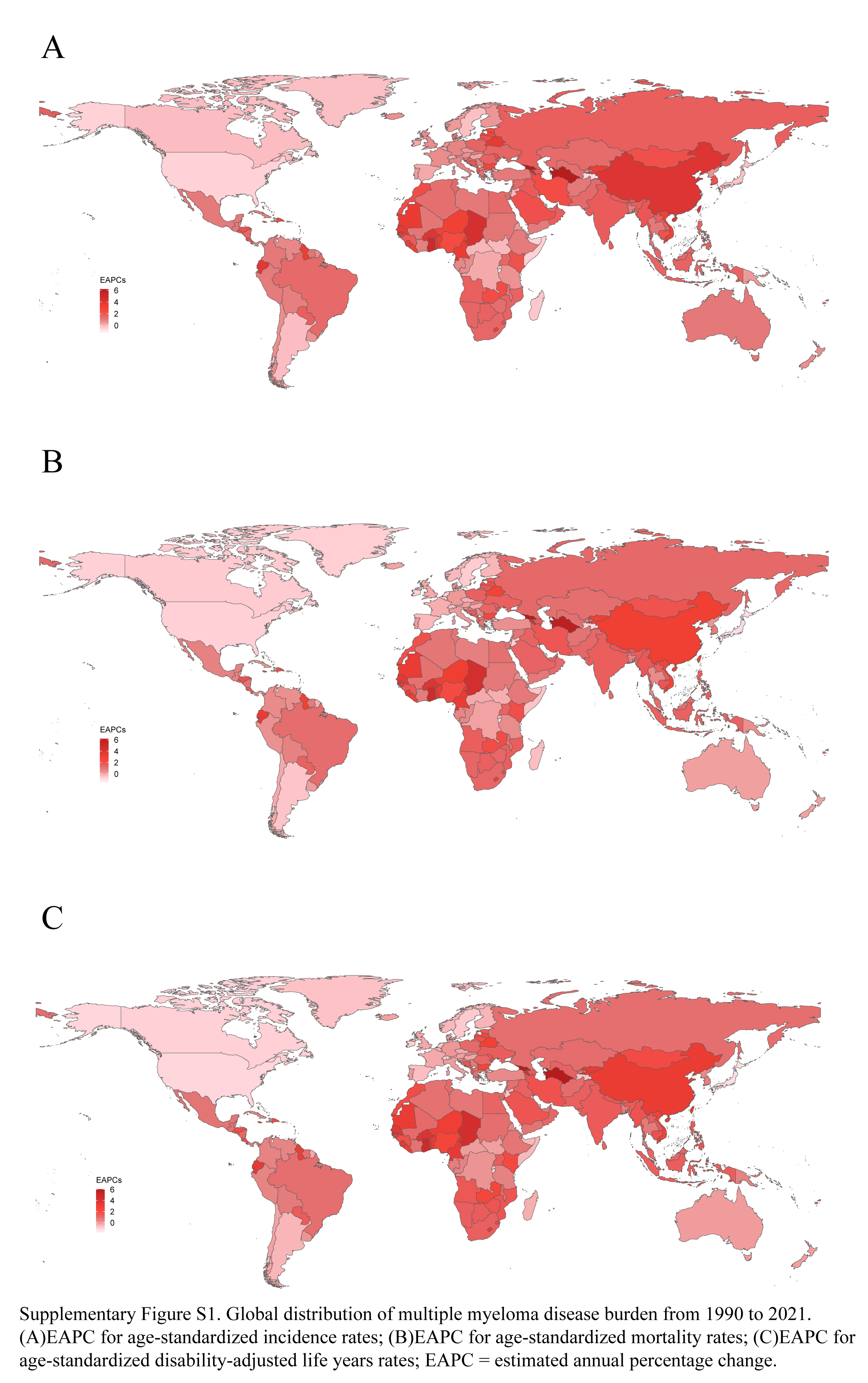


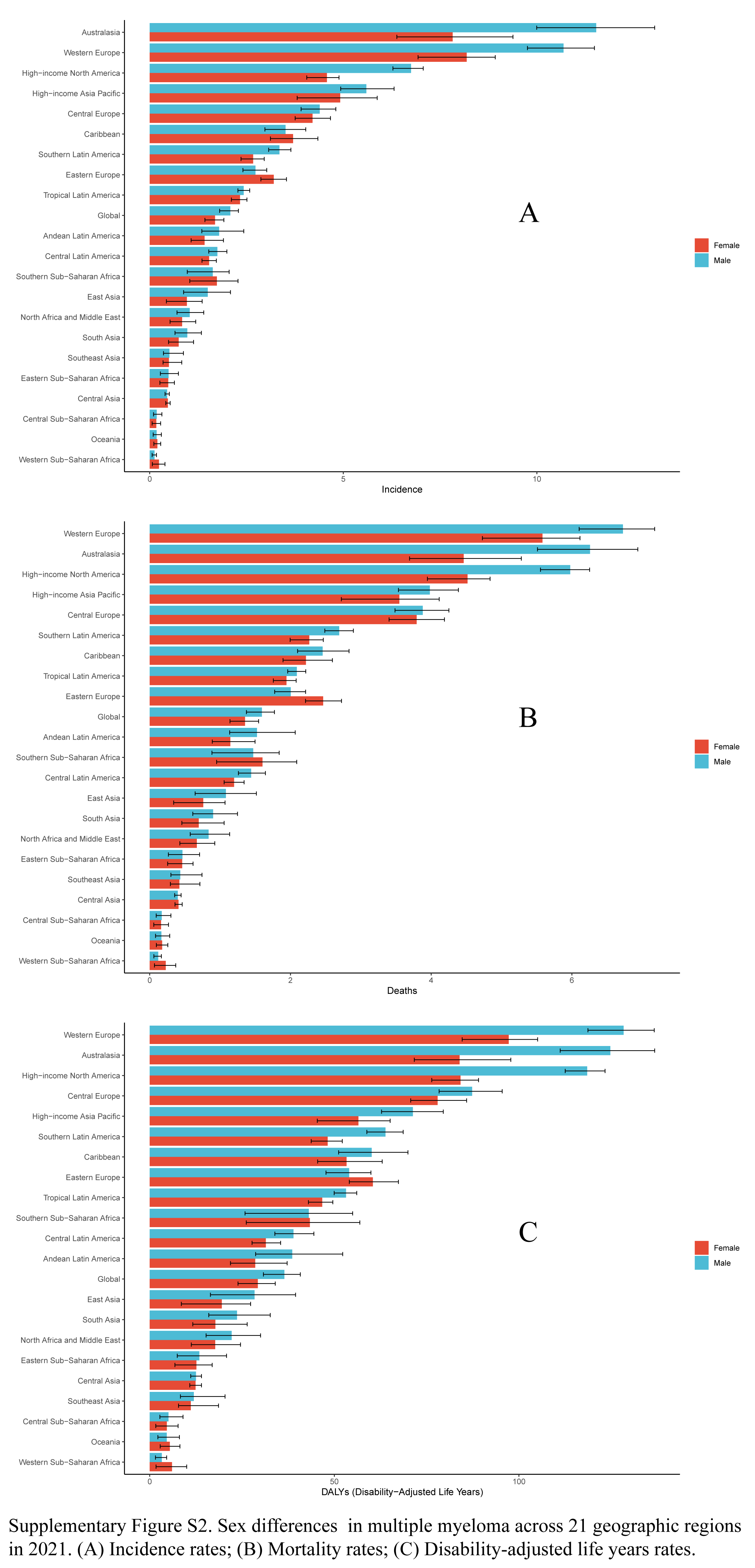


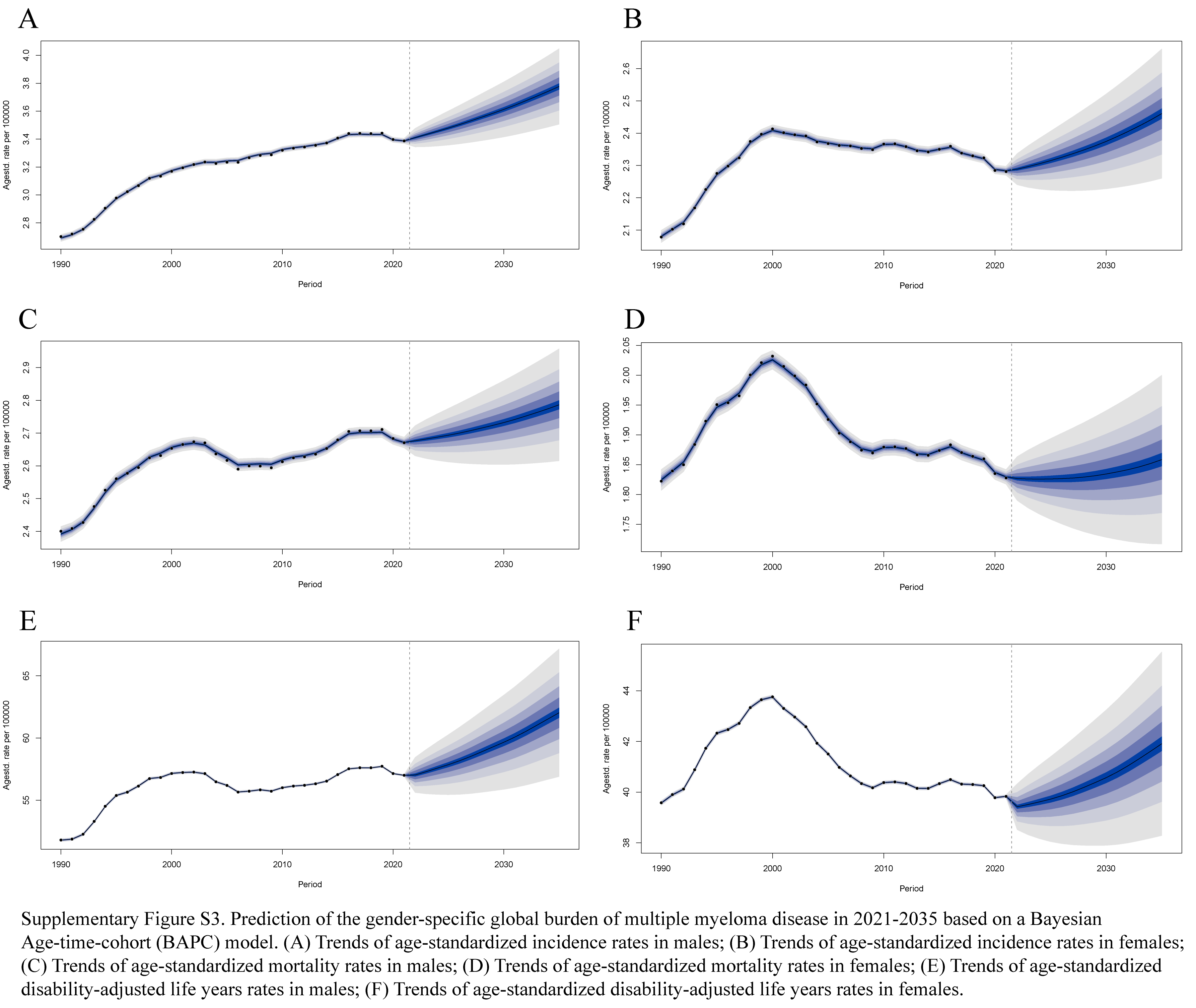


Supplementary Table S1. Age-standardized incidence rates of multiple myeloma for both sex by Country, 2021.

| location | val | upper | lower |
| --- | --- | --- | --- |
| Principality of Monaco | 6.86 | 10.95 | 3.49 |
| Commonwealth of the Bahamas | 6.55 | 8.18 | 5.23 |
| New Zealand | 6.00 | 6.74 | 5.19 |
| Kingdom of Norway | 5.55 | 6.10 | 4.91 |
| Australia | 5.38 | 6.21 | 4.61 |
| United Kingdom of Great Britain and Northern Ireland | 5.23 | 5.48 | 4.82 |
| Jamaica | 4.97 | 6.51 | 3.82 |
| Bermuda | 4.93 | 6.36 | 4.06 |
| Grenada | 4.81 | 5.54 | 4.13 |
| Puerto Rico | 4.69 | 5.65 | 3.83 |
| Republic of Estonia | 4.59 | 5.43 | 3.84 |
| Saint Lucia | 4.57 | 5.56 | 3.66 |
| Republic of Iceland | 4.56 | 5.25 | 3.92 |
| Republic of Italy | 4.55 | 5.05 | 4.04 |
| Kingdom of Denmark | 4.49 | 5.06 | 3.93 |
| Kingdom of the Netherlands | 4.45 | 4.94 | 3.93 |
| Federal Republic of Germany | 4.34 | 4.79 | 3.83 |
| Canada | 4.32 | 4.87 | 3.80 |
| Antigua and Barbuda | 4.24 | 4.79 | 3.81 |
| Ireland | 4.18 | 4.79 | 3.56 |
| French Republic | 4.14 | 4.81 | 3.57 |
| Republic of Finland | 4.12 | 4.61 | 3.61 |
| Swiss Confederation | 4.11 | 4.70 | 3.52 |
| State of Israel | 4.05 | 4.55 | 3.55 |
| Kingdom of Belgium | 4.00 | 4.52 | 3.49 |
| Kingdom of Spain | 3.87 | 4.37 | 3.36 |
| Republic of Trinidad and Tobago | 3.85 | 4.95 | 2.88 |
| Grand Duchy of Luxembourg | 3.79 | 4.30 | 3.30 |
| Republic of Lithuania | 3.75 | 4.37 | 3.21 |
| Saint Kitts and Nevis | 3.73 | 4.51 | 3.02 |
| Hellenic Republic | 3.58 | 3.97 | 3.23 |
| Eastern Republic of Uruguay | 3.58 | 3.97 | 3.24 |
| Republic of Cyprus | 3.55 | 4.71 | 2.33 |
| Republic of Cuba | 3.55 | 4.14 | 3.03 |
| Republic of Zimbabwe | 3.51 | 5.05 | 1.94 |
| Kingdom of Sweden | 3.49 | 4.01 | 3.04 |
| Republic of Latvia | 3.47 | 4.14 | 2.91 |
| Republic of Slovenia | 3.44 | 4.04 | 2.88 |
| Republic of Croatia | 3.42 | 4.11 | 2.85 |
| Republic of Austria | 3.37 | 3.80 | 2.94 |
| Republic of Costa Rica | 3.34 | 3.82 | 2.84 |
| Slovak Republic | 3.31 | 4.46 | 2.27 |
| United States Virgin Islands | 3.29 | 5.18 | 2.03 |
| Commonwealth of Dominica | 3.19 | 4.46 | 2.14 |
| Lebanese Republic | 3.15 | 4.26 | 2.16 |
| Barbados | 3.12 | 4.09 | 2.33 |
| Republic of Uganda | 3.08 | 4.57 | 2.16 |
| Czech Republic | 3.04 | 3.55 | 2.55 |
| Republic of Chile | 3.01 | 3.34 | 2.71 |
| United States of America | 2.95 | 3.09 | 2.70 |
| United Arab Emirates | 2.94 | 3.93 | 2.06 |
| Republic of Malta | 2.75 | 3.18 | 2.34 |
| Republic of Poland | 2.60 | 2.83 | 2.34 |
| Portuguese Republic | 2.55 | 2.83 | 2.26 |
| Principality of Andorra | 2.51 | 3.66 | 1.64 |
| Greenland | 2.51 | 3.67 | 1.85 |
| Republic of Belarus | 2.49 | 3.07 | 2.00 |
| Republic of Panama | 2.48 | 3.05 | 1.98 |
| Kingdom of Eswatini | 2.43 | 3.59 | 1.62 |
| Republic of San Marino | 2.42 | 3.74 | 1.47 |
| Brunei Darussalam | 2.39 | 3.18 | 1.68 |
| Republic of Zambia | 2.34 | 4.00 | 1.03 |
| Republic of Turkey | 2.33 | 3.21 | 1.69 |
| Saint Vincent and the Grenadines | 2.31 | 2.67 | 2.01 |
| Kingdom of Bahrain | 2.24 | 3.18 | 1.36 |
| State of Libya | 2.23 | 3.09 | 1.42 |
| Republic of South Africa | 2.18 | 2.67 | 1.41 |
| Republic of Suriname | 2.17 | 3.01 | 1.49 |
| Federative Republic of Brazil | 2.11 | 2.23 | 1.96 |
| Republic of Mauritius | 1.99 | 2.16 | 1.82 |
| Republic of Colombia | 1.99 | 2.39 | 1.64 |
| Taiwan (Province of China) | 1.98 | 2.24 | 1.75 |
| Japan | 1.97 | 2.17 | 1.72 |
| Hungary | 1.97 | 2.29 | 1.68 |
| Dominican Republic | 1.96 | 2.68 | 1.37 |
| Republic of Botswana | 1.93 | 2.82 | 1.35 |
| Argentine Republic | 1.90 | 2.07 | 1.72 |
| Kingdom of Tonga | 1.84 | 2.57 | 1.27 |
| Republic of Peru | 1.81 | 2.58 | 1.26 |
| Russian Federation | 1.81 | 1.96 | 1.66 |
| Plurinational State of Bolivia | 1.79 | 2.67 | 1.23 |
| Republic of Ecuador | 1.78 | 2.28 | 1.40 |
| Palestine | 1.77 | 2.33 | 1.06 |
| Republic of Korea | 1.76 | 2.32 | 1.01 |
| Bolivarian Republic of Venezuela | 1.73 | 2.28 | 1.27 |
| Republic of Seychelles | 1.72 | 2.26 | 1.29 |
| Islamic Republic of Pakistan | 1.71 | 2.46 | 1.20 |
| Kingdom of Lesotho | 1.69 | 2.59 | 1.09 |
| Republic of Tunisia | 1.66 | 2.42 | 1.05 |
| Republic of Serbia | 1.62 | 2.14 | 1.15 |
| Republic of Djibouti | 1.59 | 2.39 | 0.91 |
| United Mexican States | 1.55 | 1.74 | 1.37 |
| Republic of Bulgaria | 1.49 | 1.95 | 1.17 |
| Georgia | 1.48 | 1.73 | 1.26 |
| State of Qatar | 1.48 | 2.38 | 0.93 |
| Romania | 1.48 | 1.68 | 1.28 |
| Sultanate of Oman | 1.47 | 2.02 | 0.99 |
| Republic of Namibia | 1.43 | 1.98 | 0.99 |
| Republic of Iraq | 1.39 | 1.98 | 0.92 |
| Republic of Paraguay | 1.38 | 2.00 | 0.91 |
| Republic of Haiti | 1.36 | 2.55 | 0.76 |
| Republic of Rwanda | 1.35 | 1.98 | 0.79 |
| United Republic of Tanzania | 1.35 | 1.98 | 0.78 |
| Union of the Comoros | 1.33 | 1.95 | 0.72 |
| Republic of Kenya | 1.33 | 1.81 | 0.85 |
| Republic of Guyana | 1.32 | 1.71 | 1.02 |
| People's Democratic Republic of Algeria | 1.30 | 1.79 | 0.81 |
| Bosnia and Herzegovina | 1.29 | 1.72 | 0.87 |
| Northern Mariana Islands | 1.29 | 1.86 | 1.00 |
| Gabonese Republic | 1.28 | 1.84 | 0.72 |
| Montenegro | 1.28 | 1.69 | 0.89 |
| Kingdom of Saudi Arabia | 1.27 | 1.71 | 0.79 |
| Republic of Honduras | 1.27 | 1.87 | 0.83 |
| Republic of Mozambique | 1.25 | 1.98 | 0.66 |
| Republic of Cote d'Ivoire | 1.24 | 1.77 | 0.82 |
| American Samoa | 1.20 | 1.61 | 0.86 |
| Democratic Socialist Republic of Sri Lanka | 1.19 | 1.83 | 0.74 |
| Ukraine | 1.19 | 1.55 | 0.88 |
| Hashemite Kingdom of Jordan | 1.19 | 1.75 | 0.75 |
| State of Eritrea | 1.16 | 1.73 | 0.61 |
| Republic of Moldova | 1.13 | 1.30 | 0.99 |
| Republic of South Sudan | 1.11 | 1.74 | 0.59 |
| State of Kuwait | 1.11 | 1.33 | 0.90 |
| Kingdom of Bhutan | 1.09 | 1.99 | 0.56 |
| Turkmenistan | 1.09 | 1.48 | 0.82 |
| Republic of Singapore | 1.07 | 1.21 | 0.94 |
| Belize | 1.07 | 1.23 | 0.93 |
| North Macedonia | 1.06 | 1.41 | 0.69 |
| Republic of India | 1.06 | 1.40 | 0.83 |
| Republic of Equatorial Guinea | 1.03 | 1.62 | 0.59 |
| Malaysia | 1.02 | 1.49 | 0.81 |
| Islamic Republic of Iran | 0.98 | 1.21 | 0.63 |
| Republic of Armenia | 0.96 | 1.17 | 0.80 |
| Kingdom of Morocco | 0.90 | 1.42 | 0.55 |
| Republic of Fiji | 0.88 | 1.20 | 0.58 |
| Republic of Madagascar | 0.85 | 1.25 | 0.45 |
| Republic of Burundi | 0.82 | 1.55 | 0.39 |
| Federal Democratic Republic of Nepal | 0.81 | 1.36 | 0.43 |
| People's Republic of China | 0.81 | 1.07 | 0.52 |
| People's Republic of Bangladesh | 0.80 | 1.38 | 0.49 |
| Federal Republic of Nigeria | 0.77 | 1.20 | 0.28 |
| Republic of the Congo | 0.72 | 1.07 | 0.46 |
| Kingdom of Thailand | 0.70 | 1.31 | 0.43 |
| Republic of Kazakhstan | 0.69 | 0.81 | 0.57 |
| Republic of El Salvador | 0.68 | 0.90 | 0.46 |
| Federal Democratic Republic of Ethiopia | 0.66 | 1.17 | 0.34 |
| Islamic Republic of Afghanistan | 0.65 | 1.37 | 0.27 |
| Republic of Maldives | 0.64 | 0.89 | 0.44 |
| Independent State of Samoa | 0.63 | 1.00 | 0.43 |
| Republic of Sudan | 0.62 | 1.01 | 0.37 |
| Republic of Malawi | 0.60 | 0.85 | 0.37 |
| Republic of Angola | 0.59 | 0.84 | 0.36 |
| Republic of Niue | 0.59 | 0.83 | 0.31 |
| Republic of Nauru | 0.58 | 1.05 | 0.28 |
| Guam | 0.58 | 0.80 | 0.43 |
| Republic of Albania | 0.54 | 0.76 | 0.35 |
| Federal Republic of Somalia | 0.53 | 1.12 | 0.23 |
| Syrian Arab Republic | 0.53 | 0.78 | 0.33 |
| Republic of Guatemala | 0.53 | 0.62 | 0.45 |
| Republic of Nicaragua | 0.53 | 0.70 | 0.38 |
| Republic of Yemen | 0.49 | 0.88 | 0.27 |
| Kingdom of Cambodia | 0.46 | 0.96 | 0.26 |
| Republic of the Philippines | 0.46 | 0.63 | 0.34 |
| Republic of Indonesia | 0.44 | 0.84 | 0.28 |
| Tokelau | 0.44 | 0.64 | 0.21 |
| Arab Republic of Egypt | 0.43 | 0.81 | 0.28 |
| Lao People's Democratic Republic | 0.41 | 0.82 | 0.22 |
| Federated States of Micronesia | 0.39 | 0.62 | 0.20 |
| Republic of the Union of Myanmar | 0.39 | 0.75 | 0.24 |
| Central African Republic | 0.36 | 0.55 | 0.19 |
| Tuvalu | 0.36 | 0.53 | 0.19 |
| Republic of the Marshall Islands | 0.36 | 0.61 | 0.17 |
| Democratic People's Republic of Korea | 0.34 | 0.58 | 0.19 |
| Democratic Republic of the Congo | 0.34 | 0.57 | 0.13 |
| Cook Islands | 0.33 | 0.44 | 0.23 |
| Socialist Republic of Viet Nam | 0.32 | 0.42 | 0.21 |
| Kyrgyz Republic | 0.31 | 0.40 | 0.24 |
| Mongolia | 0.30 | 0.41 | 0.21 |
| Republic of Vanuatu | 0.30 | 0.49 | 0.14 |
| Republic of Azerbaijan | 0.27 | 0.40 | 0.17 |
| Democratic Republic of Timor-Leste | 0.26 | 0.52 | 0.16 |
| Solomon Islands | 0.26 | 0.46 | 0.12 |
| Republic of Guinea | 0.23 | 0.36 | 0.13 |
| Independent State of Papua New Guinea | 0.22 | 0.41 | 0.09 |
| Republic of Tajikistan | 0.22 | 0.31 | 0.14 |
| Republic of Uzbekistan | 0.21 | 0.26 | 0.16 |
| Republic of Cabo Verde | 0.14 | 0.23 | 0.05 |
| Republic of Cameroon | 0.13 | 0.21 | 0.04 |
| Republic of Ghana | 0.13 | 0.25 | 0.04 |
| Republic of the Gambia | 0.12 | 0.18 | 0.07 |
| Islamic Republic of Mauritania | 0.12 | 0.20 | 0.04 |
| Democratic Republic of Sao Tome and Principe | 0.11 | 0.18 | 0.04 |
| Republic of Guinea-Bissau | 0.10 | 0.16 | 0.04 |
| Republic of Senegal | 0.10 | 0.17 | 0.03 |
| Burkina Faso | 0.09 | 0.15 | 0.03 |
| Togolese Republic | 0.09 | 0.15 | 0.03 |
| Republic of Benin | 0.09 | 0.14 | 0.03 |
| Republic of Liberia | 0.08 | 0.15 | 0.02 |
| Republic of Chad | 0.08 | 0.12 | 0.03 |
| Republic of Sierra Leone | 0.07 | 0.12 | 0.02 |
| Republic of Kiribati | 0.07 | 0.10 | 0.04 |
| Republic of the Niger | 0.06 | 0.11 | 0.02 |
| Republic of Palau | 0.05 | 0.08 | 0.03 |
| Republic of Mali | 0.00 | 0.00 | 0.00 |

Supplementary Table S2. Estimated annual percentage change (EAPC) in age-standardized incidence rates of multiple myeloma for both sex by Country, 1990-2021.

| location | val | upper | lower |
| --- | --- | --- | --- |
| Georgia | 6.20 | 7.00 | 5.42 |
| Turkmenistan | 6.12 | 6.74 | 5.49 |
| Republic of Ghana | 4.98 | 5.22 | 4.75 |
| Democratic Republic of Sao Tome and Principe | 4.65 | 4.85 | 4.45 |
| Republic of Armenia | 4.51 | 5.19 | 3.84 |
| Republic of Chad | 4.44 | 4.70 | 4.19 |
| People's Republic of China | 4.05 | 4.76 | 3.35 |
| Republic of Equatorial Guinea | 4.03 | 4.27 | 3.78 |
| Burkina Faso | 3.99 | 4.15 | 3.82 |
| Republic of Ecuador | 3.80 | 4.13 | 3.47 |
| Togolese Republic | 3.67 | 3.83 | 3.52 |
| Republic of Senegal | 3.67 | 3.86 | 3.48 |
| Republic of Mauritius | 3.59 | 4.53 | 2.67 |
| Republic of Belarus | 3.58 | 3.79 | 3.38 |
| Islamic Republic of Mauritania | 3.52 | 3.66 | 3.39 |
| Republic of Cameroon | 3.45 | 3.62 | 3.28 |
| Republic of Cabo Verde | 3.45 | 3.55 | 3.35 |
| Republic of Sierra Leone | 3.45 | 3.63 | 3.26 |
| Republic of Benin | 3.44 | 3.61 | 3.28 |
| Kyrgyz Republic | 3.37 | 4.63 | 2.12 |
| Republic of Guyana | 3.29 | 4.20 | 2.39 |
| Republic of Liberia | 3.17 | 3.55 | 2.80 |
| Republic of the Niger | 3.14 | 3.30 | 2.97 |
| Kingdom of Lesotho | 3.14 | 3.45 | 2.83 |
| Republic of Guinea-Bissau | 3.12 | 3.27 | 2.97 |
| Jamaica | 3.06 | 3.54 | 2.58 |
| Socialist Republic of Viet Nam | 3.04 | 3.09 | 3.00 |
| Republic of Latvia | 2.79 | 3.05 | 2.53 |
| Republic of Estonia | 2.73 | 3.03 | 2.42 |
| Republic of Bulgaria | 2.71 | 2.95 | 2.46 |
| Federal Republic of Nigeria | 2.44 | 2.64 | 2.24 |
| Dominican Republic | 2.41 | 2.58 | 2.25 |
| Islamic Republic of Iran | 2.35 | 2.48 | 2.21 |
| Republic of Zambia | 2.34 | 2.71 | 1.97 |
| Kingdom of Morocco | 2.28 | 2.44 | 2.12 |
| Republic of Lithuania | 2.25 | 2.41 | 2.09 |
| Mongolia | 2.19 | 2.37 | 2.01 |
| Republic of Honduras | 2.19 | 2.31 | 2.07 |
| Kingdom of Saudi Arabia | 2.17 | 2.25 | 2.10 |
| Republic of Kenya | 2.15 | 2.25 | 2.05 |
| Republic of Korea | 2.14 | 2.41 | 1.87 |
| Kingdom of Cambodia | 2.10 | 2.32 | 1.89 |
| Republic of Costa Rica | 2.10 | 2.31 | 1.90 |
| Taiwan (Province of China) | 2.08 | 2.42 | 1.75 |
| Kingdom of Eswatini | 2.08 | 2.47 | 1.69 |
| Republic of Moldova | 2.06 | 2.52 | 1.61 |
| Republic of Albania | 2.05 | 2.24 | 1.86 |
| Bosnia and Herzegovina | 2.02 | 2.17 | 1.86 |
| Grenada | 1.99 | 2.36 | 1.62 |
| Republic of the Union of Myanmar | 1.99 | 2.08 | 1.89 |
| Federal Democratic Republic of Nepal | 1.94 | 2.22 | 1.66 |
| Republic of Azerbaijan | 1.90 | 2.36 | 1.45 |
| Republic of Guinea | 1.89 | 1.95 | 1.82 |
| Republic of El Salvador | 1.87 | 1.99 | 1.76 |
| Republic of Iraq | 1.87 | 2.13 | 1.61 |
| Kingdom of Bhutan | 1.85 | 1.94 | 1.77 |
| Democratic Socialist Republic of Sri Lanka | 1.84 | 2.02 | 1.66 |
| Republic of India | 1.82 | 2.03 | 1.61 |
| Sultanate of Oman | 1.78 | 2.13 | 1.44 |
| Republic of Paraguay | 1.77 | 1.89 | 1.65 |
| Russian Federation | 1.75 | 2.08 | 1.42 |
| Republic of Angola | 1.72 | 1.89 | 1.55 |
| Lao People's Democratic Republic | 1.72 | 1.81 | 1.62 |
| Republic of the Gambia | 1.70 | 1.86 | 1.55 |
| Romania | 1.68 | 1.83 | 1.53 |
| Republic of the Philippines | 1.67 | 1.72 | 1.61 |
| Republic of Botswana | 1.66 | 1.86 | 1.45 |
| Republic of Mozambique | 1.65 | 1.75 | 1.55 |
| Republic of Indonesia | 1.64 | 1.69 | 1.60 |
| Republic of Poland | 1.62 | 2.05 | 1.19 |
| Ukraine | 1.60 | 1.85 | 1.35 |
| Republic of Croatia | 1.58 | 1.79 | 1.37 |
| Republic of Namibia | 1.58 | 1.70 | 1.45 |
| Republic of Uganda | 1.56 | 1.71 | 1.41 |
| Republic of South Africa | 1.56 | 1.69 | 1.43 |
| Republic of Nicaragua | 1.55 | 1.71 | 1.40 |
| Malaysia | 1.55 | 1.70 | 1.40 |
| United Arab Emirates | 1.54 | 1.98 | 1.09 |
| Republic of Tunisia | 1.52 | 1.58 | 1.46 |
| Syrian Arab Republic | 1.49 | 1.68 | 1.30 |
| Arab Republic of Egypt | 1.49 | 1.66 | 1.31 |
| Federative Republic of Brazil | 1.48 | 1.66 | 1.30 |
| Montenegro | 1.45 | 1.69 | 1.22 |
| Republic of Kazakhstan | 1.45 | 1.78 | 1.12 |
| Islamic Republic of Pakistan | 1.44 | 1.59 | 1.29 |
| Kingdom of Denmark | 1.41 | 1.82 | 1.01 |
| Republic of Zimbabwe | 1.40 | 1.84 | 0.95 |
| People's Democratic Republic of Algeria | 1.36 | 1.50 | 1.22 |
| Republic of Uzbekistan | 1.35 | 1.75 | 0.94 |
| Hellenic Republic | 1.31 | 1.45 | 1.17 |
| Cook Islands | 1.29 | 1.51 | 1.06 |
| Democratic Republic of Timor-Leste | 1.28 | 1.47 | 1.08 |
| North Macedonia | 1.27 | 1.46 | 1.09 |
| Principality of Monaco | 1.22 | 1.28 | 1.16 |
| Republic of Fiji | 1.21 | 1.35 | 1.08 |
| Republic of Sudan | 1.21 | 1.35 | 1.06 |
| Republic of Mali | 1.19 | 1.28 | 1.11 |
| Republic of Malawi | 1.19 | 1.26 | 1.13 |
| Republic of Suriname | 1.19 | 1.37 | 1.01 |
| Lebanese Republic | 1.18 | 1.38 | 0.97 |
| Islamic Republic of Afghanistan | 1.15 | 1.47 | 0.83 |
| Kingdom of Thailand | 1.13 | 1.36 | 0.89 |
| Australia | 1.13 | 1.28 | 0.98 |
| Republic of Colombia | 1.12 | 1.33 | 0.90 |
| Republic of Seychelles | 1.11 | 1.29 | 0.94 |
| Slovak Republic | 1.11 | 1.21 | 1.01 |
| State of Libya | 1.11 | 1.39 | 0.83 |
| United Mexican States | 1.10 | 1.23 | 0.97 |
| Republic of Turkey | 1.09 | 1.30 | 0.89 |
| Republic of Yemen | 1.09 | 1.24 | 0.93 |
| Republic of Guatemala | 1.08 | 1.20 | 0.96 |
| Federal Democratic Republic of Ethiopia | 1.08 | 1.46 | 0.70 |
| Republic of Peru | 1.07 | 1.37 | 0.77 |
| Republic of Slovenia | 1.05 | 1.28 | 0.82 |
| Antigua and Barbuda | 1.05 | 1.30 | 0.80 |
| Republic of Niue | 0.98 | 1.04 | 0.92 |
| Plurinational State of Bolivia | 0.97 | 1.06 | 0.88 |
| Republic of Cote d'Ivoire | 0.96 | 1.02 | 0.91 |
| Palestine | 0.96 | 1.01 | 0.91 |
| People's Republic of Bangladesh | 0.96 | 1.07 | 0.84 |
| Tuvalu | 0.95 | 1.07 | 0.83 |
| Tokelau | 0.94 | 0.97 | 0.91 |
| Saint Vincent and the Grenadines | 0.92 | 1.11 | 0.73 |
| Republic of Cuba | 0.90 | 1.00 | 0.81 |
| Republic of Serbia | 0.90 | 1.05 | 0.74 |
| Belize | 0.89 | 1.23 | 0.55 |
| Republic of Cyprus | 0.86 | 1.12 | 0.60 |
| Republic of Italy | 0.85 | 1.16 | 0.55 |
| Federal Republic of Germany | 0.85 | 1.21 | 0.50 |
| Republic of Panama | 0.85 | 1.03 | 0.67 |
| Barbados | 0.82 | 0.96 | 0.69 |
| Gabonese Republic | 0.78 | 0.86 | 0.71 |
| Republic of Trinidad and Tobago | 0.78 | 0.92 | 0.65 |
| Bolivarian Republic of Venezuela | 0.78 | 0.99 | 0.56 |
| Republic of Maldives | 0.74 | 0.86 | 0.62 |
| Saint Kitts and Nevis | 0.71 | 0.97 | 0.45 |
| Republic of Djibouti | 0.71 | 0.77 | 0.66 |
| United Kingdom of Great Britain and Northern Ireland | 0.71 | 0.86 | 0.56 |
| Republic of the Congo | 0.70 | 0.86 | 0.53 |
| French Republic | 0.69 | 0.94 | 0.45 |
| Republic of the Marshall Islands | 0.69 | 0.74 | 0.63 |
| American Samoa | 0.68 | 0.94 | 0.42 |
| Republic of Chile | 0.66 | 0.92 | 0.40 |
| Kingdom of Tonga | 0.66 | 0.83 | 0.49 |
| Kingdom of Bahrain | 0.66 | 0.82 | 0.49 |
| Republic of Austria | 0.64 | 0.88 | 0.40 |
| New Zealand | 0.64 | 0.83 | 0.45 |
| Republic of Iceland | 0.63 | 0.87 | 0.38 |
| Puerto Rico | 0.62 | 0.84 | 0.41 |
| Solomon Islands | 0.62 | 0.75 | 0.49 |
| Grand Duchy of Luxembourg | 0.61 | 0.86 | 0.35 |
| United Republic of Tanzania | 0.59 | 0.70 | 0.48 |
| Commonwealth of the Bahamas | 0.58 | 0.68 | 0.48 |
| Republic of Malta | 0.58 | 0.76 | 0.40 |
| Democratic People's Republic of Korea | 0.57 | 0.68 | 0.46 |
| Eastern Republic of Uruguay | 0.57 | 0.68 | 0.46 |
| Kingdom of the Netherlands | 0.55 | 0.77 | 0.33 |
| State of Eritrea | 0.52 | 0.72 | 0.32 |
| Republic of Finland | 0.52 | 0.68 | 0.36 |
| Republic of Vanuatu | 0.51 | 0.60 | 0.42 |
| State of Qatar | 0.51 | 0.87 | 0.14 |
| State of Israel | 0.50 | 0.72 | 0.27 |
| Commonwealth of Dominica | 0.49 | 0.60 | 0.38 |
| Czech Republic | 0.48 | 0.69 | 0.27 |
| Republic of Haiti | 0.46 | 0.51 | 0.41 |
| Independent State of Papua New Guinea | 0.45 | 0.57 | 0.34 |
| Kingdom of Norway | 0.45 | 0.68 | 0.22 |
| Independent State of Samoa | 0.44 | 0.49 | 0.39 |
| Portuguese Republic | 0.44 | 0.66 | 0.22 |
| Principality of Andorra | 0.43 | 0.66 | 0.20 |
| Federated States of Micronesia | 0.42 | 0.49 | 0.35 |
| Republic of Palau | 0.34 | 0.40 | 0.29 |
| Saint Lucia | 0.34 | 0.55 | 0.12 |
| Brunei Darussalam | 0.33 | 0.51 | 0.15 |
| Union of the Comoros | 0.30 | 0.40 | 0.20 |
| Hungary | 0.28 | 0.56 | 0.00 |
| Bermuda | 0.28 | 0.56 | 0.00 |
| Kingdom of Belgium | 0.25 | 0.41 | 0.08 |
| Republic of Kiribati | 0.23 | 0.31 | 0.16 |
| Ireland | 0.19 | 0.37 | 0.01 |
| Kingdom of Spain | 0.19 | 0.39 | -0.01 |
| Democratic Republic of the Congo | 0.18 | 0.59 | -0.23 |
| State of Kuwait | 0.16 | 1.04 | -0.71 |
| United States Virgin Islands | 0.09 | 0.36 | -0.18 |
| Swiss Confederation | 0.07 | 0.52 | -0.38 |
| Central African Republic | -0.01 | 0.05 | -0.07 |
| Hashemite Kingdom of Jordan | -0.03 | 0.19 | -0.24 |
| Guam | -0.05 | 0.23 | -0.34 |
| Republic of South Sudan | -0.05 | 0.01 | -0.12 |
| Republic of San Marino | -0.07 | 0.39 | -0.53 |
| Argentine Republic | -0.12 | 0.05 | -0.29 |
| Canada | -0.12 | 0.04 | -0.28 |
| Kingdom of Sweden | -0.17 | 0.01 | -0.35 |
| Greenland | -0.17 | -0.02 | -0.33 |
| Japan | -0.21 | -0.02 | -0.41 |
| Republic of Nauru | -0.26 | 0.06 | -0.57 |
| Republic of Madagascar | -0.30 | -0.09 | -0.51 |
| Republic of Rwanda | -0.34 | -0.06 | -0.62 |
| Federal Republic of Somalia | -0.45 | -0.41 | -0.50 |
| United States of America | -0.46 | -0.31 | -0.62 |
| Republic of Singapore | -0.54 | -0.39 | -0.69 |
| Republic of Tajikistan | -0.66 | -0.38 | -0.93 |
| Republic of Burundi | -1.19 | -0.98 | -1.40 |
| Northern Mariana Islands | -1.35 | -1.08 | -1.61 |

Supplementary Table S3. Age-standardized mortality rates of multiple myeloma for both sex by Country, 2021.

| location | val | upper | lower |
| --- | --- | --- | --- |
| Commonwealth of the Bahamas | 4.71 | 5.70 | 3.86 |
| Principality of Monaco | 4.40 | 6.84 | 2.29 |
| Grenada | 3.57 | 4.04 | 3.10 |
| Republic of Zimbabwe | 3.53 | 4.97 | 1.96 |
| Jamaica | 3.50 | 4.48 | 2.70 |
| Kingdom of Norway | 3.42 | 3.69 | 3.04 |
| Saint Lucia | 3.34 | 3.97 | 2.76 |
| New Zealand | 3.15 | 3.47 | 2.73 |
| Kingdom of the Netherlands | 3.15 | 3.43 | 2.78 |
| Republic of Uganda | 3.11 | 4.65 | 2.17 |
| Eastern Republic of Uruguay | 2.94 | 3.21 | 2.67 |
| United Kingdom of Great Britain and Northern Ireland | 2.89 | 3.02 | 2.64 |
| Antigua and Barbuda | 2.87 | 3.11 | 2.63 |
| State of Israel | 2.87 | 3.15 | 2.51 |
| United States of America | 2.84 | 2.99 | 2.58 |
| Australia | 2.84 | 3.20 | 2.46 |
| Republic of Iceland | 2.79 | 3.13 | 2.39 |
| Saint Kitts and Nevis | 2.77 | 3.27 | 2.27 |
| Kingdom of Denmark | 2.77 | 3.08 | 2.49 |
| French Republic | 2.75 | 3.11 | 2.35 |
| Republic of Trinidad and Tobago | 2.73 | 3.49 | 2.07 |
| Republic of Estonia | 2.72 | 3.10 | 2.33 |
| United Arab Emirates | 2.69 | 3.59 | 1.93 |
| Kingdom of Belgium | 2.69 | 2.96 | 2.35 |
| Ireland | 2.65 | 2.97 | 2.31 |
| Kingdom of Sweden | 2.64 | 2.97 | 2.29 |
| Canada | 2.60 | 2.85 | 2.31 |
| Republic of Italy | 2.59 | 2.78 | 2.31 |
| Republic of Slovenia | 2.58 | 2.99 | 2.22 |
| Puerto Rico | 2.54 | 2.97 | 2.12 |
| Lebanese Republic | 2.53 | 3.46 | 1.71 |
| Commonwealth of Dominica | 2.53 | 3.46 | 1.73 |
| Republic of Lithuania | 2.52 | 2.84 | 2.20 |
| Grand Duchy of Luxembourg | 2.51 | 2.77 | 2.23 |
| Republic of Finland | 2.49 | 2.76 | 2.20 |
| Republic of Poland | 2.49 | 2.72 | 2.25 |
| Bermuda | 2.49 | 3.11 | 2.09 |
| Republic of Costa Rica | 2.47 | 2.77 | 2.16 |
| Hellenic Republic | 2.46 | 2.62 | 2.25 |
| Portuguese Republic | 2.45 | 2.71 | 2.15 |
| Republic of Latvia | 2.44 | 2.86 | 2.07 |
| Republic of Cyprus | 2.42 | 3.24 | 1.55 |
| Swiss Confederation | 2.41 | 2.71 | 2.10 |
| Federal Republic of Germany | 2.40 | 2.60 | 2.14 |
| Kingdom of Eswatini | 2.37 | 3.52 | 1.60 |
| Slovak Republic | 2.34 | 3.12 | 1.57 |
| Republic of Zambia | 2.31 | 3.88 | 1.04 |
| Republic of Croatia | 2.30 | 2.70 | 1.96 |
| Republic of Chile | 2.29 | 2.50 | 2.09 |
| Czech Republic | 2.26 | 2.63 | 1.89 |
| Kingdom of Spain | 2.21 | 2.45 | 1.91 |
| United States Virgin Islands | 2.20 | 3.50 | 1.34 |
| Brunei Darussalam | 2.16 | 2.82 | 1.53 |
| Republic of Austria | 2.15 | 2.35 | 1.90 |
| Greenland | 2.09 | 3.09 | 1.57 |
| Republic of Cuba | 2.06 | 2.34 | 1.77 |
| Barbados | 2.06 | 2.61 | 1.56 |
| Republic of South Africa | 2.03 | 2.47 | 1.32 |
| State of Libya | 1.96 | 2.70 | 1.23 |
| Republic of Panama | 1.96 | 2.38 | 1.53 |
| Republic of Turkey | 1.89 | 2.62 | 1.39 |
| Republic of Botswana | 1.88 | 2.78 | 1.33 |
| Kingdom of Bahrain | 1.85 | 2.60 | 1.15 |
| Republic of Malta | 1.83 | 2.10 | 1.56 |
| Federative Republic of Brazil | 1.81 | 1.91 | 1.67 |
| Republic of Suriname | 1.77 | 2.44 | 1.22 |
| Saint Vincent and the Grenadines | 1.76 | 1.98 | 1.55 |
| Islamic Republic of Pakistan | 1.72 | 2.50 | 1.21 |
| Hungary | 1.72 | 1.98 | 1.48 |
| Kingdom of Tonga | 1.70 | 2.37 | 1.17 |
| Kingdom of Lesotho | 1.70 | 2.57 | 1.10 |
| Plurinational State of Bolivia | 1.69 | 2.53 | 1.16 |
| Republic of Mauritius | 1.67 | 1.77 | 1.54 |
| Republic of Belarus | 1.64 | 1.98 | 1.32 |
| Republic of Serbia | 1.63 | 2.14 | 1.15 |
| Argentine Republic | 1.61 | 1.73 | 1.48 |
| Republic of Djibouti | 1.59 | 2.41 | 0.92 |
| Principality of Andorra | 1.57 | 2.21 | 1.05 |
| Republic of Colombia | 1.55 | 1.87 | 1.28 |
| Palestine | 1.54 | 2.02 | 0.92 |
| Republic of Ecuador | 1.53 | 1.91 | 1.22 |
| Republic of San Marino | 1.53 | 2.31 | 0.96 |
| Dominican Republic | 1.53 | 2.09 | 1.07 |
| Republic of Seychelles | 1.51 | 1.98 | 1.13 |
| Republic of Peru | 1.46 | 2.09 | 1.02 |
| Bolivarian Republic of Venezuela | 1.45 | 1.90 | 1.08 |
| Republic of Tunisia | 1.39 | 1.97 | 0.86 |
| Republic of Namibia | 1.38 | 1.90 | 0.96 |
| Russian Federation | 1.37 | 1.48 | 1.26 |
| Republic of Rwanda | 1.36 | 2.02 | 0.81 |
| United Republic of Tanzania | 1.35 | 1.99 | 0.79 |
| Union of the Comoros | 1.34 | 1.98 | 0.73 |
| Romania | 1.32 | 1.49 | 1.14 |
| Republic of Kenya | 1.32 | 1.80 | 0.85 |
| Georgia | 1.32 | 1.53 | 1.14 |
| Japan | 1.30 | 1.40 | 1.12 |
| Taiwan (Province of China) | 1.29 | 1.42 | 1.16 |
| United Mexican States | 1.28 | 1.44 | 1.14 |
| Republic of Mozambique | 1.28 | 2.03 | 0.68 |
| Republic of Haiti | 1.27 | 2.36 | 0.71 |
| Republic of Cote d'Ivoire | 1.25 | 1.77 | 0.84 |
| Gabonese Republic | 1.24 | 1.78 | 0.71 |
| Republic of Paraguay | 1.20 | 1.73 | 0.79 |
| Republic of Iraq | 1.20 | 1.69 | 0.79 |
| Republic of Bulgaria | 1.19 | 1.54 | 0.92 |
| Sultanate of Oman | 1.19 | 1.61 | 0.80 |
| Republic of Honduras | 1.19 | 1.73 | 0.78 |
| State of Eritrea | 1.18 | 1.78 | 0.62 |
| Bosnia and Herzegovina | 1.18 | 1.59 | 0.80 |
| People's Democratic Republic of Algeria | 1.18 | 1.62 | 0.72 |
| Republic of Korea | 1.16 | 1.53 | 0.66 |
| Montenegro | 1.14 | 1.48 | 0.81 |
| Northern Mariana Islands | 1.13 | 1.64 | 0.87 |
| Republic of South Sudan | 1.13 | 1.76 | 0.60 |
| State of Qatar | 1.12 | 1.78 | 0.70 |
| American Samoa | 1.11 | 1.47 | 0.80 |
| Republic of Guyana | 1.08 | 1.38 | 0.84 |
| Kingdom of Bhutan | 1.07 | 1.93 | 0.56 |
| Kingdom of Saudi Arabia | 1.04 | 1.39 | 0.65 |
| Republic of India | 1.01 | 1.34 | 0.80 |
| North Macedonia | 1.01 | 1.32 | 0.66 |
| Republic of Equatorial Guinea | 0.99 | 1.54 | 0.57 |
| Turkmenistan | 0.99 | 1.34 | 0.74 |
| Democratic Socialist Republic of Sri Lanka | 0.97 | 1.44 | 0.61 |
| Hashemite Kingdom of Jordan | 0.97 | 1.42 | 0.62 |
| Ukraine | 0.92 | 1.19 | 0.66 |
| Malaysia | 0.87 | 1.29 | 0.69 |
| Republic of Madagascar | 0.86 | 1.27 | 0.45 |
| Republic of Fiji | 0.85 | 1.15 | 0.57 |
| Republic of Burundi | 0.84 | 1.60 | 0.40 |
| Republic of Armenia | 0.84 | 1.00 | 0.68 |
| Kingdom of Morocco | 0.82 | 1.29 | 0.51 |
| Republic of Moldova | 0.82 | 0.92 | 0.74 |
| Federal Democratic Republic of Nepal | 0.80 | 1.33 | 0.41 |
| State of Kuwait | 0.80 | 0.95 | 0.65 |
| Belize | 0.80 | 0.90 | 0.70 |
| Islamic Republic of Iran | 0.79 | 0.96 | 0.51 |
| Federal Republic of Nigeria | 0.78 | 1.19 | 0.30 |
| People's Republic of Bangladesh | 0.77 | 1.33 | 0.47 |
| Republic of Singapore | 0.73 | 0.81 | 0.64 |
| Republic of the Congo | 0.71 | 1.06 | 0.45 |
| Federal Democratic Republic of Ethiopia | 0.67 | 1.18 | 0.34 |
| Islamic Republic of Afghanistan | 0.64 | 1.34 | 0.27 |
| People's Republic of China | 0.62 | 0.81 | 0.40 |
| Republic of Malawi | 0.61 | 0.87 | 0.37 |
| Republic of Kazakhstan | 0.60 | 0.70 | 0.50 |
| Republic of Angola | 0.59 | 0.84 | 0.36 |
| Independent State of Samoa | 0.59 | 0.92 | 0.39 |
| Republic of Sudan | 0.58 | 0.97 | 0.35 |
| Republic of Nauru | 0.56 | 0.98 | 0.27 |
| Federal Republic of Somalia | 0.55 | 1.16 | 0.24 |
| Republic of El Salvador | 0.55 | 0.73 | 0.38 |
| Kingdom of Thailand | 0.55 | 1.04 | 0.34 |
| Republic of Maldives | 0.55 | 0.75 | 0.37 |
| Republic of Niue | 0.53 | 0.75 | 0.28 |
| Republic of Albania | 0.51 | 0.71 | 0.33 |
| Republic of Yemen | 0.48 | 0.86 | 0.26 |
| Republic of Guatemala | 0.47 | 0.55 | 0.40 |
| Guam | 0.47 | 0.64 | 0.35 |
| Syrian Arab Republic | 0.45 | 0.66 | 0.28 |
| Republic of Nicaragua | 0.44 | 0.58 | 0.31 |
| Kingdom of Cambodia | 0.44 | 0.92 | 0.25 |
| Republic of the Philippines | 0.42 | 0.58 | 0.32 |
| Republic of Indonesia | 0.42 | 0.79 | 0.26 |
| Tokelau | 0.40 | 0.58 | 0.19 |
| Lao People's Democratic Republic | 0.40 | 0.80 | 0.22 |
| Arab Republic of Egypt | 0.39 | 0.74 | 0.25 |
| Federated States of Micronesia | 0.38 | 0.59 | 0.19 |
| Central African Republic | 0.37 | 0.55 | 0.20 |
| Republic of the Union of Myanmar | 0.37 | 0.72 | 0.23 |
| Republic of the Marshall Islands | 0.35 | 0.59 | 0.17 |
| Tuvalu | 0.34 | 0.50 | 0.18 |
| Democratic Republic of the Congo | 0.34 | 0.57 | 0.12 |
| Republic of Vanuatu | 0.30 | 0.48 | 0.14 |
| Democratic People's Republic of Korea | 0.29 | 0.50 | 0.16 |
| Mongolia | 0.28 | 0.38 | 0.20 |
| Kyrgyz Republic | 0.28 | 0.35 | 0.22 |
| Socialist Republic of Viet Nam | 0.27 | 0.36 | 0.18 |
| Cook Islands | 0.26 | 0.35 | 0.18 |
| Democratic Republic of Timor-Leste | 0.26 | 0.51 | 0.16 |
| Solomon Islands | 0.25 | 0.46 | 0.12 |
| Republic of Azerbaijan | 0.24 | 0.36 | 0.15 |
| Republic of Guinea | 0.24 | 0.36 | 0.14 |
| Independent State of Papua New Guinea | 0.22 | 0.42 | 0.09 |
| Republic of Tajikistan | 0.21 | 0.30 | 0.14 |
| Republic of Uzbekistan | 0.19 | 0.24 | 0.15 |
| Republic of Cameroon | 0.13 | 0.22 | 0.04 |
| Republic of Cabo Verde | 0.13 | 0.22 | 0.05 |
| Republic of Ghana | 0.13 | 0.25 | 0.04 |
| Republic of the Gambia | 0.12 | 0.18 | 0.07 |
| Islamic Republic of Mauritania | 0.12 | 0.20 | 0.04 |
| Democratic Republic of Sao Tome and Principe | 0.11 | 0.18 | 0.03 |
| Republic of Guinea-Bissau | 0.11 | 0.16 | 0.04 |
| Republic of Senegal | 0.10 | 0.17 | 0.03 |
| Burkina Faso | 0.09 | 0.15 | 0.03 |
| Republic of Benin | 0.09 | 0.14 | 0.03 |
| Togolese Republic | 0.09 | 0.15 | 0.03 |
| Republic of Chad | 0.08 | 0.13 | 0.03 |
| Republic of Liberia | 0.08 | 0.15 | 0.02 |
| Republic of Sierra Leone | 0.07 | 0.13 | 0.02 |
| Republic of Kiribati | 0.07 | 0.10 | 0.04 |
| Republic of the Niger | 0.06 | 0.11 | 0.02 |
| Republic of Palau | 0.05 | 0.07 | 0.03 |
| Republic of Mali | 0.00 | 0.00 | 0.00 |

Supplementary Table S4. Estimated annual percentage change (EAPC) in age-standardized mortality rates of multiple myeloma for both sex by Country, 1990-2021.

| location | val | upper | lower |
| --- | --- | --- | --- |
| Georgia | 6.18 | 6.98 | 5.39 |
| Turkmenistan | 5.92 | 6.55 | 5.30 |
| Republic of Ghana | 4.93 | 5.18 | 4.68 |
| Democratic Republic of Sao Tome and Principe | 4.52 | 4.73 | 4.31 |
| Republic of Chad | 4.43 | 4.70 | 4.17 |
| Republic of Armenia | 4.23 | 4.87 | 3.58 |
| Burkina Faso | 3.96 | 4.13 | 3.79 |
| Republic of Equatorial Guinea | 3.75 | 4.00 | 3.51 |
| Togolese Republic | 3.62 | 3.78 | 3.46 |
| Republic of Senegal | 3.62 | 3.81 | 3.43 |
| Republic of Ecuador | 3.43 | 3.75 | 3.12 |
| Republic of Benin | 3.40 | 3.57 | 3.22 |
| Republic of Cameroon | 3.40 | 3.57 | 3.22 |
| Republic of Sierra Leone | 3.39 | 3.58 | 3.21 |
| Islamic Republic of Mauritania | 3.39 | 3.53 | 3.25 |
| Republic of Mauritius | 3.39 | 4.32 | 2.47 |
| Republic of Cabo Verde | 3.19 | 3.31 | 3.07 |
| Republic of the Niger | 3.12 | 3.28 | 2.95 |
| People's Republic of China | 3.11 | 3.86 | 2.37 |
| Kingdom of Lesotho | 3.10 | 3.42 | 2.79 |
| Republic of Guinea-Bissau | 3.09 | 3.24 | 2.93 |
| Kyrgyz Republic | 3.07 | 4.37 | 1.79 |
| Republic of Liberia | 3.05 | 3.42 | 2.69 |
| Republic of Guyana | 2.95 | 3.82 | 2.09 |
| Republic of Belarus | 2.74 | 3.00 | 2.49 |
| Jamaica | 2.73 | 3.20 | 2.27 |
| Socialist Republic of Viet Nam | 2.60 | 2.66 | 2.55 |
| Republic of Bulgaria | 2.52 | 2.79 | 2.25 |
| Federal Republic of Nigeria | 2.29 | 2.47 | 2.11 |
| Republic of Latvia | 2.23 | 2.46 | 2.01 |
| Republic of Zambia | 2.18 | 2.52 | 1.84 |
| Republic of Kenya | 2.09 | 2.18 | 1.99 |
| Republic of Honduras | 2.04 | 2.16 | 1.92 |
| Dominican Republic | 2.02 | 2.17 | 1.88 |
| Kingdom of Morocco | 2.01 | 2.15 | 1.87 |
| Kingdom of Eswatini | 2.00 | 2.40 | 1.60 |
| Mongolia | 1.93 | 2.09 | 1.78 |
| Kingdom of Cambodia | 1.89 | 2.09 | 1.69 |
| Islamic Republic of Iran | 1.84 | 1.99 | 1.70 |
| Republic of Guinea | 1.81 | 1.88 | 1.75 |
| Republic of the Union of Myanmar | 1.80 | 1.88 | 1.72 |
| Federal Democratic Republic of Nepal | 1.77 | 2.04 | 1.49 |
| Republic of Azerbaijan | 1.76 | 2.20 | 1.32 |
| Republic of Lithuania | 1.76 | 1.90 | 1.61 |
| Republic of Albania | 1.71 | 1.88 | 1.53 |
| Bosnia and Herzegovina | 1.68 | 1.80 | 1.56 |
| Republic of Estonia | 1.68 | 1.99 | 1.36 |
| Kingdom of Bhutan | 1.65 | 1.72 | 1.58 |
| Republic of the Gambia | 1.62 | 1.78 | 1.47 |
| Republic of Angola | 1.62 | 1.77 | 1.46 |
| Republic of India | 1.61 | 1.80 | 1.42 |
| Republic of Mozambique | 1.59 | 1.69 | 1.49 |
| Grenada | 1.58 | 1.95 | 1.22 |
| Republic of the Philippines | 1.58 | 1.63 | 1.53 |
| Lao People's Democratic Republic | 1.57 | 1.65 | 1.49 |
| Taiwan (Province of China) | 1.57 | 1.92 | 1.22 |
| Republic of Botswana | 1.56 | 1.79 | 1.34 |
| Ukraine | 1.55 | 1.84 | 1.27 |
| Republic of Costa Rica | 1.55 | 1.74 | 1.35 |
| Republic of Poland | 1.53 | 1.91 | 1.15 |
| Republic of Moldova | 1.53 | 1.94 | 1.12 |
| Republic of Paraguay | 1.52 | 1.65 | 1.40 |
| Republic of Indonesia | 1.50 | 1.55 | 1.45 |
| United Arab Emirates | 1.50 | 1.98 | 1.02 |
| Republic of Uganda | 1.49 | 1.64 | 1.33 |
| Kingdom of Saudi Arabia | 1.49 | 1.57 | 1.40 |
| Republic of Iraq | 1.48 | 1.70 | 1.26 |
| Republic of Namibia | 1.44 | 1.57 | 1.30 |
| Republic of Zimbabwe | 1.41 | 1.84 | 0.98 |
| Romania | 1.40 | 1.54 | 1.25 |
| Republic of South Africa | 1.38 | 1.53 | 1.24 |
| Islamic Republic of Pakistan | 1.35 | 1.51 | 1.18 |
| Russian Federation | 1.34 | 1.60 | 1.08 |
| Republic of El Salvador | 1.34 | 1.44 | 1.24 |
| Montenegro | 1.31 | 1.53 | 1.09 |
| Sultanate of Oman | 1.30 | 1.66 | 0.94 |
| Democratic Socialist Republic of Sri Lanka | 1.30 | 1.49 | 1.11 |
| Federative Republic of Brazil | 1.23 | 1.39 | 1.07 |
| Malaysia | 1.20 | 1.37 | 1.03 |
| Republic of Kazakhstan | 1.19 | 1.49 | 0.89 |
| Republic of Uzbekistan | 1.18 | 1.57 | 0.80 |
| Arab Republic of Egypt | 1.17 | 1.36 | 0.99 |
| Republic of Fiji | 1.15 | 1.27 | 1.02 |
| Republic of Nicaragua | 1.14 | 1.30 | 0.98 |
| Republic of Mali | 1.14 | 1.22 | 1.05 |
| Democratic Republic of Timor-Leste | 1.13 | 1.31 | 0.94 |
| Republic of Malawi | 1.10 | 1.17 | 1.03 |
| Islamic Republic of Afghanistan | 1.07 | 1.37 | 0.77 |
| Republic of Tunisia | 1.04 | 1.12 | 0.95 |
| North Macedonia | 1.04 | 1.23 | 0.84 |
| Syrian Arab Republic | 1.02 | 1.20 | 0.85 |
| People's Democratic Republic of Algeria | 1.02 | 1.16 | 0.87 |
| Republic of Sudan | 0.98 | 1.11 | 0.84 |
| Federal Democratic Republic of Ethiopia | 0.97 | 1.33 | 0.61 |
| Republic of Croatia | 0.97 | 1.16 | 0.78 |
| Hellenic Republic | 0.94 | 1.06 | 0.83 |
| Republic of Yemen | 0.94 | 1.08 | 0.79 |
| Republic of Suriname | 0.89 | 1.06 | 0.72 |
| Republic of Seychelles | 0.89 | 1.07 | 0.71 |
| Republic of Cote d'Ivoire | 0.87 | 0.93 | 0.82 |
| Republic of Korea | 0.83 | 1.01 | 0.66 |
| Republic of Niue | 0.83 | 0.90 | 0.76 |
| Tuvalu | 0.82 | 0.94 | 0.70 |
| Cook Islands | 0.82 | 1.03 | 0.61 |
| State of Libya | 0.78 | 1.04 | 0.53 |
| Principality of Monaco | 0.76 | 0.79 | 0.74 |
| United Mexican States | 0.74 | 0.84 | 0.63 |
| Plurinational State of Bolivia | 0.73 | 0.82 | 0.64 |
| People's Republic of Bangladesh | 0.71 | 0.82 | 0.59 |
| Tokelau | 0.71 | 0.75 | 0.67 |
| Republic of Guatemala | 0.70 | 0.83 | 0.57 |
| Republic of the Marshall Islands | 0.64 | 0.71 | 0.57 |
| Slovak Republic | 0.64 | 0.75 | 0.53 |
| Republic of Djibouti | 0.64 | 0.69 | 0.59 |
| Gabonese Republic | 0.64 | 0.70 | 0.57 |
| Palestine | 0.61 | 0.68 | 0.53 |
| Saint Vincent and the Grenadines | 0.60 | 0.79 | 0.41 |
| American Samoa | 0.59 | 0.86 | 0.33 |
| Republic of the Congo | 0.59 | 0.74 | 0.44 |
| Republic of Serbia | 0.58 | 0.71 | 0.45 |
| Antigua and Barbuda | 0.57 | 0.83 | 0.32 |
| Lebanese Republic | 0.57 | 0.77 | 0.37 |
| Kingdom of Tonga | 0.57 | 0.76 | 0.38 |
| Republic of Colombia | 0.55 | 0.76 | 0.34 |
| Kingdom of Denmark | 0.55 | 0.90 | 0.19 |
| Kingdom of Thailand | 0.54 | 0.79 | 0.30 |
| Solomon Islands | 0.52 | 0.64 | 0.40 |
| United Republic of Tanzania | 0.52 | 0.62 | 0.42 |
| Belize | 0.50 | 0.88 | 0.12 |
| State of Eritrea | 0.50 | 0.70 | 0.29 |
| Republic of Panama | 0.46 | 0.63 | 0.28 |
| Republic of Vanuatu | 0.46 | 0.54 | 0.38 |
| Republic of Slovenia | 0.45 | 0.66 | 0.25 |
| Bolivarian Republic of Venezuela | 0.44 | 0.63 | 0.24 |
| Republic of Turkey | 0.43 | 0.63 | 0.24 |
| Independent State of Papua New Guinea | 0.43 | 0.53 | 0.32 |
| Republic of Peru | 0.41 | 0.70 | 0.13 |
| Barbados | 0.34 | 0.44 | 0.24 |
| Commonwealth of Dominica | 0.32 | 0.41 | 0.23 |
| Independent State of Samoa | 0.30 | 0.36 | 0.25 |
| Republic of Haiti | 0.30 | 0.35 | 0.24 |
| Democratic People's Republic of Korea | 0.29 | 0.38 | 0.19 |
| Federated States of Micronesia | 0.28 | 0.37 | 0.20 |
| Portuguese Republic | 0.28 | 0.47 | 0.09 |
| Federal Republic of Germany | 0.28 | 0.48 | 0.08 |
| Republic of Cuba | 0.26 | 0.34 | 0.18 |
| Union of the Comoros | 0.25 | 0.34 | 0.15 |
| Republic of Palau | 0.24 | 0.30 | 0.18 |
| Republic of Italy | 0.23 | 0.39 | 0.07 |
| Eastern Republic of Uruguay | 0.22 | 0.32 | 0.12 |
| Republic of Kiribati | 0.21 | 0.29 | 0.14 |
| Republic of Trinidad and Tobago | 0.17 | 0.31 | 0.04 |
| Republic of Maldives | 0.17 | 0.30 | 0.04 |
| Commonwealth of the Bahamas | 0.16 | 0.21 | 0.12 |
| Republic of Austria | 0.16 | 0.31 | 0.01 |
| Australia | 0.14 | 0.26 | 0.01 |
| Brunei Darussalam | 0.12 | 0.32 | -0.07 |
| Democratic Republic of the Congo | 0.11 | 0.51 | -0.28 |
| Saint Kitts and Nevis | 0.10 | 0.34 | -0.13 |
| Kingdom of Bahrain | 0.10 | 0.31 | -0.11 |
| New Zealand | 0.09 | 0.25 | -0.07 |
| Republic of Iceland | 0.07 | 0.25 | -0.10 |
| Hungary | 0.01 | 0.27 | -0.25 |
| United Kingdom of Great Britain and Northern Ireland | -0.01 | 0.09 | -0.11 |
| Central African Republic | -0.02 | 0.04 | -0.08 |
| Republic of Chile | -0.02 | 0.24 | -0.28 |
| French Republic | -0.09 | 0.14 | -0.31 |
| Republic of South Sudan | -0.10 | -0.05 | -0.16 |
| Czech Republic | -0.14 | 0.02 | -0.30 |
| Grand Duchy of Luxembourg | -0.17 | 0.02 | -0.37 |
| Principality of Andorra | -0.19 | 0.02 | -0.40 |
| Republic of Malta | -0.19 | -0.03 | -0.35 |
| Saint Lucia | -0.20 | 0.00 | -0.39 |
| Republic of Finland | -0.21 | -0.06 | -0.36 |
| State of Israel | -0.22 | -0.02 | -0.42 |
| Kingdom of Norway | -0.25 | -0.17 | -0.34 |
| Kingdom of Spain | -0.27 | -0.07 | -0.47 |
| Republic of Cyprus | -0.30 | -0.12 | -0.47 |
| State of Qatar | -0.34 | 0.08 | -0.76 |
| Puerto Rico | -0.35 | -0.17 | -0.52 |
| Republic of Madagascar | -0.35 | -0.14 | -0.55 |
| Kingdom of Belgium | -0.37 | -0.25 | -0.48 |
| United States Virgin Islands | -0.37 | -0.11 | -0.63 |
| Republic of Nauru | -0.39 | -0.10 | -0.68 |
| Guam | -0.39 | -0.09 | -0.69 |
| Swiss Confederation | -0.41 | -0.05 | -0.76 |
| Republic of Rwanda | -0.43 | -0.16 | -0.70 |
| State of Kuwait | -0.44 | -0.45 | -1.32 |
| Federal Republic of Somalia | -0.44 | -0.40 | -0.48 |
| Argentine Republic | -0.46 | -0.30 | -0.62 |
| Canada | -0.58 | -0.48 | -0.68 |
| Kingdom of Sweden | -0.59 | -0.50 | -0.68 |
| Republic of San Marino | -0.59 | -0.16 | -1.02 |
| Hashemite Kingdom of Jordan | -0.62 | -0.38 | -0.86 |
| Kingdom of the Netherlands | -0.62 | -0.50 | -0.74 |
| Greenland | -0.63 | -0.48 | -0.79 |
| United States of America | -0.68 | -0.56 | -0.80 |
| Republic of Tajikistan | -0.70 | -0.44 | -0.97 |
| Bermuda | -0.73 | -0.53 | -0.93 |
| Ireland | -0.73 | -0.56 | -0.90 |
| Japan | -0.92 | -0.79 | -1.06 |
| Republic of Burundi | -1.20 | -1.00 | -1.41 |
| Republic of Singapore | -1.44 | -1.33 | -1.56 |
| Northern Mariana Islands | -1.45 | -1.20 | -1.70 |

Supplementary Table S5. Age-standardized disability-adjusted life years(DALYs) rates of multiple myeloma for both sex by Country, 2021.

| location | val | upper | lower |
| --- | --- | --- | --- |
| Commonwealth of the Bahamas | 117.52 | 145.13 | 94.29 |
| Principality of Monaco | 93.34 | 150.07 | 47.42 |
| Republic of Zimbabwe | 88.68 | 126.74 | 48.75 |
| Grenada | 84.44 | 96.69 | 73.31 |
| Jamaica | 83.97 | 108.33 | 63.83 |
| Saint Lucia | 80.73 | 97.66 | 66.34 |
| Republic of Uganda | 73.85 | 114.45 | 50.54 |
| Republic of Trinidad and Tobago | 68.79 | 89.07 | 51.55 |
| Kingdom of Norway | 68.12 | 72.59 | 62.22 |
| New Zealand | 67.57 | 73.84 | 60.03 |
| Republic of Estonia | 67.13 | 77.14 | 57.54 |
| Eastern Republic of Uruguay | 67.11 | 72.99 | 61.84 |
| Antigua and Barbuda | 66.61 | 72.52 | 61.13 |
| Republic of Lithuania | 65.57 | 74.52 | 57.19 |
| Kingdom of the Netherlands | 64.47 | 69.49 | 58.18 |
| Republic of Costa Rica | 63.78 | 71.80 | 55.89 |
| Republic of Latvia | 63.11 | 74.68 | 53.82 |
| Saint Kitts and Nevis | 63.04 | 75.96 | 50.97 |
| Kingdom of Eswatini | 62.53 | 96.05 | 40.69 |
| Puerto Rico | 62.31 | 73.67 | 51.81 |
| Commonwealth of Dominica | 60.28 | 83.74 | 41.81 |
| United Kingdom of Great Britain and Northern Ireland | 59.77 | 62.37 | 56.16 |
| Republic of Zambia | 59.44 | 104.61 | 24.57 |
| Australia | 59.30 | 66.69 | 52.35 |
| State of Israel | 58.31 | 63.56 | 52.47 |
| United States of America | 57.79 | 60.26 | 54.27 |
| United Arab Emirates | 57.68 | 77.14 | 41.04 |
| Bermuda | 57.64 | 72.25 | 47.97 |
| Republic of Iceland | 57.50 | 64.34 | 50.79 |
| Lebanese Republic | 57.04 | 78.48 | 40.04 |
| Republic of Slovenia | 55.68 | 64.92 | 47.22 |
| French Republic | 55.48 | 62.46 | 48.94 |
| United States Virgin Islands | 55.06 | 87.99 | 33.60 |
| Republic of Poland | 55.05 | 59.92 | 50.32 |
| Kingdom of Belgium | 54.91 | 60.29 | 49.32 |
| Kingdom of Denmark | 54.88 | 60.38 | 49.93 |
| Slovak Republic | 54.77 | 74.69 | 37.35 |
| Republic of Italy | 54.38 | 57.99 | 49.67 |
| Ireland | 54.29 | 60.89 | 48.24 |
| Canada | 52.98 | 57.47 | 48.39 |
| Republic of Chile | 52.51 | 57.04 | 48.53 |
| Republic of South Africa | 51.66 | 63.96 | 33.59 |
| Republic of Finland | 51.63 | 56.42 | 46.68 |
| Hellenic Republic | 51.60 | 54.76 | 47.75 |
| Republic of Croatia | 51.40 | 61.06 | 43.86 |
| Grand Duchy of Luxembourg | 51.18 | 56.47 | 45.39 |
| Czech Republic | 50.83 | 58.58 | 43.16 |
| Portuguese Republic | 50.81 | 55.92 | 45.82 |
| Federal Republic of Germany | 50.73 | 54.62 | 46.40 |
| Republic of Cuba | 50.61 | 57.70 | 43.84 |
| Kingdom of Sweden | 50.03 | 56.25 | 44.23 |
| Republic of Panama | 49.70 | 60.68 | 39.12 |
| Barbados | 48.88 | 62.08 | 36.55 |
| Swiss Confederation | 48.75 | 54.63 | 43.34 |
| Republic of Cyprus | 48.47 | 64.99 | 31.31 |
| Greenland | 48.31 | 70.67 | 36.76 |
| Brunei Darussalam | 48.01 | 62.54 | 34.64 |
| State of Libya | 47.29 | 66.92 | 29.98 |
| Republic of Botswana | 46.70 | 68.30 | 31.86 |
| Republic of Turkey | 45.96 | 62.25 | 33.70 |
| Kingdom of Spain | 45.93 | 50.17 | 40.90 |
| Republic of Suriname | 44.81 | 61.50 | 30.77 |
| Saint Vincent and the Grenadines | 44.04 | 50.33 | 38.44 |
| Republic of Austria | 43.81 | 47.79 | 39.55 |
| Federative Republic of Brazil | 43.77 | 45.58 | 41.32 |
| Kingdom of Lesotho | 43.74 | 67.44 | 27.53 |
| Republic of Belarus | 43.01 | 52.81 | 34.22 |
| Republic of Mauritius | 42.85 | 45.21 | 39.23 |
| Kingdom of Tonga | 41.61 | 59.20 | 28.27 |
| Kingdom of Bahrain | 41.25 | 57.90 | 24.46 |
| Islamic Republic of Pakistan | 40.86 | 58.75 | 28.80 |
| Plurinational State of Bolivia | 40.73 | 61.06 | 27.94 |
| Hungary | 40.10 | 46.58 | 34.21 |
| Republic of Colombia | 38.78 | 47.05 | 32.14 |
| Republic of Malta | 38.65 | 43.69 | 33.30 |
| Republic of Djibouti | 38.56 | 59.24 | 21.88 |
| Georgia | 38.46 | 44.33 | 33.11 |
| Dominican Republic | 38.32 | 53.27 | 26.81 |
| Argentine Republic | 37.91 | 40.71 | 35.14 |
| Republic of Serbia | 37.90 | 49.88 | 26.42 |
| Bolivarian Republic of Venezuela | 37.31 | 49.43 | 27.45 |
| Republic of Ecuador | 37.29 | 47.22 | 29.45 |
| Palestine | 37.18 | 49.08 | 21.86 |
| Republic of Seychelles | 37.12 | 48.52 | 27.71 |
| Republic of Peru | 35.86 | 51.60 | 24.96 |
| Russian Federation | 35.48 | 38.34 | 32.66 |
| Republic of Namibia | 34.25 | 48.48 | 23.08 |
| United Mexican States | 33.95 | 38.28 | 29.91 |
| Principality of Andorra | 33.47 | 47.60 | 21.97 |
| Republic of Rwanda | 32.82 | 49.15 | 18.91 |
| Taiwan (Province of China) | 32.80 | 35.68 | 29.82 |
| United Republic of Tanzania | 32.79 | 48.95 | 18.66 |
| Republic of San Marino | 32.55 | 49.54 | 19.89 |
| Romania | 32.45 | 36.68 | 27.95 |
| Union of the Comoros | 32.41 | 47.83 | 17.30 |
| Republic of Tunisia | 32.34 | 47.24 | 20.41 |
| Turkmenistan | 31.83 | 43.57 | 23.83 |
| Republic of Kenya | 31.74 | 43.87 | 20.45 |
| Republic of Mozambique | 31.53 | 50.41 | 16.22 |
| Republic of Haiti | 31.03 | 58.78 | 17.51 |
| Gabonese Republic | 30.90 | 45.17 | 17.37 |
| Republic of Honduras | 29.69 | 44.03 | 19.34 |
| Republic of Bulgaria | 29.66 | 38.34 | 22.91 |
| Republic of Iraq | 29.64 | 42.24 | 19.40 |
| Republic of Cote d'Ivoire | 29.53 | 42.05 | 19.16 |
| State of Eritrea | 29.33 | 44.34 | 15.38 |
| Republic of Paraguay | 29.06 | 41.36 | 19.31 |
| Sultanate of Oman | 27.97 | 39.14 | 18.69 |
| Republic of Guyana | 27.90 | 36.26 | 21.29 |
| Republic of South Sudan | 27.66 | 43.92 | 14.22 |
| American Samoa | 27.53 | 37.13 | 20.16 |
| Bosnia and Herzegovina | 27.36 | 36.36 | 18.46 |
| Northern Mariana Islands | 27.14 | 38.89 | 20.94 |
| Ukraine | 26.40 | 34.28 | 18.91 |
| People's Democratic Republic of Algeria | 25.79 | 34.87 | 16.29 |
| Japan | 25.68 | 27.30 | 23.20 |
| Kingdom of Saudi Arabia | 25.68 | 34.33 | 16.38 |
| Montenegro | 25.55 | 33.98 | 18.00 |
| State of Qatar | 25.32 | 40.27 | 15.71 |
| Republic of Korea | 24.74 | 32.59 | 14.64 |
| Kingdom of Bhutan | 24.58 | 44.49 | 12.43 |
| Republic of Equatorial Guinea | 24.40 | 38.96 | 13.69 |
| Republic of India | 24.22 | 32.16 | 19.08 |
| Democratic Socialist Republic of Sri Lanka | 23.71 | 35.20 | 14.31 |
| Hashemite Kingdom of Jordan | 23.48 | 34.26 | 14.68 |
| Republic of Armenia | 23.40 | 27.89 | 18.96 |
| North Macedonia | 22.23 | 29.33 | 14.42 |
| Republic of Moldova | 22.16 | 24.85 | 19.81 |
| Malaysia | 21.91 | 31.69 | 17.37 |
| Republic of Madagascar | 21.19 | 31.40 | 10.84 |
| Republic of Fiji | 20.73 | 28.91 | 13.49 |
| Kingdom of Morocco | 20.47 | 32.26 | 12.32 |
| Republic of Burundi | 20.40 | 39.41 | 9.50 |
| Belize | 20.13 | 22.97 | 17.60 |
| State of Kuwait | 19.40 | 23.06 | 15.88 |
| Islamic Republic of Iran | 19.38 | 23.03 | 12.38 |
| Federal Democratic Republic of Nepal | 18.77 | 31.49 | 9.54 |
| People's Republic of Bangladesh | 18.21 | 31.13 | 10.76 |
| Federal Republic of Nigeria | 17.84 | 27.90 | 6.39 |
| Republic of Kazakhstan | 17.74 | 20.90 | 14.84 |
| Republic of the Congo | 17.73 | 26.58 | 11.24 |
| Islamic Republic of Afghanistan | 16.66 | 35.61 | 6.86 |
| People's Republic of China | 16.12 | 21.35 | 10.09 |
| Federal Democratic Republic of Ethiopia | 15.64 | 27.81 | 7.91 |
| Republic of Singapore | 15.13 | 16.67 | 13.62 |
| Republic of Sudan | 14.81 | 24.38 | 8.70 |
| Republic of El Salvador | 14.74 | 19.67 | 9.79 |
| Republic of Malawi | 14.68 | 20.93 | 8.89 |
| Republic of Angola | 14.62 | 21.25 | 8.96 |
| Independent State of Samoa | 14.44 | 22.98 | 9.71 |
| Republic of Nauru | 14.36 | 26.70 | 6.47 |
| Kingdom of Thailand | 14.06 | 26.55 | 8.76 |
| Federal Republic of Somalia | 13.63 | 29.17 | 5.69 |
| Guam | 13.36 | 17.61 | 9.96 |
| Republic of Niue | 13.25 | 18.95 | 6.82 |
| Republic of Maldives | 12.64 | 16.96 | 8.98 |
| Republic of Guatemala | 12.61 | 14.78 | 10.75 |
| Republic of Yemen | 11.96 | 21.14 | 6.41 |
| Republic of Nicaragua | 11.64 | 15.13 | 8.14 |
| Kingdom of Cambodia | 11.09 | 22.84 | 6.24 |
| Syrian Arab Republic | 11.08 | 16.38 | 6.78 |
| Republic of the Philippines | 11.07 | 14.66 | 8.24 |
| Republic of Albania | 11.07 | 15.35 | 7.24 |
| Republic of Indonesia | 10.37 | 19.35 | 6.70 |
| Tokelau | 10.14 | 15.01 | 4.99 |
| Lao People's Democratic Republic | 10.11 | 19.95 | 5.42 |
| Federated States of Micronesia | 9.72 | 15.69 | 4.85 |
| Central African Republic | 9.64 | 14.66 | 5.22 |
| Arab Republic of Egypt | 9.55 | 17.39 | 6.16 |
| Republic of the Union of Myanmar | 9.33 | 18.00 | 5.70 |
| Republic of the Marshall Islands | 8.94 | 15.57 | 4.34 |
| Tuvalu | 8.70 | 12.94 | 4.61 |
| Kyrgyz Republic | 8.48 | 10.60 | 6.55 |
| Democratic Republic of the Congo | 8.47 | 14.34 | 3.09 |
| Mongolia | 8.40 | 11.50 | 5.91 |
| Democratic People's Republic of Korea | 8.04 | 13.74 | 4.58 |
| Republic of Vanuatu | 7.55 | 12.06 | 3.66 |
| Republic of Azerbaijan | 7.12 | 10.61 | 4.47 |
| Solomon Islands | 6.60 | 11.82 | 3.10 |
| Socialist Republic of Viet Nam | 6.52 | 8.85 | 4.42 |
| Democratic Republic of Timor-Leste | 6.39 | 12.63 | 3.84 |
| Cook Islands | 6.36 | 8.66 | 4.38 |
| Republic of Tajikistan | 6.11 | 8.88 | 4.06 |
| Republic of Uzbekistan | 5.81 | 7.42 | 4.40 |
| Republic of Guinea | 5.67 | 8.71 | 3.12 |
| Independent State of Papua New Guinea | 5.54 | 10.22 | 2.18 |
| Republic of Cameroon | 3.12 | 5.14 | 0.97 |
| Republic of Ghana | 3.04 | 5.98 | 0.87 |
| Republic of Cabo Verde | 3.02 | 5.08 | 1.11 |
| Republic of the Gambia | 2.93 | 4.31 | 1.75 |
| Islamic Republic of Mauritania | 2.76 | 4.68 | 0.85 |
| Republic of Guinea-Bissau | 2.58 | 4.04 | 0.91 |
| Democratic Republic of Sao Tome and Principe | 2.53 | 4.11 | 0.79 |
| Republic of Senegal | 2.40 | 4.07 | 0.78 |
| Burkina Faso | 2.13 | 3.58 | 0.72 |
| Togolese Republic | 2.10 | 3.65 | 0.60 |
| Republic of Benin | 2.08 | 3.40 | 0.64 |
| Republic of Chad | 1.85 | 3.10 | 0.64 |
| Republic of Liberia | 1.82 | 3.69 | 0.52 |
| Republic of Kiribati | 1.76 | 2.57 | 0.96 |
| Republic of Sierra Leone | 1.71 | 3.01 | 0.51 |
| Republic of the Niger | 1.37 | 2.63 | 0.41 |
| Republic of Palau | 1.21 | 1.77 | 0.70 |
| Republic of Mali | 0.00 | 0.00 | 0.00 |

Supplementary Table S6. Estimated annual percentage change (EAPC) in disability-adjusted life years(DALYs) rates of multiple myeloma for both sex by Country, 1990-2021.

| location | val | upper | lower |
| --- | --- | --- | --- |
| Turkmenistan | 6.04 | 6.66 | 5.42 |
| Georgia | 5.98 | 6.77 | 5.20 |
| Republic of Ghana | 4.75 | 4.99 | 4.51 |
| Republic of Chad | 4.35 | 4.62 | 4.09 |
| Democratic Republic of Sao Tome and Principe | 4.33 | 4.54 | 4.12 |
| Republic of Armenia | 4.14 | 4.80 | 3.48 |
| Burkina Faso | 3.84 | 4.00 | 3.69 |
| Republic of Equatorial Guinea | 3.57 | 3.79 | 3.34 |
| Togolese Republic | 3.54 | 3.70 | 3.38 |
| Republic of Senegal | 3.51 | 3.70 | 3.31 |
| Republic of Mauritius | 3.41 | 4.33 | 2.49 |
| Republic of Sierra Leone | 3.36 | 3.56 | 3.17 |
| Republic of Ecuador | 3.32 | 3.63 | 3.01 |
| Republic of Cameroon | 3.30 | 3.47 | 3.13 |
| Kyrgyz Republic | 3.28 | 4.37 | 2.21 |
| Republic of Benin | 3.26 | 3.43 | 3.10 |
| Kingdom of Lesotho | 3.21 | 3.55 | 2.86 |
| Islamic Republic of Mauritania | 3.18 | 3.32 | 3.05 |
| People's Republic of China | 3.15 | 3.87 | 2.44 |
| Republic of Cabo Verde | 3.11 | 3.23 | 3.00 |
| Republic of Liberia | 3.01 | 3.38 | 2.64 |
| Republic of Guinea-Bissau | 2.99 | 3.15 | 2.83 |
| Republic of Guyana | 2.98 | 3.82 | 2.14 |
| Republic of the Niger | 2.96 | 3.11 | 2.80 |
| Republic of Belarus | 2.74 | 3.03 | 2.45 |
| Jamaica | 2.64 | 3.11 | 2.16 |
| Socialist Republic of Viet Nam | 2.59 | 2.64 | 2.54 |
| Republic of Bulgaria | 2.47 | 2.72 | 2.22 |
| Federal Republic of Nigeria | 2.35 | 2.54 | 2.15 |
| Republic of Zambia | 2.32 | 2.68 | 1.96 |
| Republic of Latvia | 2.24 | 2.49 | 2.00 |
| Republic of Kenya | 2.11 | 2.20 | 2.01 |
| Dominican Republic | 2.09 | 2.20 | 1.98 |
| Kingdom of Eswatini | 2.08 | 2.53 | 1.64 |
| Mongolia | 2.01 | 2.16 | 1.85 |
| Kingdom of Morocco | 1.96 | 2.08 | 1.83 |
| Republic of Honduras | 1.88 | 1.99 | 1.77 |
| Republic of Guinea | 1.85 | 1.94 | 1.77 |
| Republic of Costa Rica | 1.81 | 2.01 | 1.60 |
| Kingdom of Cambodia | 1.80 | 2.00 | 1.61 |
| Republic of Lithuania | 1.80 | 1.99 | 1.61 |
| Islamic Republic of Iran | 1.75 | 1.89 | 1.60 |
| Republic of the Union of Myanmar | 1.69 | 1.76 | 1.62 |
| Federal Democratic Republic of Nepal | 1.66 | 1.95 | 1.38 |
| Republic of Mozambique | 1.64 | 1.75 | 1.54 |
| Republic of Albania | 1.64 | 1.82 | 1.46 |
| Bosnia and Herzegovina | 1.63 | 1.78 | 1.49 |
| Kingdom of Saudi Arabia | 1.63 | 1.73 | 1.53 |
| Republic of Zimbabwe | 1.62 | 2.09 | 1.14 |
| Republic of the Philippines | 1.59 | 1.63 | 1.56 |
| Republic of the Gambia | 1.59 | 1.77 | 1.41 |
| Republic of Angola | 1.55 | 1.71 | 1.38 |
| Republic of Moldova | 1.52 | 1.92 | 1.12 |
| Republic of Azerbaijan | 1.49 | 1.93 | 1.05 |
| Grenada | 1.49 | 1.78 | 1.20 |
| Republic of Estonia | 1.48 | 1.86 | 1.11 |
| Kingdom of Bhutan | 1.48 | 1.55 | 1.40 |
| Republic of Paraguay | 1.47 | 1.58 | 1.36 |
| Lao People's Democratic Republic | 1.46 | 1.53 | 1.39 |
| Republic of India | 1.46 | 1.65 | 1.27 |
| Taiwan (Province of China) | 1.44 | 1.74 | 1.13 |
| Republic of Botswana | 1.42 | 1.66 | 1.18 |
| Ukraine | 1.41 | 1.68 | 1.15 |
| Republic of Uganda | 1.39 | 1.57 | 1.21 |
| Republic of El Salvador | 1.38 | 1.49 | 1.28 |
| Republic of Indonesia | 1.38 | 1.43 | 1.33 |
| Republic of Namibia | 1.37 | 1.53 | 1.21 |
| Republic of South Africa | 1.34 | 1.48 | 1.21 |
| Islamic Republic of Pakistan | 1.32 | 1.49 | 1.15 |
| Republic of Iraq | 1.31 | 1.50 | 1.12 |
| Republic of Uzbekistan | 1.27 | 1.65 | 0.90 |
| Democratic Socialist Republic of Sri Lanka | 1.24 | 1.42 | 1.06 |
| Republic of Fiji | 1.21 | 1.37 | 1.04 |
| Arab Republic of Egypt | 1.18 | 1.36 | 1.00 |
| Republic of Nicaragua | 1.17 | 1.32 | 1.03 |
| Malaysia | 1.17 | 1.30 | 1.04 |
| Romania | 1.15 | 1.30 | 1.00 |
| Sultanate of Oman | 1.13 | 1.51 | 0.75 |
| Montenegro | 1.11 | 1.27 | 0.95 |
| Democratic Republic of Timor-Leste | 1.09 | 1.29 | 0.88 |
| Republic of Malawi | 1.08 | 1.16 | 1.01 |
| Republic of Poland | 1.07 | 1.44 | 0.69 |
| Republic of Mali | 1.06 | 1.14 | 0.98 |
| Republic of Tunisia | 1.05 | 1.13 | 0.97 |
| Islamic Republic of Afghanistan | 1.04 | 1.33 | 0.74 |
| Republic of Kazakhstan | 1.00 | 1.26 | 0.75 |
| Federative Republic of Brazil | 0.97 | 1.14 | 0.80 |
| Republic of Sudan | 0.95 | 1.08 | 0.81 |
| Russian Federation | 0.94 | 1.16 | 0.72 |
| Syrian Arab Republic | 0.91 | 1.07 | 0.76 |
| Republic of Suriname | 0.91 | 1.08 | 0.74 |
| People's Democratic Republic of Algeria | 0.90 | 1.00 | 0.79 |
| Cook Islands | 0.89 | 1.12 | 0.66 |
| Hellenic Republic | 0.87 | 0.95 | 0.78 |
| North Macedonia | 0.86 | 1.03 | 0.69 |
| Republic of Croatia | 0.85 | 1.06 | 0.64 |
| Republic of Guatemala | 0.85 | 0.98 | 0.71 |
| Republic of Yemen | 0.85 | 0.99 | 0.70 |
| Republic of Cote d'Ivoire | 0.83 | 0.91 | 0.76 |
| United Mexican States | 0.78 | 0.90 | 0.67 |
| Republic of Seychelles | 0.78 | 0.93 | 0.62 |
| Tuvalu | 0.76 | 0.87 | 0.65 |
| Republic of Niue | 0.75 | 0.82 | 0.68 |
| Federal Democratic Republic of Ethiopia | 0.75 | 1.12 | 0.39 |
| State of Libya | 0.74 | 0.99 | 0.49 |
| Tokelau | 0.73 | 0.77 | 0.70 |
| Saint Vincent and the Grenadines | 0.70 | 0.86 | 0.54 |
| Kingdom of Thailand | 0.67 | 0.91 | 0.42 |
| Solomon Islands | 0.66 | 0.79 | 0.53 |
| United Arab Emirates | 0.65 | 1.05 | 0.26 |
| American Samoa | 0.62 | 0.89 | 0.35 |
| Republic of the Marshall Islands | 0.62 | 0.69 | 0.55 |
| Republic of Serbia | 0.62 | 0.73 | 0.51 |
| Plurinational State of Bolivia | 0.62 | 0.71 | 0.52 |
| Principality of Monaco | 0.61 | 0.63 | 0.59 |
| People's Republic of Bangladesh | 0.61 | 0.71 | 0.50 |
| Palestine | 0.59 | 0.64 | 0.53 |
| Belize | 0.58 | 0.95 | 0.22 |
| Gabonese Republic | 0.56 | 0.63 | 0.49 |
| Republic of Djibouti | 0.56 | 0.61 | 0.51 |
| Republic of Korea | 0.55 | 0.76 | 0.34 |
| Republic of Vanuatu | 0.55 | 0.65 | 0.45 |
| Kingdom of Tonga | 0.54 | 0.71 | 0.37 |
| Republic of Panama | 0.53 | 0.71 | 0.36 |
| Slovak Republic | 0.50 | 0.60 | 0.39 |
| Republic of the Congo | 0.49 | 0.65 | 0.34 |
| Antigua and Barbuda | 0.48 | 0.71 | 0.25 |
| United Republic of Tanzania | 0.47 | 0.57 | 0.37 |
| Independent State of Papua New Guinea | 0.46 | 0.57 | 0.35 |
| Commonwealth of Dominica | 0.45 | 0.54 | 0.36 |
| Republic of Colombia | 0.45 | 0.65 | 0.24 |
| Lebanese Republic | 0.41 | 0.60 | 0.23 |
| Bolivarian Republic of Venezuela | 0.41 | 0.60 | 0.22 |
| Republic of Peru | 0.41 | 0.70 | 0.12 |
| Republic of Cuba | 0.35 | 0.46 | 0.25 |
| State of Eritrea | 0.35 | 0.55 | 0.14 |
| Independent State of Samoa | 0.34 | 0.38 | 0.30 |
| Barbados | 0.32 | 0.41 | 0.22 |
| Democratic People's Republic of Korea | 0.31 | 0.39 | 0.23 |
| Republic of Turkey | 0.30 | 0.49 | 0.11 |
| Federated States of Micronesia | 0.29 | 0.37 | 0.22 |
| Republic of Haiti | 0.26 | 0.31 | 0.20 |
| Republic of Trinidad and Tobago | 0.25 | 0.38 | 0.11 |
| Republic of Kiribati | 0.17 | 0.25 | 0.09 |
| Guam | 0.17 | 0.44 | -0.10 |
| Republic of Slovenia | 0.11 | 0.32 | -0.09 |
| Democratic Republic of the Congo | 0.11 | 0.50 | -0.29 |
| Union of the Comoros | 0.10 | 0.23 | -0.02 |
| Commonwealth of the Bahamas | 0.10 | 0.14 | 0.06 |
| Republic of Palau | 0.10 | 0.16 | 0.04 |
| Eastern Republic of Uruguay | 0.09 | 0.19 | -0.01 |
| Kingdom of Denmark | 0.05 | 0.39 | -0.30 |
| Federal Republic of Germany | 0.04 | 0.25 | -0.18 |
| Republic of Maldives | 0.04 | 0.17 | -0.09 |
| Kingdom of Bahrain | -0.02 | 0.14 | -0.18 |
| Saint Kitts and Nevis | -0.02 | 0.21 | -0.25 |
| Portuguese Republic | -0.04 | 0.16 | -0.23 |
| Central African Republic | -0.05 | 0.02 | -0.11 |
| Brunei Darussalam | -0.05 | 0.11 | -0.22 |
| Saint Lucia | -0.05 | 0.13 | -0.24 |
| Republic of Italy | -0.06 | 0.10 | -0.23 |
| Australia | -0.07 | 0.03 | -0.17 |
| New Zealand | -0.11 | 0.04 | -0.27 |
| Republic of Austria | -0.13 | 0.05 | -0.31 |
| Republic of South Sudan | -0.13 | -0.06 | -0.21 |
| Republic of Chile | -0.17 | 0.08 | -0.41 |
| Republic of Cyprus | -0.17 | 0.02 | -0.37 |
| Hungary | -0.18 | 0.08 | -0.43 |
| United States Virgin Islands | -0.19 | 0.05 | -0.42 |
| Republic of Malta | -0.19 | -0.03 | -0.34 |
| Republic of Iceland | -0.19 | -0.03 | -0.36 |
| Republic of Nauru | -0.23 | 0.03 | -0.49 |
| Principality of Andorra | -0.24 | -0.04 | -0.45 |
| State of Qatar | -0.27 | 0.10 | -0.63 |
| Puerto Rico | -0.27 | -0.10 | -0.44 |
| United Kingdom of Great Britain and Northern Ireland | -0.28 | -0.18 | -0.38 |
| French Republic | -0.30 | -0.07 | -0.53 |
| Czech Republic | -0.31 | -0.16 | -0.46 |
| State of Israel | -0.34 | -0.12 | -0.55 |
| Grand Duchy of Luxembourg | -0.37 | -0.17 | -0.58 |
| Republic of Madagascar | -0.39 | -0.18 | -0.59 |
| State of Kuwait | -0.46 | 0.34 | -1.26 |
| Republic of Finland | -0.46 | -0.32 | -0.61 |
| Kingdom of Belgium | -0.47 | -0.30 | -0.64 |
| Argentine Republic | -0.49 | -0.34 | -0.65 |
| Republic of San Marino | -0.51 | -0.10 | -0.93 |
| Kingdom of Norway | -0.53 | -0.41 | -0.66 |
| Federal Republic of Somalia | -0.54 | -0.50 | -0.59 |
| Kingdom of Spain | -0.64 | -0.45 | -0.84 |
| Republic of Rwanda | -0.66 | -0.37 | -0.95 |
| Hashemite Kingdom of Jordan | -0.68 | -0.44 | -0.92 |
| Greenland | -0.69 | -0.55 | -0.82 |
| Republic of Tajikistan | -0.75 | -0.46 | -1.04 |
| Bermuda | -0.76 | -0.57 | -0.96 |
| Swiss Confederation | -0.78 | -0.43 | -1.13 |
| Kingdom of the Netherlands | -0.79 | -0.67 | -0.90 |
| Kingdom of Sweden | -0.82 | -0.67 | -0.97 |
| Ireland | -0.93 | -0.75 | -1.11 |
| Canada | -0.94 | -0.83 | -1.05 |
| United States of America | -1.05 | -0.93 | -1.18 |
| Japan | -1.24 | -1.08 | -1.39 |
| Northern Mariana Islands | -1.32 | -1.05 | -1.59 |
| Republic of Burundi | -1.34 | -1.12 | -1.56 |
| Republic of Singapore | -1.74 | -1.62 | -1.86 |

Supplementary Table S7. Global age and sex structure of multiple myeloma incidence, mortality and disability-adjusted life years, 2021.

| sex | age | metric | incidence | | | mortality | | | disability-adjusted life years | | |
| --- | --- | --- | --- | --- | --- | --- | --- | --- | --- | --- | --- |
|  |  |  | val | upper | lower | val | upper | lower | val | upper | lower |
| Male | <5 years | Number | 0 | 0 | 0 | 0 | 0 | 0 | 0 | 0 | 0 |
| Female | <5 years | Number | 0 | 0 | 0 | 0 | 0 | 0 | 0 | 0 | 0 |
| Male | <5 years | Rate | 0 | 0 | 0 | 0 | 0 | 0 | 0 | 0 | 0 |
| Female | <5 years | Rate | 0 | 0 | 0 | 0 | 0 | 0 | 0 | 0 | 0 |
| Male | 5-9 years | Number | 0 | 0 | 0 | 0 | 0 | 0 | 0 | 0 | 0 |
| Female | 5-9 years | Number | 0 | 0 | 0 | 0 | 0 | 0 | 0 | 0 | 0 |
| Male | 5-9 years | Rate | 0 | 0 | 0 | 0 | 0 | 0 | 0 | 0 | 0 |
| Female | 5-9 years | Rate | 0 | 0 | 0 | 0 | 0 | 0 | 0 | 0 | 0 |
| Male | 10-14 years | Number | 0 | 0 | 0 | 0 | 0 | 0 | 0 | 0 | 0 |
| Female | 10-14 years | Number | 0 | 0 | 0 | 0 | 0 | 0 | 0 | 0 | 0 |
| Male | 10-14 years | Rate | 0 | 0 | 0 | 0 | 0 | 0 | 0 | 0 | 0 |
| Female | 10-14 years | Rate | 0 | 0 | 0 | 0 | 0 | 0 | 0 | 0 | 0 |
| Male | 15-19 years | Number | 0 | 0 | 0 | 0 | 0 | 0 | 0 | 0 | 0 |
| Female | 15-19 years | Number | 0 | 0 | 0 | 0 | 0 | 0 | 0 | 0 | 0 |
| Male | 15-19 years | Rate | 0 | 0 | 0 | 0 | 0 | 0 | 0 | 0 | 0 |
| Female | 15-19 years | Rate | 0 | 0 | 0 | 0 | 0 | 0 | 0 | 0 | 0 |
| Male | 20-24 years | Number | 118.5666539 | 153.6998509 | 68.18567378 | 82.79505695 | 105.1523098 | 48.66733827 | 5641.18662 | 7168.929411 | 3318.016382 |
| Female | 20-24 years | Number | 82.42963539 | 107.3207054 | 46.71824939 | 59.36001389 | 77.44745732 | 34.05977332 | 4041.667973 | 5275.428944 | 2317.444487 |
| Male | 20-24 years | Rate | 0.039078533 | 0.050658127 | 0.022473402 | 0.027288527 | 0.034657282 | 0.016040329 | 1.859285797 | 2.362816465 | 1.093589194 |
| Female | 20-24 years | Rate | 0.028060958 | 0.036534455 | 0.015903975 | 0.020207524 | 0.026364909 | 0.011594736 | 1.375877448 | 1.795878276 | 0.788911813 |
| Male | 25-29 years | Number | 201.8263025 | 246.5989346 | 122.5727008 | 128.7072914 | 154.4334336 | 78.83130578 | 8149.923936 | 9760.97252 | 4990.894493 |
| Female | 25-29 years | Number | 121.1812004 | 155.2207119 | 71.84620359 | 79.82624046 | 103.4084303 | 47.20357806 | 5055.855509 | 6546.539776 | 2990.359724 |
| Male | 25-29 years | Rate | 0.067873639 | 0.082930554 | 0.041220867 | 0.043283914 | 0.051935545 | 0.026510755 | 2.740797349 | 3.282588624 | 1.678424302 |
| Female | 25-29 years | Rate | 0.041644846 | 0.053342784 | 0.024690497 | 0.027432898 | 0.035537098 | 0.01622187 | 1.737483395 | 2.249768438 | 1.027659979 |
| Male | 30-34 years | Number | 403.8244708 | 485.8619831 | 281.5414049 | 257.487674 | 315.8388697 | 176.7088717 | 15068.11661 | 18467.32327 | 10344.10004 |
| Female | 30-34 years | Number | 266.5997177 | 335.1984331 | 176.5242913 | 168.4371994 | 217.2845982 | 111.1143556 | 9849.708745 | 12702.36485 | 6499.224981 |
| Male | 30-34 years | Rate | 0.132163441 | 0.159012631 | 0.092142709 | 0.084270418 | 0.103367564 | 0.057833178 | 4.93148454 | 6.043974943 | 3.385411114 |
| Female | 30-34 years | Rate | 0.089184404 | 0.112132424 | 0.059051877 | 0.056346539 | 0.072687239 | 0.037170586 | 3.294978756 | 4.249264971 | 2.174156495 |
| Male | 35-39 years | Number | 786.708233 | 977.7860807 | 590.2822229 | 474.1357198 | 601.052963 | 349.7425047 | 25476.42394 | 32360.48455 | 18829.56267 |
| Female | 35-39 years | Number | 555.4469878 | 708.844453 | 387.8652717 | 328.3785939 | 422.923507 | 222.9027178 | 17641.3211 | 22699.05314 | 11976.38004 |
| Male | 35-39 years | Rate | 0.277926539 | 0.345430097 | 0.208533594 | 0.167501615 | 0.212338657 | 0.123556256 | 9.000254525 | 11.43224018 | 6.652066122 |
| Female | 35-39 years | Rate | 0.199942952 | 0.255161079 | 0.139618954 | 0.118205674 | 0.152238785 | 0.08023777 | 6.35030507 | 8.170925035 | 4.311109495 |
| Male | 40-44 years | Number | 1487.369014 | 1854.646274 | 1129.690957 | 932.5729357 | 1204.936204 | 694.6683204 | 45493.2675 | 58878.73923 | 33856.03848 |
| Female | 40-44 years | Number | 1043.654535 | 1279.220434 | 766.4439607 | 632.2853391 | 793.3930565 | 448.102116 | 30836.30225 | 38661.80857 | 21847.72313 |
| Male | 40-44 years | Rate | 0.589849102 | 0.735501028 | 0.448003952 | 0.369832438 | 0.47784412 | 0.275486097 | 18.04136211 | 23.34966717 | 13.42636138 |
| Female | 40-44 years | Rate | 0.420676014 | 0.515627859 | 0.308938044 | 0.25486142 | 0.319800679 | 0.180620891 | 12.42948916 | 15.58379233 | 8.806374891 |
| Male | 45-49 years | Number | 2716.438559 | 3203.006036 | 2148.788453 | 1689.699997 | 2031.743377 | 1316.869143 | 74224.98101 | 89334.83534 | 57858.50014 |
| Female | 45-49 years | Number | 1982.894964 | 2401.471522 | 1559.091322 | 1196.56293 | 1459.2145 | 933.983368 | 52546.23668 | 63906.42935 | 41078.5193 |
| Male | 45-49 years | Rate | 1.142022683 | 1.346581367 | 0.90337591 | 0.71036973 | 0.854168785 | 0.553627258 | 31.20505402 | 37.55741429 | 24.32439318 |
| Female | 45-49 years | Rate | 0.841484164 | 1.019116137 | 0.661633965 | 0.507787238 | 0.619249086 | 0.396355948 | 22.2991267 | 27.12006901 | 17.43255396 |
| Male | 50-54 years | Number | 5011.651112 | 5903.254144 | 3996.870174 | 3080.153249 | 3753.958422 | 2416.77577 | 120786.9487 | 147113.7105 | 94876.1532 |
| Female | 50-54 years | Number | 3643.438644 | 4372.281967 | 2859.872704 | 2165.325808 | 2635.46882 | 1647.825486 | 84918.98916 | 103156.5177 | 64632.17874 |
| Male | 50-54 years | Rate | 2.257699667 | 2.659358087 | 1.800550808 | 1.387578826 | 1.691121446 | 1.088733778 | 54.41333558 | 66.27328354 | 42.74077638 |
| Female | 50-54 years | Rate | 1.634249886 | 1.961169654 | 1.282784505 | 0.971248263 | 1.182129038 | 0.739125556 | 38.09007421 | 46.27044498 | 28.99050623 |
| Male | 55-59 years | Number | 7546.384838 | 8582.575812 | 6041.247143 | 4889.266167 | 5741.967732 | 3886.20286 | 168489.3897 | 198253.973 | 133610.8554 |
| Female | 55-59 years | Number | 5619.448339 | 6773.713007 | 4444.544681 | 3633.206804 | 4482.411287 | 2836.563157 | 125206.931 | 154256.0486 | 97616.23683 |
| Male | 55-59 years | Rate | 3.875416605 | 4.407548453 | 3.102458992 | 2.510863638 | 2.948765214 | 1.995744375 | 86.52707126 | 101.8125573 | 68.61533553 |
| Female | 55-59 years | Rate | 2.795696429 | 3.369947391 | 2.211177494 | 1.807533885 | 2.230016271 | 1.411200711 | 62.29091339 | 76.74295737 | 48.56444054 |
| Male | 60-64 years | Number | 9943.226919 | 11251.72575 | 8380.808855 | 6768.749417 | 7803.212246 | 5650.77014 | 201516.5597 | 231859.5044 | 167715.9839 |
| Female | 60-64 years | Number | 7799.410004 | 9160.259931 | 6495.063639 | 5397.460239 | 6488.52588 | 4425.807227 | 160466.1784 | 193305.1057 | 131912.7545 |
| Male | 60-64 years | Rate | 6.392840295 | 7.2341189 | 5.388308342 | 4.351860254 | 5.016951749 | 3.633073181 | 129.561881 | 149.0703968 | 107.830336 |
| Female | 60-64 years | Rate | 4.740967584 | 5.568177 | 3.948104556 | 3.280912789 | 3.944130499 | 2.690281519 | 97.54134605 | 117.5028931 | 80.18479512 |
| Male | 65-69 years | Number | 12335.74813 | 13805.64005 | 10407.27524 | 8912.06439 | 10208.3729 | 7430.075263 | 224111.1511 | 256258.1811 | 186516.1573 |
| Female | 65-69 years | Number | 9503.685603 | 11102.39093 | 7996.354358 | 7145.252925 | 8488.72179 | 5830.935999 | 178922.9717 | 213201.0042 | 145868.6349 |
| Male | 65-69 years | Rate | 9.357085096 | 10.47204818 | 7.894272727 | 6.760104375 | 7.743398531 | 5.635965147 | 169.9959411 | 194.3805582 | 141.4788579 |
| Female | 65-69 years | Rate | 6.599372783 | 7.709516035 | 5.552679825 | 4.961673781 | 5.89458047 | 4.049010241 | 124.2443657 | 148.0470802 | 101.2913873 |
| Male | 70-74 years | Number | 13614.10908 | 15169.15615 | 11784.51828 | 10400.47071 | 11695.07616 | 8892.448676 | 216096.772 | 243554.692 | 185266.7436 |
| Female | 70-74 years | Number | 10352.70628 | 12018.83441 | 8843.543495 | 8316.757784 | 9761.994696 | 6956.506701 | 171775.3371 | 200997.263 | 143510.6756 |
| Male | 70-74 years | Rate | 14.12377799 | 15.73704108 | 12.22569314 | 10.78983123 | 12.13290259 | 9.225353652 | 224.1867473 | 252.6726045 | 192.2025408 |
| Female | 70-74 years | Rate | 9.459034821 | 10.98133861 | 8.080146739 | 7.598834486 | 8.919314939 | 6.356003673 | 156.9472611 | 183.6466774 | 131.1224758 |
| Male | 75-79 years | Number | 11324.9474 | 12423.84686 | 10031.07373 | 9222.432946 | 10260.79491 | 8184.220295 | 153593.5852 | 170845.876 | 136527.8585 |
| Female | 75-79 years | Number | 9113.814356 | 10503.32413 | 7854.899696 | 7777.051157 | 8967.1166 | 6599.029708 | 128553.2915 | 148595.7297 | 109031.6776 |
| Male | 75-79 years | Rate | 18.94224469 | 20.7802773 | 16.77809587 | 15.42555346 | 17.1623303 | 13.68902636 | 256.9024978 | 285.7588891 | 228.3581558 |
| Female | 75-79 years | Rate | 12.64092573 | 14.56818572 | 10.89480208 | 10.7868256 | 12.43745489 | 9.152901424 | 178.3043352 | 206.1033404 | 151.227717 |
| Male | 80-84 years | Number | 8873.37436 | 9700.001995 | 7752.939364 | 7970.853636 | 8679.478667 | 6954.991829 | 104093.7989 | 113437.1351 | 90941.74299 |
| Female | 80-84 years | Number | 7790.914398 | 9026.638336 | 6144.072925 | 7294.560498 | 8306.862572 | 5904.184858 | 94463.43391 | 107975.4735 | 76544.06752 |
| Male | 80-84 years | Rate | 24.20988682 | 26.46523645 | 21.15292074 | 21.74747245 | 23.68086679 | 18.97582117 | 284.0068489 | 309.4989673 | 248.1231171 |
| Female | 80-84 years | Rate | 15.2969402 | 17.72320164 | 12.06347693 | 14.32238246 | 16.30996999 | 11.59247273 | 185.4726449 | 212.0026324 | 150.2891655 |
| Male | 85-89 years | Number | 5617.28136 | 6201.356759 | 4744.718312 | 5479.787765 | 6031.919772 | 4693.2841 | 56684.24837 | 62314.46396 | 48678.905 |
| Female | 85-89 years | Number | 5155.618522 | 5977.467776 | 3863.719565 | 5203.924318 | 6001.434404 | 3977.679432 | 53426.52188 | 61641.75147 | 40884.23284 |
| Male | 85-89 years | Rate | 32.55893018 | 35.94435257 | 27.50137341 | 31.76198873 | 34.96226059 | 27.20325003 | 328.553684 | 361.1875837 | 282.1530501 |
| Female | 85-89 years | Rate | 18.10950401 | 20.99631232 | 13.57161021 | 18.27918182 | 21.08049693 | 13.97190295 | 187.6647406 | 216.5213623 | 143.6089919 |
| Male | 90-94 years | Number | 2068.243612 | 2305.615257 | 1702.408347 | 2232.575317 | 2455.177641 | 1837.747332 | 19973.2436 | 22052.1 | 16539.46886 |
| Female | 90-94 years | Number | 2631.14756 | 3104.948975 | 1947.884328 | 2862.344934 | 3318.489309 | 2124.856399 | 25588.54034 | 29709.93743 | 19058.63726 |
| Male | 90-94 years | Rate | 35.48501261 | 39.55761593 | 29.2083492 | 38.30446415 | 42.1236691 | 31.53037045 | 342.6824563 | 378.3495532 | 283.7689224 |
| Female | 90-94 years | Rate | 21.81556192 | 25.74397865 | 16.15044014 | 23.73248239 | 27.5144998 | 17.61776384 | 212.1615658 | 246.333193 | 158.0203587 |
| Male | 95+ years | Number | 404.3130699 | 456.6961039 | 309.9937502 | 599.967199 | 676.2913373 | 460.6936478 | 4923.56822 | 5545.187902 | 3790.107819 |
| Female | 95+ years | Number | 638.2230407 | 748.660549 | 445.5226525 | 977.1723995 | 1142.808262 | 687.8556501 | 7978.536613 | 9327.630415 | 5623.765289 |
| Male | 95+ years | Rate | 26.73954173 | 30.20393214 | 20.50166427 | 39.67927121 | 44.72702413 | 30.46831264 | 325.6237992 | 366.7350732 | 250.6615633 |
| Female | 95+ years | Rate | 16.20568322 | 19.00989924 | 11.31265798 | 24.81224485 | 29.01805087 | 17.4659485 | 202.5900487 | 236.8460773 | 142.7979765 |

Supplementary Table S8. Sex differences of incidence, mortality and disability-adjusted life years rates in multiple myeloma across 21 geographic regions in 2021.

| location | sex | incidence | | | mortality | | | disability-adjusted life years | | |
| --- | --- | --- | --- | --- | --- | --- | --- | --- | --- | --- |
|  |  | val | upper | lower | val | upper | lower | val | upper | lower |
| Global | Male | 2.082492079 | 2.291675657 | 1.804755168 | 1.594227811 | 1.772497877 | 1.374966715 | 36.47841309 | 40.78385596 | 30.78939845 |
| Global | Female | 1.686196767 | 1.914740662 | 1.42474805 | 1.353978219 | 1.548339213 | 1.140134608 | 29.27983191 | 34.00496549 | 23.91396091 |
| Central Asia | Male | 0.449777244 | 0.504987647 | 0.396518558 | 0.398909095 | 0.445863672 | 0.354718765 | 12.51548656 | 14.01908873 | 11.10917674 |
| Central Asia | Female | 0.469062196 | 0.527324756 | 0.414465069 | 0.408720984 | 0.460882346 | 0.359229067 | 12.403493 | 14.02276081 | 10.84223153 |
| Central Europe | Male | 4.391657645 | 4.807630176 | 3.910665649 | 3.880659387 | 4.251482525 | 3.486516868 | 87.32193369 | 95.46452693 | 78.39172742 |
| Central Europe | Female | 4.206371492 | 4.668701677 | 3.755603325 | 3.793915258 | 4.188803917 | 3.402334986 | 77.96862747 | 85.81688328 | 70.6824779 |
| Eastern Europe | Male | 2.731199611 | 3.022803128 | 2.407664655 | 2.00354177 | 2.215782037 | 1.775772248 | 53.99742888 | 59.91861083 | 47.7372448 |
| Eastern Europe | Female | 3.204213166 | 3.531809679 | 2.872304347 | 2.464720519 | 2.726342402 | 2.213810149 | 60.4352511 | 67.35501419 | 54.05777313 |
| Australasia | Male | 11.53371727 | 13.04320294 | 9.995415095 | 6.258499545 | 6.936981509 | 5.511398288 | 124.7445913 | 136.7682602 | 111.1837482 |
| Australasia | Female | 7.824921962 | 9.382202425 | 6.383624164 | 4.462419399 | 5.281349074 | 3.693363614 | 83.92035354 | 97.79276928 | 71.66231545 |
| High-income Asia Pacific | Male | 5.596054516 | 6.312203076 | 4.933752028 | 3.981577845 | 4.38776225 | 3.534239462 | 71.26564036 | 79.50823095 | 62.78328247 |
| High-income Asia Pacific | Female | 4.920054718 | 5.875660243 | 3.80736119 | 3.548073336 | 4.114400339 | 2.723983515 | 56.51596437 | 65.12219728 | 45.39419602 |
| High-income North America | Male | 6.750061422 | 7.064211106 | 6.281624783 | 5.976186435 | 6.251392175 | 5.554138663 | 118.4779528 | 123.3315898 | 112.544273 |
| High-income North America | Female | 4.577387323 | 4.890263285 | 4.057117059 | 4.5172951 | 4.837737778 | 3.946398502 | 84.18405966 | 89.06967476 | 76.40236442 |
| Southern Latin America | Male | 3.35079679 | 3.646361146 | 3.072476094 | 2.692415674 | 2.89492789 | 2.48823658 | 63.84373797 | 68.65345368 | 58.81568959 |
| Southern Latin America | Female | 2.671923148 | 2.957725973 | 2.356901706 | 2.267092125 | 2.467539703 | 1.995986501 | 48.1959948 | 52.15985848 | 43.7296051 |
| Western Europe | Male | 10.68816662 | 11.48788195 | 9.757421732 | 6.726071988 | 7.176905976 | 6.103330627 | 128.3456711 | 136.6564062 | 118.6817337 |
| Western Europe | Female | 8.186978651 | 8.926085868 | 6.93087438 | 5.582215208 | 6.11583318 | 4.728409915 | 97.23677147 | 105.0586535 | 84.63670235 |
| Andean Latin America | Male | 1.791366051 | 2.428416587 | 1.345462094 | 1.524315509 | 2.068019594 | 1.136171854 | 38.5903269 | 52.26581941 | 28.69001754 |
| Andean Latin America | Female | 1.416688306 | 1.904063849 | 1.070484656 | 1.145528921 | 1.496685131 | 0.888851283 | 28.57606788 | 37.21231876 | 21.87898896 |
| Caribbean | Male | 3.507558176 | 4.031279816 | 2.975648493 | 2.457893929 | 2.833418759 | 2.10079998 | 60.12696454 | 69.93353353 | 51.14579496 |
| Caribbean | Female | 3.703355306 | 4.342874885 | 3.117447105 | 2.220568497 | 2.596955658 | 1.894835237 | 53.30472566 | 62.97453288 | 45.48511222 |
| Central Latin America | Male | 1.746961934 | 1.99236516 | 1.524109712 | 1.44081445 | 1.643940153 | 1.260128443 | 38.93046973 | 44.4579118 | 33.8800764 |
| Central Latin America | Female | 1.534115399 | 1.721817185 | 1.349398215 | 1.198585008 | 1.340950537 | 1.057276682 | 31.43996089 | 35.44153239 | 27.71968836 |
| Tropical Latin America | Male | 2.426809028 | 2.581332045 | 2.278214232 | 2.090512238 | 2.217207866 | 1.961856959 | 53.17799938 | 56.07523278 | 49.9527823 |
| Tropical Latin America | Female | 2.333016844 | 2.511248328 | 2.110107697 | 1.943807613 | 2.081625518 | 1.756855986 | 46.7201028 | 49.58497581 | 42.995663 |
| North Africa and Middle East | Male | 1.032033936 | 1.394275046 | 0.704941987 | 0.836450806 | 1.135324407 | 0.575168918 | 22.18411784 | 30.02193578 | 15.27226387 |
| North Africa and Middle East | Female | 0.835416285 | 1.186991468 | 0.525894189 | 0.668647602 | 0.924217101 | 0.427660545 | 17.73656496 | 24.58294167 | 11.25599665 |
| South Asia | Male | 0.9705483 | 1.335674542 | 0.65289934 | 0.901303651 | 1.247633464 | 0.612144207 | 23.64185658 | 32.65622533 | 15.98276158 |
| South Asia | Female | 0.74788567 | 1.131094945 | 0.486429907 | 0.696769418 | 1.056993722 | 0.456856515 | 17.78582056 | 26.39272233 | 11.67456939 |
| East Asia | Male | 1.49911156 | 2.085438184 | 0.876082374 | 1.082095971 | 1.516385192 | 0.644920437 | 28.38893259 | 39.4919946 | 16.44853116 |
| East Asia | Female | 0.958740244 | 1.353516074 | 0.429645345 | 0.760928197 | 1.070152321 | 0.340651974 | 19.53000798 | 27.33616559 | 8.574909392 |
| Oceania | Male | 0.17714266 | 0.301763426 | 0.088637721 | 0.165671134 | 0.284117485 | 0.081979091 | 4.597840998 | 8.037496101 | 2.200466073 |
| Oceania | Female | 0.197857169 | 0.282971516 | 0.106534724 | 0.176654916 | 0.257453018 | 0.094519716 | 5.444794859 | 8.180406348 | 2.851936926 |
| Southeast Asia | Male | 0.508756616 | 0.870047483 | 0.355372021 | 0.434372954 | 0.742661518 | 0.301614805 | 11.94842886 | 20.38968538 | 8.306957309 |
| Southeast Asia | Female | 0.495892642 | 0.827873218 | 0.343622104 | 0.42077572 | 0.712810823 | 0.294271427 | 11.12950789 | 18.63084138 | 7.798766207 |
| Central Sub-Saharan Africa | Male | 0.179883271 | 0.314674682 | 0.097064688 | 0.170574723 | 0.301173522 | 0.09249928 | 5.077949348 | 9.00834574 | 2.753030806 |
| Central Sub-Saharan Africa | Female | 0.170073145 | 0.281939142 | 0.061561417 | 0.161463579 | 0.266690181 | 0.057156736 | 4.632973072 | 7.687125631 | 1.651286309 |
| Eastern Sub-Saharan Africa | Male | 0.487601358 | 0.741911906 | 0.275488059 | 0.465115138 | 0.708887249 | 0.264964384 | 13.43135706 | 20.81246105 | 7.457807197 |
| Eastern Sub-Saharan Africa | Female | 0.479039999 | 0.638313597 | 0.262161184 | 0.461577878 | 0.61566929 | 0.254417212 | 12.64493193 | 16.91793128 | 6.834384368 |
| Southern Sub-Saharan Africa | Male | 1.628225153 | 2.051423412 | 0.969921506 | 1.471055753 | 1.840029087 | 0.884051967 | 43.08006966 | 54.96713275 | 25.81746856 |
| Southern Sub-Saharan Africa | Female | 1.734942943 | 2.28137207 | 1.034488723 | 1.602606179 | 2.089472628 | 0.951023614 | 43.37650537 | 56.91563917 | 26.16121415 |
| Western Sub-Saharan Africa | Male | 0.124818498 | 0.17294133 | 0.0603763 | 0.120308121 | 0.165981177 | 0.059209382 | 3.281792151 | 4.588442613 | 1.55998484 |
| Western Sub-Saharan Africa | Female | 0.238190641 | 0.393150585 | 0.067837861 | 0.227939187 | 0.370048318 | 0.067163569 | 6.024742382 | 10.00914364 | 1.698991441 |

Supplementary Table S9. Predictions of the age-standardized incidence rates of multiple myeloma by Bayesian Age-Period-Cohort (BAPC) model.

| Time | val | low_50 | up_50 | low_60 | up_60 | low_70 | up_70 | low_80 | up_80 | low_95 | up_95 |
| --- | --- | --- | --- | --- | --- | --- | --- | --- | --- | --- | --- |
| 1990 | 2.342527924 | 2.336113575 | 2.348942273 | 2.334524263 | 2.350531585 | 2.33266848 | 2.352387369 | 2.330327338 | 2.354728511 | 2.323874921 | 2.361180928 |
| 1991 | 2.364037848 | 2.357932479 | 2.370143217 | 2.356419724 | 2.371655972 | 2.354653334 | 2.373422362 | 2.352424964 | 2.375650731 | 2.346283361 | 2.381792334 |
| 1992 | 2.392339917 | 2.386279619 | 2.398400214 | 2.384778032 | 2.399901801 | 2.383024682 | 2.401655151 | 2.380812763 | 2.40386707 | 2.374716499 | 2.409963334 |
| 1993 | 2.448761913 | 2.44268966 | 2.454834166 | 2.441185111 | 2.456338715 | 2.439428302 | 2.458095524 | 2.43721202 | 2.460311806 | 2.43110373 | 2.466420096 |
| 1994 | 2.51607645 | 2.509971111 | 2.522181788 | 2.508458364 | 2.523694536 | 2.506691983 | 2.525460917 | 2.504463625 | 2.527689275 | 2.498322053 | 2.533830847 |
| 1995 | 2.577106709 | 2.570975349 | 2.583238069 | 2.569456154 | 2.584757264 | 2.567682244 | 2.586531174 | 2.565444389 | 2.588769029 | 2.559276641 | 2.594936777 |
| 1996 | 2.612350566 | 2.606235096 | 2.618466036 | 2.604719839 | 2.619981294 | 2.602950526 | 2.621750606 | 2.60071847 | 2.623982662 | 2.594566707 | 2.630134425 |
| 1997 | 2.648714729 | 2.642614356 | 2.654815102 | 2.641102839 | 2.656326619 | 2.639337894 | 2.658091564 | 2.637111348 | 2.66031811 | 2.630974771 | 2.666454687 |
| 1998 | 2.697512437 | 2.6914096 | 2.703615274 | 2.689897473 | 2.705127401 | 2.688131815 | 2.706893059 | 2.68590437 | 2.709120504 | 2.679765314 | 2.71525956 |
| 1999 | 2.719793483 | 2.713724547 | 2.72586242 | 2.712220819 | 2.727366147 | 2.71046497 | 2.729121997 | 2.708249898 | 2.731337069 | 2.702144944 | 2.737442023 |
| 2000 | 2.742958754 | 2.736923452 | 2.748994056 | 2.735428059 | 2.75048945 | 2.73368194 | 2.752235569 | 2.731479144 | 2.754438364 | 2.725408025 | 2.760509484 |
| 2001 | 2.748177909 | 2.742203262 | 2.754152557 | 2.740722896 | 2.755632922 | 2.738994326 | 2.757361492 | 2.736813669 | 2.75954215 | 2.730803563 | 2.765552255 |
| 2002 | 2.754454941 | 2.748541475 | 2.760368407 | 2.74707627 | 2.761833613 | 2.7453654 | 2.763544482 | 2.743207073 | 2.765702809 | 2.737258513 | 2.771651369 |
| 2003 | 2.76019249 | 2.754337976 | 2.766047003 | 2.752887377 | 2.767497602 | 2.751193564 | 2.769191415 | 2.749056754 | 2.771328226 | 2.743167495 | 2.777217484 |
| 2004 | 2.750528706 | 2.744751749 | 2.756305663 | 2.743320367 | 2.757737045 | 2.741648992 | 2.75940842 | 2.739540488 | 2.761516923 | 2.733729247 | 2.767328165 |
| 2005 | 2.752472262 | 2.746754939 | 2.758189586 | 2.745338332 | 2.759606193 | 2.74368421 | 2.761260314 | 2.741597472 | 2.763347053 | 2.735846217 | 2.769098307 |
| 2006 | 2.750257956 | 2.744615368 | 2.755900545 | 2.743217278 | 2.757298634 | 2.741584779 | 2.758931134 | 2.739525318 | 2.760990595 | 2.733849242 | 2.76666667 |
| 2007 | 2.761844713 | 2.756265875 | 2.76742355 | 2.754883581 | 2.768805844 | 2.753269526 | 2.770419899 | 2.751233333 | 2.772456092 | 2.745621386 | 2.778068039 |
| 2008 | 2.76679633 | 2.761283019 | 2.772309641 | 2.759916961 | 2.773675699 | 2.758321864 | 2.775270796 | 2.756309587 | 2.777283073 | 2.750763556 | 2.782829104 |
| 2009 | 2.768625826 | 2.763183778 | 2.774067873 | 2.761835378 | 2.775416274 | 2.760260898 | 2.776990753 | 2.758274632 | 2.77897702 | 2.752800287 | 2.784451364 |
| 2010 | 2.790091983 | 2.784696836 | 2.795487131 | 2.783360056 | 2.79682391 | 2.781799145 | 2.798384821 | 2.779829996 | 2.80035397 | 2.77440283 | 2.805781136 |
| 2011 | 2.796636375 | 2.791307417 | 2.801965332 | 2.789987038 | 2.803285711 | 2.788445277 | 2.804827472 | 2.786500287 | 2.806772462 | 2.781139703 | 2.812133046 |
| 2012 | 2.796697735 | 2.791442887 | 2.801952583 | 2.79014087 | 2.803254601 | 2.788620551 | 2.80477492 | 2.786702609 | 2.806692862 | 2.781416575 | 2.811978896 |
| 2013 | 2.795826558 | 2.790643598 | 2.801009517 | 2.789359393 | 2.802293722 | 2.787859872 | 2.803793243 | 2.785968169 | 2.805684946 | 2.78075445 | 2.810898665 |
| 2014 | 2.799983893 | 2.794864746 | 2.80510304 | 2.793596352 | 2.806371434 | 2.792115293 | 2.807852493 | 2.790246881 | 2.809720905 | 2.785097353 | 2.814870433 |
| 2015 | 2.819710351 | 2.814637096 | 2.824783606 | 2.813380073 | 2.826040629 | 2.811912291 | 2.82750841 | 2.810060628 | 2.829360073 | 2.804957265 | 2.834463436 |
| 2016 | 2.837443425 | 2.832417063 | 2.842469788 | 2.831171659 | 2.843715192 | 2.829717444 | 2.845169407 | 2.827882896 | 2.847003954 | 2.822826704 | 2.852060147 |
| 2017 | 2.828331205 | 2.823385526 | 2.833276884 | 2.822160113 | 2.834502297 | 2.820729242 | 2.835933168 | 2.818924142 | 2.837738268 | 2.813949112 | 2.842713298 |
| 2018 | 2.822888913 | 2.818017686 | 2.827760141 | 2.81681072 | 2.828967107 | 2.815401388 | 2.830376439 | 2.813623462 | 2.832154364 | 2.808723325 | 2.837054502 |
| 2019 | 2.819918074 | 2.815116238 | 2.824719911 | 2.813926466 | 2.825909683 | 2.81253721 | 2.827298938 | 2.810784611 | 2.829051537 | 2.805954278 | 2.833881871 |
| 2020 | 2.781704871 | 2.776993568 | 2.786416174 | 2.775826227 | 2.787583515 | 2.774463164 | 2.788946577 | 2.772743609 | 2.790666133 | 2.768004345 | 2.795405396 |
| 2021 | 2.775232998 | 2.770490609 | 2.779975387 | 2.769315566 | 2.78115043 | 2.76794351 | 2.782522487 | 2.766212608 | 2.784253388 | 2.761442074 | 2.789023922 |
| 2022 | 2.785544565 | 2.762925604 | 2.808163525 | 2.757321203 | 2.813767926 | 2.750777141 | 2.820311988 | 2.742521556 | 2.828567573 | 2.719768359 | 2.851320771 |
| 2023 | 2.800693253 | 2.770426812 | 2.830959694 | 2.762927561 | 2.838458946 | 2.75417095 | 2.847215557 | 2.743124148 | 2.858262359 | 2.712678084 | 2.888708423 |
| 2024 | 2.816171879 | 2.779429829 | 2.852913928 | 2.770326087 | 2.862017671 | 2.759695969 | 2.872647789 | 2.746285666 | 2.886058092 | 2.709325563 | 2.923018195 |
| 2025 | 2.832250811 | 2.78964533 | 2.874856291 | 2.77908878 | 2.885412842 | 2.766762268 | 2.897739353 | 2.7512119 | 2.913289722 | 2.708353568 | 2.956148054 |
| 2026 | 2.849640475 | 2.801498418 | 2.897782533 | 2.789570045 | 2.909710906 | 2.775641705 | 2.923639246 | 2.758070568 | 2.941210383 | 2.709642801 | 2.98963815 |
| 2027 | 2.868047183 | 2.814522235 | 2.92157213 | 2.801260119 | 2.934834246 | 2.785774415 | 2.950319951 | 2.766238603 | 2.969855762 | 2.712396 | 3.023698365 |
| 2028 | 2.887232864 | 2.828479668 | 2.945986061 | 2.813922125 | 2.960543603 | 2.796923797 | 2.977541932 | 2.775479752 | 2.998985977 | 2.716377872 | 3.058087857 |
| 2029 | 2.906853031 | 2.843001021 | 2.970705042 | 2.827180122 | 2.98652594 | 2.808706618 | 3.004999445 | 2.785401582 | 3.028304481 | 2.721170628 | 3.092535435 |
| 2030 | 2.927425119 | 2.858549212 | 2.996301026 | 2.84148352 | 3.013366718 | 2.821556515 | 3.033293723 | 2.796417831 | 3.058432407 | 2.727133165 | 3.127717073 |
| 2031 | 2.949956116 | 2.876049751 | 3.023862482 | 2.85773764 | 3.042174593 | 2.836355234 | 3.063556998 | 2.809380508 | 3.090531725 | 2.735035529 | 3.164876704 |
| 2032 | 2.973962198 | 2.894961875 | 3.052962522 | 2.875387611 | 3.072536786 | 2.852531434 | 3.095392963 | 2.823697488 | 3.124226908 | 2.74422832 | 3.203696077 |
| 2033 | 2.998956238 | 2.914845026 | 3.08306745 | 2.894004414 | 3.103908062 | 2.869669567 | 3.128242908 | 2.838970223 | 3.158942252 | 2.754359834 | 3.243552641 |
| 2034 | 3.024687 | 2.935457237 | 3.113916764 | 2.913348379 | 3.136025622 | 2.887532646 | 3.161841355 | 2.854965107 | 3.194408894 | 2.76520579 | 3.284168211 |
| 2035 | 3.051783718 | 2.957391902 | 3.146175535 | 2.934004018 | 3.169563418 | 2.906694813 | 3.196872623 | 2.872243201 | 3.231324235 | 2.777291195 | 3.326276241 |

Supplementary Table S10. Predictions of the age-standardized mortality rates of multiple myeloma by Bayesian Age-Period-Cohort (BAPC) model.

| Time | val | low_50 | up_50 | low_60 | up_60 | low_70 | up_70 | low_80 | up_80 | low_95 | up_95 |
| --- | --- | --- | --- | --- | --- | --- | --- | --- | --- | --- | --- |
| 1990 | 2.063157188 | 2.057199369 | 2.069115008 | 2.055723173 | 2.070591204 | 2.053999471 | 2.072314906 | 2.051824956 | 2.074489421 | 2.045831778 | 2.080482599 |
| 1991 | 2.076682281 | 2.071113742 | 2.082250819 | 2.069734 | 2.083630561 | 2.068122925 | 2.085241636 | 2.066090491 | 2.08727407 | 2.060488905 | 2.092875656 |
| 1992 | 2.094348224 | 2.088839734 | 2.099856714 | 2.08747487 | 2.101221577 | 2.085881168 | 2.10281528 | 2.08387065 | 2.104825797 | 2.078329469 | 2.110366979 |
| 1993 | 2.131613462 | 2.126110458 | 2.137116465 | 2.124746954 | 2.138479969 | 2.123154839 | 2.140072084 | 2.121146324 | 2.142080599 | 2.115610662 | 2.147616261 |
| 1994 | 2.17435044 | 2.1688327 | 2.179868179 | 2.167465545 | 2.181235334 | 2.165869166 | 2.182831713 | 2.163855273 | 2.184845606 | 2.158304787 | 2.190396092 |
| 1995 | 2.206821188 | 2.201303984 | 2.212338392 | 2.199936962 | 2.213705415 | 2.198340738 | 2.215301638 | 2.196327041 | 2.217315336 | 2.190777094 | 2.222865283 |
| 1996 | 2.219832713 | 2.214352712 | 2.225312713 | 2.212994908 | 2.226670518 | 2.211409448 | 2.228255978 | 2.209409329 | 2.230256097 | 2.203896806 | 2.235768619 |
| 1997 | 2.237038879 | 2.23158856 | 2.242489197 | 2.230238111 | 2.243839647 | 2.228661238 | 2.245416519 | 2.226671953 | 2.247405805 | 2.221189288 | 2.252888469 |
| 1998 | 2.266508524 | 2.261067395 | 2.271949653 | 2.259719222 | 2.273297826 | 2.258145008 | 2.27487204 | 2.256159076 | 2.276857972 | 2.250685656 | 2.282331392 |
| 1999 | 2.282486578 | 2.277073588 | 2.287899568 | 2.275732387 | 2.289240769 | 2.274166314 | 2.290806842 | 2.272190653 | 2.292782503 | 2.266745538 | 2.298227618 |
| 2000 | 2.295828989 | 2.290449495 | 2.301208482 | 2.289116594 | 2.302541383 | 2.287560213 | 2.304097765 | 2.285596777 | 2.3060612 | 2.280185358 | 2.311472619 |
| 2001 | 2.292040281 | 2.286725282 | 2.29735528 | 2.285408361 | 2.298672202 | 2.283870638 | 2.300209924 | 2.281930742 | 2.30214982 | 2.2765842 | 2.307496362 |
| 2002 | 2.285781729 | 2.280534018 | 2.291029439 | 2.279233769 | 2.292329688 | 2.277715515 | 2.293847943 | 2.275800178 | 2.295763279 | 2.270521324 | 2.301042133 |
| 2003 | 2.274690888 | 2.269512741 | 2.279869035 | 2.268229729 | 2.281152047 | 2.2667316 | 2.282650176 | 2.264841653 | 2.284540122 | 2.259632776 | 2.289749 |
| 2004 | 2.247897548 | 2.242811708 | 2.252983387 | 2.241551567 | 2.254243528 | 2.240080145 | 2.25571495 | 2.238223889 | 2.257571206 | 2.233107867 | 2.262687228 |
| 2005 | 2.225667716 | 2.220665775 | 2.230669658 | 2.219426421 | 2.231909011 | 2.217979272 | 2.233356161 | 2.216153638 | 2.235181795 | 2.211122011 | 2.240213422 |
| 2006 | 2.203304728 | 2.19839312 | 2.208216336 | 2.197176149 | 2.209433307 | 2.195755135 | 2.210854322 | 2.193962471 | 2.212646986 | 2.189021714 | 2.217587743 |
| 2007 | 2.19659303 | 2.191754317 | 2.201431743 | 2.190555408 | 2.202630652 | 2.189155484 | 2.204030577 | 2.187389425 | 2.205796635 | 2.182521996 | 2.210664064 |
| 2008 | 2.190621824 | 2.185849911 | 2.195393736 | 2.184667553 | 2.196576095 | 2.183286955 | 2.197956692 | 2.181545278 | 2.19969837 | 2.176745046 | 2.204498602 |
| 2009 | 2.187680733 | 2.182971009 | 2.192390456 | 2.18180406 | 2.193557405 | 2.180441455 | 2.194920011 | 2.178722476 | 2.19663899 | 2.173984802 | 2.201376664 |
| 2010 | 2.198592858 | 2.1939284 | 2.203257315 | 2.192772667 | 2.204413049 | 2.191423158 | 2.205762558 | 2.1897207 | 2.207465016 | 2.18502856 | 2.212157155 |
| 2011 | 2.203360908 | 2.198750995 | 2.207970821 | 2.197608776 | 2.20911304 | 2.196275048 | 2.210446769 | 2.194592498 | 2.212129319 | 2.189955226 | 2.216766591 |
| 2012 | 2.203959843 | 2.199411141 | 2.208508545 | 2.198284088 | 2.209635597 | 2.196968069 | 2.210951617 | 2.195307861 | 2.212611825 | 2.190732163 | 2.217187523 |
| 2013 | 2.202842422 | 2.19835405 | 2.207330794 | 2.197241946 | 2.208442898 | 2.195943382 | 2.209741463 | 2.194305193 | 2.211379652 | 2.189790184 | 2.215894661 |
| 2014 | 2.208861326 | 2.204421973 | 2.213300679 | 2.203322014 | 2.214400637 | 2.202037632 | 2.21568502 | 2.200417334 | 2.217305318 | 2.195951634 | 2.221771017 |
| 2015 | 2.224272841 | 2.219871271 | 2.228674411 | 2.218780675 | 2.229765007 | 2.217507224 | 2.231038459 | 2.215900716 | 2.232644966 | 2.211473024 | 2.237072658 |
| 2016 | 2.238652142 | 2.234289052 | 2.243015231 | 2.23320799 | 2.244096293 | 2.231945672 | 2.245358612 | 2.230353209 | 2.246951074 | 2.225964226 | 2.251340058 |
| 2017 | 2.234315705 | 2.230020075 | 2.238611336 | 2.228955727 | 2.239675684 | 2.227712926 | 2.240918485 | 2.226145085 | 2.242486326 | 2.221823961 | 2.24680745 |
| 2018 | 2.230962283 | 2.226729039 | 2.235195527 | 2.225680149 | 2.236244417 | 2.224455397 | 2.237469169 | 2.222910325 | 2.23901424 | 2.218651958 | 2.243272608 |
| 2019 | 2.229365297 | 2.225189644 | 2.233540949 | 2.224155024 | 2.234575569 | 2.222946935 | 2.235783659 | 2.221422883 | 2.23730771 | 2.21722245 | 2.241508144 |
| 2020 | 2.206918552 | 2.202812196 | 2.211024908 | 2.201794746 | 2.212042358 | 2.200606705 | 2.213230398 | 2.199107946 | 2.214729157 | 2.19497722 | 2.218859883 |
| 2021 | 2.197497897 | 2.19331593 | 2.201679864 | 2.192279745 | 2.202716049 | 2.191069829 | 2.203925965 | 2.189543473 | 2.205452321 | 2.185336687 | 2.209659107 |
| 2022 | 2.196770818 | 2.181258005 | 2.21228363 | 2.177414326 | 2.216127309 | 2.172926198 | 2.220615438 | 2.167264251 | 2.226277384 | 2.151659375 | 2.241882261 |
| 2023 | 2.199002024 | 2.178326841 | 2.219677208 | 2.173204059 | 2.22479999 | 2.167222366 | 2.230781683 | 2.159676231 | 2.238327818 | 2.138878346 | 2.259125702 |
| 2024 | 2.20167638 | 2.176683488 | 2.226669272 | 2.170490887 | 2.232861872 | 2.163260006 | 2.240092754 | 2.154137971 | 2.249214788 | 2.128996754 | 2.274356006 |
| 2025 | 2.204728182 | 2.175872098 | 2.233584266 | 2.168722296 | 2.240734067 | 2.160373726 | 2.249082638 | 2.149841683 | 2.259614681 | 2.120814346 | 2.288642018 |
| 2026 | 2.208202339 | 2.175743212 | 2.240661465 | 2.167700669 | 2.248704008 | 2.158309676 | 2.258095002 | 2.146462576 | 2.269942101 | 2.113810814 | 2.302593863 |
| 2027 | 2.212140918 | 2.17621497 | 2.248066866 | 2.167313437 | 2.256968399 | 2.156919431 | 2.267362404 | 2.143806993 | 2.280474842 | 2.107667835 | 2.316614001 |
| 2028 | 2.216727982 | 2.177470069 | 2.255985894 | 2.16774296 | 2.265713003 | 2.156384959 | 2.277071004 | 2.142056403 | 2.29139956 | 2.102565506 | 2.330890457 |
| 2029 | 2.221775351 | 2.179300593 | 2.264250109 | 2.168776431 | 2.274774271 | 2.15648774 | 2.287062962 | 2.140985083 | 2.302565618 | 2.098258249 | 2.345292452 |
| 2030 | 2.227422819 | 2.181812369 | 2.273033268 | 2.170511264 | 2.284334374 | 2.157315362 | 2.297530275 | 2.140668225 | 2.314177413 | 2.09478709 | 2.360058548 |
| 2031 | 2.233828824 | 2.185119993 | 2.282537655 | 2.173051187 | 2.29460646 | 2.15895887 | 2.308698777 | 2.141180869 | 2.326476778 | 2.092182965 | 2.375474682 |
| 2032 | 2.240769096 | 2.188958991 | 2.292579201 | 2.176121769 | 2.305416423 | 2.161132198 | 2.320405993 | 2.142222279 | 2.339315913 | 2.090104695 | 2.391433496 |
| 2033 | 2.248399087 | 2.193507678 | 2.303290496 | 2.179906988 | 2.316891187 | 2.164025942 | 2.332772232 | 2.143991392 | 2.352806782 | 2.088774218 | 2.408023956 |
| 2034 | 2.256629248 | 2.19867997 | 2.314578526 | 2.184321618 | 2.328936879 | 2.167555877 | 2.345702619 | 2.146405251 | 2.366853246 | 2.08811206 | 2.425146437 |
| 2035 | 2.265739298 | 2.2047362 | 2.326742396 | 2.189621189 | 2.341857407 | 2.171971925 | 2.359506671 | 2.1497067 | 2.381771896 | 2.088341566 | 2.44313703 |

Supplementary Table S11. Predictions of the age-standardized disability-adjusted life years rates of multiple myeloma by Bayesian Age-Period-Cohort (BAPC) model.

| Time | val | low_50 | up_50 | low_60 | up_60 | low_70 | up_70 | low_80 | up_80 | low_95 | up_95 |
| --- | --- | --- | --- | --- | --- | --- | --- | --- | --- | --- | --- |
| 1990 | 45.059167 | 45.03003973 | 45.08829427 | 45.02282274 | 45.09551126 | 45.01439571 | 45.10393829 | 45.00376469 | 45.11456931 | 44.97446456 | 45.14386945 |
| 1991 | 45.26930843 | 45.24054154 | 45.29807531 | 45.23341384 | 45.30520301 | 45.22509108 | 45.31352577 | 45.2145916 | 45.32402526 | 45.18565399 | 45.35296286 |
| 1992 | 45.5759997 | 45.54749169 | 45.60450771 | 45.54042813 | 45.61157127 | 45.53218027 | 45.61981914 | 45.52177527 | 45.63022414 | 45.49309807 | 45.65890133 |
| 1993 | 46.46008388 | 46.43163412 | 46.48853364 | 46.42458499 | 46.49558276 | 46.41635398 | 46.50381378 | 46.40597024 | 46.51419752 | 46.37735163 | 46.54281612 |
| 1994 | 47.49800985 | 47.46957493 | 47.52644477 | 47.46252949 | 47.53349022 | 47.45430277 | 47.54171694 | 47.44392444 | 47.55209526 | 47.41532077 | 47.58069894 |
| 1995 | 48.2416213 | 48.21328062 | 48.26996199 | 48.20625852 | 48.27698408 | 48.19805907 | 48.28518354 | 48.18771514 | 48.29552747 | 48.15920626 | 48.32403634 |
| 1996 | 48.43197752 | 48.40389756 | 48.46005748 | 48.39694006 | 48.46701498 | 48.38881603 | 48.47513901 | 48.37856726 | 48.48538778 | 48.35032065 | 48.51363439 |
| 1997 | 48.80900749 | 48.78114359 | 48.83687139 | 48.77423963 | 48.84377535 | 48.76617811 | 48.85183687 | 48.7560082 | 48.86200678 | 48.72797894 | 48.89003604 |
| 1998 | 49.43457192 | 49.40684198 | 49.46230185 | 49.39997121 | 49.46917262 | 49.39194845 | 49.47719538 | 49.38182744 | 49.4873164 | 49.35393293 | 49.5152109 |
| 1999 | 49.65791284 | 49.63043846 | 49.68538722 | 49.623631 | 49.69219467 | 49.61568218 | 49.7001435 | 49.60565444 | 49.71017124 | 49.578017 | 49.73780867 |
| 2000 | 49.8676107 | 49.84038819 | 49.89483321 | 49.83364315 | 49.90157825 | 49.8257672 | 49.9094542 | 49.81583139 | 49.91939001 | 49.78844732 | 49.94677408 |
| 2001 | 49.6552949 | 49.6284538 | 49.682136 | 49.62180327 | 49.68878654 | 49.61403767 | 49.69655214 | 49.60424106 | 49.70634874 | 49.57724067 | 49.73334914 |
| 2002 | 49.47817547 | 49.45171793 | 49.504633 | 49.44516243 | 49.5111885 | 49.4375078 | 49.51884313 | 49.42785119 | 49.52849974 | 49.40123664 | 49.55511429 |
| 2003 | 49.22203346 | 49.1959705 | 49.24809643 | 49.18951276 | 49.25455417 | 49.18197228 | 49.26209465 | 49.17245969 | 49.27160724 | 49.14624204 | 49.29782489 |
| 2004 | 48.57988391 | 48.55433232 | 48.6054355 | 48.54800129 | 48.61176653 | 48.54060877 | 48.61915905 | 48.53128281 | 48.628485 | 48.50557958 | 48.65418823 |
| 2005 | 48.2230939 | 48.19796739 | 48.24822041 | 48.19174168 | 48.25444612 | 48.18447214 | 48.26171566 | 48.17530134 | 48.27088646 | 48.15002571 | 48.29616209 |
| 2006 | 47.70627784 | 47.68162657 | 47.73092911 | 47.67551861 | 47.73703707 | 47.66838656 | 47.74416912 | 47.65938921 | 47.75316647 | 47.63459164 | 47.77796404 |
| 2007 | 47.54958902 | 47.5253269 | 47.57385114 | 47.51931537 | 47.57986267 | 47.51229591 | 47.58688213 | 47.5034406 | 47.59573744 | 47.47903449 | 47.62014355 |
| 2008 | 47.4578328 | 47.43394064 | 47.48172496 | 47.42802077 | 47.48764483 | 47.42110835 | 47.49455725 | 47.41238807 | 47.50327753 | 47.38835412 | 47.52731149 |
| 2009 | 47.33027874 | 47.3067634 | 47.35379409 | 47.3009369 | 47.35962059 | 47.2941335 | 47.36642399 | 47.28555075 | 47.37500674 | 47.26189585 | 47.39866164 |
| 2010 | 47.56575013 | 47.54250142 | 47.58899884 | 47.53674098 | 47.59475928 | 47.53001472 | 47.60148554 | 47.52152929 | 47.60997097 | 47.4981426 | 47.63335766 |
| 2011 | 47.63630688 | 47.61337361 | 47.65924015 | 47.60769133 | 47.66492242 | 47.60105633 | 47.67155742 | 47.59268603 | 47.67992772 | 47.56961666 | 47.70299709 |
| 2012 | 47.63893519 | 47.61633887 | 47.66153151 | 47.61074008 | 47.6671303 | 47.60420257 | 47.67366781 | 47.59595525 | 47.68191513 | 47.57322483 | 47.70464555 |
| 2013 | 47.59394629 | 47.57168825 | 47.61620433 | 47.56617328 | 47.6217193 | 47.55973364 | 47.62815894 | 47.55160978 | 47.63628279 | 47.52921965 | 47.65867293 |
| 2014 | 47.67985958 | 47.65790183 | 47.70181733 | 47.65246126 | 47.7072579 | 47.64610849 | 47.71361066 | 47.63809424 | 47.72162492 | 47.61600617 | 47.74371298 |
| 2015 | 48.02655916 | 48.00482442 | 48.0482939 | 47.99943911 | 48.05367921 | 47.99315087 | 48.05996745 | 47.98521801 | 48.06790031 | 47.96335428 | 48.08976403 |
| 2016 | 48.33152455 | 48.31002458 | 48.35302452 | 48.30469743 | 48.35835167 | 48.29847711 | 48.36457199 | 48.29062994 | 48.37241916 | 48.26900237 | 48.39404673 |
| 2017 | 48.2779899 | 48.25681173 | 48.29916806 | 48.25156432 | 48.30441547 | 48.24543711 | 48.31054268 | 48.2377074 | 48.3182724 | 48.21640355 | 48.33957625 |
| 2018 | 48.26711709 | 48.24624216 | 48.28799202 | 48.24106988 | 48.2931643 | 48.2350304 | 48.29920378 | 48.22741136 | 48.30682282 | 48.20641254 | 48.32782164 |
| 2019 | 48.30339337 | 48.28280503 | 48.32398171 | 48.27770377 | 48.32908297 | 48.2717472 | 48.33503954 | 48.26423276 | 48.34255398 | 48.24352224 | 48.3632645 |
| 2020 | 47.77875301 | 47.75853328 | 47.79897275 | 47.75352334 | 47.80398268 | 47.74767342 | 47.8098326 | 47.74029352 | 47.81721251 | 47.71995378 | 47.83755224 |
| 2021 | 47.72911675 | 47.70912047 | 47.74911303 | 47.70416591 | 47.7540676 | 47.69838063 | 47.75985287 | 47.69108229 | 47.76715122 | 47.67096734 | 47.78726617 |
| 2022 | 47.44763131 | 46.99010831 | 47.9051543 | 46.87674579 | 48.01851682 | 46.74437638 | 48.15088624 | 46.57738727 | 48.31787534 | 46.11714901 | 48.7781136 |
| 2023 | 47.62491722 | 47.02927893 | 48.2205555 | 46.88169497 | 48.36813946 | 46.70936639 | 48.54046805 | 46.49196725 | 48.75786718 | 45.89279402 | 49.35704041 |
| 2024 | 47.81240886 | 47.09791704 | 48.52690067 | 46.92088421 | 48.7039335 | 46.71416922 | 48.91064849 | 46.45339031 | 49.17142741 | 45.73465819 | 49.89015952 |
| 2025 | 48.004047 | 47.18137326 | 48.82672074 | 46.97753571 | 49.03055829 | 46.73952179 | 49.26857221 | 46.43925808 | 49.56883592 | 45.61170201 | 50.39639199 |
| 2026 | 48.22325704 | 47.29837628 | 49.14813779 | 47.06921443 | 49.37729964 | 46.80163024 | 49.64488384 | 46.46406248 | 49.98245159 | 45.53369282 | 50.91282125 |
| 2027 | 48.4725314 | 47.44858826 | 49.49647453 | 47.19488129 | 49.75018151 | 46.89863661 | 50.04642619 | 46.52491256 | 50.42015024 | 45.49489261 | 51.45017019 |
| 2028 | 48.74756077 | 47.62708114 | 49.86804041 | 47.34945487 | 50.14566667 | 47.0252805 | 50.46984104 | 46.61632206 | 50.87879949 | 45.48919269 | 52.00592886 |
| 2029 | 49.03301154 | 47.81813452 | 50.24788855 | 47.517119 | 50.54890408 | 47.16563381 | 50.90038927 | 46.72222172 | 51.34380135 | 45.50013475 | 52.56588833 |
| 2030 | 49.31969093 | 48.01209916 | 50.6272827 | 47.68811129 | 50.95127057 | 47.30980209 | 51.32957976 | 46.8325505 | 51.80683136 | 45.51719854 | 53.12218332 |
| 2031 | 49.63774655 | 48.23755926 | 51.03793384 | 47.89062858 | 51.38486452 | 47.48552988 | 51.78996322 | 46.97448229 | 52.30101081 | 45.56598529 | 53.70950782 |
| 2032 | 49.99558753 | 48.50169538 | 51.48947968 | 48.13154702 | 51.85962803 | 47.69933787 | 52.29183719 | 47.1540894 | 52.83708566 | 45.65133142 | 54.33984364 |
| 2033 | 50.38363252 | 48.7953368 | 51.97192824 | 48.40179765 | 52.36546738 | 47.94227589 | 52.82498914 | 47.36257152 | 53.40469351 | 45.76484971 | 55.00241532 |
| 2034 | 50.78177937 | 49.09892554 | 52.4646332 | 48.68195731 | 52.88160144 | 48.19507823 | 53.36848051 | 47.58086155 | 53.98269719 | 45.88802046 | 55.67553829 |
| 2035 | 51.1777467 | 49.40038426 | 52.95510914 | 48.9599992 | 53.39549419 | 48.44577713 | 53.90971626 | 47.79706621 | 54.55842718 | 46.00915563 | 56.34633777 |

Supplementary Table S12. Predictions of the age-standardized incidence rates of multiple myeloma in diffent sex by Bayesian Age-Period-Cohort (BAPC) model.

| time | sex | val | low_50 | up_50 | low_60 | up_60 | low_70 | up_70 | low_80 | up_80 | low_95 | up_95 |
| --- | --- | --- | --- | --- | --- | --- | --- | --- | --- | --- | --- | --- |
| 1990 | male | 2.692019579 | 2.681964149 | 2.702075009 | 2.67947267 | 2.704566488 | 2.676563458 | 2.7074757 | 2.672893376 | 2.711145783 | 2.662778269 | 2.721260889 |
| 1990 | female | 2.080274034 | 2.072507425 | 2.088040644 | 2.070583057 | 2.089965012 | 2.068336041 | 2.092212028 | 2.065501343 | 2.095046726 | 2.057688641 | 2.102859428 |
| 1991 | male | 2.714817244 | 2.705489836 | 2.724144653 | 2.703178742 | 2.726455747 | 2.700480159 | 2.72915433 | 2.697075793 | 2.732558696 | 2.687693029 | 2.74194146 |
| 1991 | female | 2.102276929 | 2.095044403 | 2.109509454 | 2.093252368 | 2.11130149 | 2.091159872 | 2.113393986 | 2.088520107 | 2.11603375 | 2.081244658 | 2.123309199 |
| 1992 | male | 2.753799006 | 2.744593081 | 2.763004932 | 2.742312087 | 2.765285926 | 2.739648652 | 2.767949361 | 2.736288625 | 2.771309387 | 2.727028065 | 2.780569948 |
| 1992 | female | 2.124172551 | 2.116999199 | 2.131345904 | 2.115221825 | 2.133123278 | 2.113146449 | 2.135198654 | 2.110528281 | 2.137816822 | 2.103312357 | 2.145032746 |
| 1993 | male | 2.820024258 | 2.810813421 | 2.829235095 | 2.80853121 | 2.831517305 | 2.805866354 | 2.834182161 | 2.802504535 | 2.83754398 | 2.793239034 | 2.846809481 |
| 1993 | female | 2.170094212 | 2.16289907 | 2.177289354 | 2.161116297 | 2.179072127 | 2.159034616 | 2.181153807 | 2.156408496 | 2.183779928 | 2.149170653 | 2.191017771 |
| 1994 | male | 2.899292987 | 2.890024548 | 2.908561425 | 2.887728066 | 2.910857907 | 2.885046545 | 2.913539429 | 2.881663702 | 2.916922271 | 2.872340258 | 2.926245715 |
| 1994 | female | 2.223435163 | 2.216190794 | 2.230679533 | 2.214395824 | 2.232474503 | 2.212299901 | 2.234570426 | 2.209655813 | 2.237214513 | 2.20236845 | 2.244501876 |
| 1995 | male | 2.973393727 | 2.964070638 | 2.982716815 | 2.961760615 | 2.985026839 | 2.959063282 | 2.987724172 | 2.955660493 | 2.991126961 | 2.946282074 | 3.000505379 |
| 1995 | female | 2.271170489 | 2.263887544 | 2.278453434 | 2.262083016 | 2.280257962 | 2.259975933 | 2.282365046 | 2.257317766 | 2.285023213 | 2.249991598 | 2.29234938 |
| 1996 | male | 3.022590275 | 3.013274492 | 3.031906058 | 3.010966279 | 3.034214271 | 3.00827106 | 3.03690949 | 3.004870938 | 3.040309613 | 2.995499868 | 3.049680682 |
| 1996 | female | 2.298676504 | 2.291409235 | 2.305943772 | 2.289608591 | 2.307744416 | 2.287506043 | 2.309846964 | 2.284853598 | 2.312499409 | 2.2775432 | 2.319809807 |
| 1997 | male | 3.070235922 | 3.060932124 | 3.07953972 | 3.05862688 | 3.081844964 | 3.055935129 | 3.084536715 | 3.052539381 | 3.087932464 | 3.043180367 | 3.097291477 |
| 1997 | female | 2.326526278 | 2.319275129 | 2.333777426 | 2.31747848 | 2.335574076 | 2.315380596 | 2.33767196 | 2.312734034 | 2.340318521 | 2.305439852 | 2.347612703 |
| 1998 | male | 3.119973132 | 3.110665293 | 3.129280971 | 3.108359047 | 3.131587216 | 3.105666127 | 3.134280137 | 3.102268903 | 3.13767736 | 3.092905825 | 3.147040439 |
| 1998 | female | 2.370168137 | 2.362896973 | 2.377439301 | 2.361095364 | 2.37924091 | 2.358991689 | 2.381344585 | 2.356337822 | 2.383998452 | 2.349023506 | 2.391312768 |
| 1999 | male | 3.142267335 | 3.133005379 | 3.151529291 | 3.130710502 | 3.153824168 | 3.128030856 | 3.156503814 | 3.124650379 | 3.159884291 | 3.115333456 | 3.169201214 |
| 1999 | female | 2.3943052 | 2.387052418 | 2.401557982 | 2.385255364 | 2.403355036 | 2.383157007 | 2.405453393 | 2.380509849 | 2.408100551 | 2.373214024 | 2.415396376 |
| 2000 | male | 3.174157205 | 3.164929845 | 3.183384565 | 3.162643541 | 3.185670869 | 3.159973904 | 3.188340506 | 3.156606054 | 3.191708356 | 3.147323932 | 3.200990477 |
| 2000 | female | 2.408551992 | 2.401336691 | 2.415767293 | 2.399548923 | 2.417555061 | 2.39746141 | 2.419642574 | 2.394827932 | 2.422276052 | 2.38756981 | 2.429534174 |
| 2001 | male | 3.196522502 | 3.187359724 | 3.205685279 | 3.185089422 | 3.207955582 | 3.18243847 | 3.210606534 | 3.179094192 | 3.213950812 | 3.169877036 | 3.223167968 |
| 2001 | female | 2.400715087 | 2.393589675 | 2.4078405 | 2.391824179 | 2.409605995 | 2.389762672 | 2.411667502 | 2.387162003 | 2.414268172 | 2.379994303 | 2.421435872 |
| 2002 | male | 3.217654304 | 3.208560991 | 3.226747616 | 3.206307901 | 3.229000706 | 3.203677046 | 3.231631561 | 3.200358122 | 3.234950485 | 3.191210844 | 3.244097763 |
| 2002 | female | 2.394754775 | 2.387714642 | 2.401794908 | 2.385970277 | 2.403539273 | 2.383933443 | 2.405576107 | 2.381363899 | 2.408145651 | 2.374281985 | 2.415227565 |
| 2003 | male | 3.23424813 | 3.225222485 | 3.243273775 | 3.222986161 | 3.245510099 | 3.220374884 | 3.248121376 | 3.217080657 | 3.251415603 | 3.208001448 | 3.260494812 |
| 2003 | female | 2.389995928 | 2.383034078 | 2.396957779 | 2.381309109 | 2.398682748 | 2.379294924 | 2.400696933 | 2.376753951 | 2.403237906 | 2.369750784 | 2.410241073 |
| 2004 | male | 3.233565337 | 3.224630711 | 3.242499963 | 3.222416939 | 3.244713736 | 3.219831995 | 3.247298679 | 3.216570989 | 3.250559685 | 3.207583338 | 3.259547336 |
| 2004 | female | 2.375631495 | 2.368761926 | 2.382501065 | 2.367059822 | 2.384203169 | 2.365072335 | 2.386190656 | 2.362565044 | 2.388697947 | 2.355654705 | 2.395608285 |
| 2005 | male | 3.24385232 | 3.234993489 | 3.252711152 | 3.232798496 | 3.254906144 | 3.230235481 | 3.257469159 | 3.227002139 | 3.260702501 | 3.218090733 | 3.269613907 |
| 2005 | female | 2.369812348 | 2.363016636 | 2.37660806 | 2.361332832 | 2.378291864 | 2.359366714 | 2.380257982 | 2.35688638 | 2.382738316 | 2.350050337 | 2.389574359 |
| 2006 | male | 3.246721299 | 3.237966023 | 3.255476575 | 3.235796689 | 3.257645909 | 3.233263634 | 3.260178964 | 3.230068088 | 3.26337451 | 3.221260852 | 3.272181746 |
| 2006 | female | 2.363478863 | 2.356771857 | 2.370185869 | 2.355110032 | 2.371847694 | 2.353169577 | 2.373788148 | 2.35072162 | 2.376236106 | 2.343974809 | 2.382982917 |
| 2007 | male | 3.272487122 | 3.263810566 | 3.281163678 | 3.261660737 | 3.283313507 | 3.259150458 | 3.285823787 | 3.255983643 | 3.288990601 | 3.247255595 | 3.29771865 |
| 2007 | female | 2.360936969 | 2.354314563 | 2.367559376 | 2.3526737 | 2.369200239 | 2.350757722 | 2.371116217 | 2.348340641 | 2.373533297 | 2.341678933 | 2.380195006 |
| 2008 | male | 3.290747093 | 3.282146092 | 3.299348094 | 3.280014984 | 3.301479203 | 3.277526564 | 3.303967623 | 3.274387326 | 3.307106861 | 3.265735281 | 3.315758906 |
| 2008 | female | 2.354137476 | 2.347601621 | 2.360673331 | 2.345982203 | 2.362292749 | 2.344091266 | 2.364183686 | 2.341705776 | 2.366569176 | 2.335131133 | 2.373143819 |
| 2009 | male | 3.298609991 | 3.290103829 | 3.307116152 | 3.287996219 | 3.309223762 | 3.285535237 | 3.311684744 | 3.282430614 | 3.314789367 | 3.273873971 | 3.32334601 |
| 2009 | female | 2.351861261 | 2.345403527 | 2.358318996 | 2.343803465 | 2.359919057 | 2.34193513 | 2.361787393 | 2.339578153 | 2.36414437 | 2.333082094 | 2.370640429 |
| 2010 | male | 3.325619336 | 3.317178801 | 3.334059872 | 3.315087452 | 3.336151221 | 3.312645457 | 3.338593216 | 3.309564787 | 3.341673886 | 3.30107416 | 3.350164513 |
| 2010 | female | 2.365148045 | 2.358741655 | 2.371554435 | 2.357154315 | 2.373141775 | 2.355300834 | 2.374995256 | 2.352962597 | 2.377333493 | 2.346518187 | 2.383777903 |
| 2011 | male | 3.338282484 | 3.329935461 | 3.346629506 | 3.327867282 | 3.348697685 | 3.325452342 | 3.351112625 | 3.322405803 | 3.354159165 | 3.314009243 | 3.362555724 |
| 2011 | female | 2.365931014 | 2.359600354 | 2.372261674 | 2.358031778 | 2.37383025 | 2.356200207 | 2.375661821 | 2.35388961 | 2.377972418 | 2.34752138 | 2.384340649 |
| 2012 | male | 3.34700207 | 3.338755103 | 3.355249038 | 3.336711715 | 3.357292426 | 3.334325723 | 3.359678418 | 3.331315703 | 3.362688438 | 3.323019792 | 3.370984349 |
| 2012 | female | 2.358127996 | 2.351889953 | 2.364366039 | 2.350344325 | 2.365911667 | 2.34853955 | 2.367716442 | 2.346262757 | 2.369993235 | 2.339987692 | 2.376268299 |
| 2013 | male | 3.358688793 | 3.350533277 | 3.36684431 | 3.348512548 | 3.368865039 | 3.346153014 | 3.371224573 | 3.343176371 | 3.374201216 | 3.334972454 | 3.382405133 |
| 2013 | female | 2.346892795 | 2.340748201 | 2.353037389 | 2.339225728 | 2.354559862 | 2.337447989 | 2.356337601 | 2.335205304 | 2.358580286 | 2.329024244 | 2.364761346 |
| 2014 | male | 3.37262238 | 3.364547923 | 3.380696838 | 3.362547278 | 3.382697482 | 3.360211196 | 3.385033564 | 3.357264139 | 3.387980621 | 3.349141762 | 3.396102998 |
| 2014 | female | 2.343248235 | 2.337181888 | 2.349314583 | 2.335678802 | 2.350817669 | 2.333923702 | 2.352572769 | 2.331709575 | 2.354786896 | 2.325607225 | 2.360889245 |
| 2015 | male | 3.40446539 | 3.396445622 | 3.412485159 | 3.394458528 | 3.414472252 | 3.392138269 | 3.416792512 | 3.389211172 | 3.419719608 | 3.381143809 | 3.427786972 |
| 2015 | female | 2.350167 | 2.34416048 | 2.356173521 | 2.342672217 | 2.357661784 | 2.340934425 | 2.359399575 | 2.338742134 | 2.361591866 | 2.332699967 | 2.367634034 |
| 2016 | male | 3.432197662 | 3.42423851 | 3.440156814 | 3.422266435 | 3.442128889 | 3.419963713 | 3.444431611 | 3.417058741 | 3.447336583 | 3.409052354 | 3.45534297 |
| 2016 | female | 2.356305011 | 2.350358903 | 2.362251119 | 2.348885609 | 2.363724413 | 2.347165296 | 2.365444726 | 2.344995055 | 2.367614967 | 2.339013658 | 2.373596364 |
| 2017 | male | 3.434870526 | 3.427017517 | 3.442723534 | 3.425071742 | 3.44466931 | 3.422799729 | 3.446941323 | 3.419933497 | 3.449807554 | 3.412033883 | 3.457707168 |
| 2017 | female | 2.338621626 | 2.33278066 | 2.344462592 | 2.331333418 | 2.345909834 | 2.329643524 | 2.347599728 | 2.327511658 | 2.349731593 | 2.321636028 | 2.355607224 |
| 2018 | male | 3.432809889 | 3.425066331 | 3.440553448 | 3.423147675 | 3.442472104 | 3.420907328 | 3.444712451 | 3.418081044 | 3.447538735 | 3.410291529 | 3.455328249 |
| 2018 | female | 2.329535202 | 2.323781824 | 2.335288579 | 2.322356284 | 2.336714119 | 2.320691732 | 2.338378672 | 2.318591834 | 2.340478569 | 2.312804312 | 2.346266091 |
| 2019 | male | 3.433100343 | 3.425459398 | 3.440741289 | 3.423566167 | 3.44263452 | 3.421355508 | 3.444845179 | 3.418566676 | 3.447634011 | 3.410880384 | 3.455320303 |
| 2019 | female | 2.320912235 | 2.315240205 | 2.326584266 | 2.31383482 | 2.327989651 | 2.312193803 | 2.329630668 | 2.310123595 | 2.331700875 | 2.304417903 | 2.337406568 |
| 2020 | male | 3.394897881 | 3.387379759 | 3.402416003 | 3.38551696 | 3.404278802 | 3.383341836 | 3.406453927 | 3.380597833 | 3.40919793 | 3.373035092 | 3.41676067 |
| 2020 | female | 2.287638912 | 2.282067292 | 2.293210532 | 2.280686787 | 2.294591037 | 2.27907482 | 2.296203005 | 2.277041261 | 2.298236563 | 2.271436575 | 2.303841249 |
| 2021 | male | 3.387130778 | 3.37945808 | 3.394803476 | 3.377556981 | 3.396704575 | 3.375337135 | 3.398924421 | 3.372536714 | 3.401724842 | 3.364818481 | 3.409443076 |
| 2021 | female | 2.28366773 | 2.2779529 | 2.28938256 | 2.276536911 | 2.290798549 | 2.27488351 | 2.292451949 | 2.272797682 | 2.294537777 | 2.267048936 | 2.300286523 |
| 2022 | male | 3.410464048 | 3.38322612 | 3.437701975 | 3.376477256 | 3.444450839 | 3.368596847 | 3.452331248 | 3.358655407 | 3.462272688 | 3.33125583 | 3.489672265 |
| 2022 | female | 2.288582195 | 2.268799017 | 2.308365373 | 2.26389725 | 2.313267139 | 2.258173631 | 2.318990759 | 2.250953064 | 2.326211325 | 2.231052479 | 2.346111911 |
| 2023 | male | 3.433055715 | 3.397184685 | 3.468926745 | 3.388296759 | 3.47781467 | 3.377918643 | 3.488192787 | 3.364826249 | 3.501285181 | 3.328742335 | 3.537369095 |
| 2023 | female | 2.296744592 | 2.270181437 | 2.323307747 | 2.263599765 | 2.329889419 | 2.255914579 | 2.337574605 | 2.246219421 | 2.347269763 | 2.219498621 | 2.373990563 |
| 2024 | male | 3.455861259 | 3.412582626 | 3.499139892 | 3.401859286 | 3.509863232 | 3.38933802 | 3.522384498 | 3.373541961 | 3.538180557 | 3.330006482 | 3.581716036 |
| 2024 | female | 2.305484125 | 2.27321764 | 2.33775061 | 2.265222829 | 2.345745421 | 2.25588757 | 2.35508068 | 2.244110781 | 2.366857468 | 2.211652804 | 2.399315446 |
| 2025 | male | 3.479286144 | 3.429244055 | 3.529328233 | 3.416844903 | 3.541727385 | 3.40236685 | 3.556205438 | 3.38410223 | 3.574470058 | 3.333763156 | 3.624809132 |
| 2025 | female | 2.314949881 | 2.277542683 | 2.35235708 | 2.268274134 | 2.361625629 | 2.257451576 | 2.372448187 | 2.243798504 | 2.386101259 | 2.206169304 | 2.423730459 |
| 2026 | male | 3.504089704 | 3.447622117 | 3.560557291 | 3.43363089 | 3.574548518 | 3.417293828 | 3.590885579 | 3.396683997 | 3.611495411 | 3.33988129 | 3.668298117 |
| 2026 | female | 2.325362246 | 2.283126869 | 2.367597624 | 2.27266202 | 2.378062473 | 2.260442586 | 2.390281907 | 2.245027299 | 2.405697194 | 2.202541266 | 2.448183227 |
| 2027 | male | 3.530270404 | 3.467526371 | 3.593014436 | 3.451980001 | 3.608560806 | 3.433827054 | 3.626713753 | 3.410926413 | 3.649614394 | 3.347810013 | 3.712730794 |
| 2027 | female | 2.336369399 | 2.289469335 | 2.383269464 | 2.277848696 | 2.394890103 | 2.264279686 | 2.408459113 | 2.247161859 | 2.42557694 | 2.199983456 | 2.472755343 |
| 2028 | male | 3.557146627 | 3.488294259 | 3.625998995 | 3.471234399 | 3.643058855 | 3.451314204 | 3.662979051 | 3.426184111 | 3.688109143 | 3.356923123 | 3.757370131 |
| 2028 | female | 2.348213079 | 2.296794734 | 2.399631423 | 2.284054581 | 2.412371577 | 2.269178353 | 2.427247804 | 2.250411421 | 2.446014737 | 2.198687923 | 2.497738235 |
| 2029 | male | 3.584345806 | 3.509525438 | 3.659166173 | 3.490986861 | 3.677704751 | 3.469340019 | 3.699351593 | 3.442031695 | 3.726659917 | 3.366767289 | 3.801924322 |
| 2029 | female | 2.360830495 | 2.305006785 | 2.416654205 | 2.291175094 | 2.430485896 | 2.275024318 | 2.446636673 | 2.254649492 | 2.467011499 | 2.198494484 | 2.523166507 |
| 2030 | male | 3.612522755 | 3.531808228 | 3.693237282 | 3.511809228 | 3.713236282 | 3.488457103 | 3.736588408 | 3.458997498 | 3.766048013 | 3.377803953 | 3.847241558 |
| 2030 | female | 2.374559366 | 2.314396165 | 2.434722566 | 2.299489259 | 2.449629472 | 2.282082992 | 2.467035739 | 2.260124317 | 2.488994414 | 2.199604065 | 2.549514666 |
| 2031 | male | 3.642745958 | 3.556120064 | 3.729371852 | 3.534656378 | 3.750835538 | 3.50959399 | 3.775897926 | 3.477976824 | 3.807515092 | 3.390836829 | 3.894655087 |
| 2031 | female | 2.38973101 | 2.325236637 | 2.454225383 | 2.309256578 | 2.470205443 | 2.290597226 | 2.488864794 | 2.267057737 | 2.512404283 | 2.202180609 | 2.577281412 |
| 2032 | male | 3.67483698 | 3.582212495 | 3.767461465 | 3.559262512 | 3.790411449 | 3.532464627 | 3.817209334 | 3.498658064 | 3.851015897 | 3.405483879 | 3.944190082 |
| 2032 | female | 2.405799405 | 2.336941003 | 2.474657807 | 2.319879649 | 2.491719161 | 2.299957708 | 2.511641103 | 2.274825413 | 2.536773397 | 2.205558356 | 2.606040454 |
| 2033 | male | 3.707890279 | 3.609246844 | 3.806533715 | 3.584805518 | 3.830975041 | 3.556266245 | 3.859514314 | 3.520262855 | 3.895517704 | 3.421033998 | 3.99474656 |
| 2033 | female | 2.422927616 | 2.349690842 | 2.49616439 | 2.331544638 | 2.514310594 | 2.310355957 | 2.535499275 | 2.283625622 | 2.562229611 | 2.209954208 | 2.635901024 |
| 2034 | male | 3.741691593 | 3.637016595 | 3.84636659 | 3.611080802 | 3.872302383 | 3.580796492 | 3.902586693 | 3.542591671 | 3.940791514 | 3.437295457 | 4.046087728 |
| 2034 | female | 2.441103588 | 2.363470872 | 2.518736304 | 2.344235466 | 2.53797171 | 2.321774962 | 2.560432214 | 2.293440173 | 2.588767003 | 2.215346728 | 2.666860448 |
| 2035 | male | 3.777001081 | 3.666236778 | 3.887765384 | 3.63879221 | 3.915209952 | 3.606746158 | 3.947256004 | 3.566318831 | 3.987683331 | 3.454897173 | 4.099104989 |
| 2035 | female | 2.460789132 | 2.378711698 | 2.542866566 | 2.358375005 | 2.56320326 | 2.334628566 | 2.586949698 | 2.304671521 | 2.616906744 | 2.22210698 | 2.699471284 |

Supplementary Table S13. Predictions of the age-standardized mortality rates of multiple myeloma in diffent sex by Bayesian Age-Period-Cohort (BAPC) model.

| time | sex | val | low_50 | up_50 | low_60 | up_60 | low_70 | up_70 | low_80 | up_80 | low_95 | up_95 |
| --- | --- | --- | --- | --- | --- | --- | --- | --- | --- | --- | --- | --- |
| 1990 | male | 2.39188271 | 2.382493218 | 2.401272203 | 2.380166741 | 2.40359868 | 2.377450196 | 2.406315224 | 2.374023171 | 2.40974225 | 2.364577954 | 2.419187466 |
| 1990 | female | 1.824555225 | 1.817373933 | 1.831736518 | 1.815594592 | 1.833515859 | 1.813516918 | 1.835593532 | 1.810895853 | 1.838214598 | 1.803671942 | 1.845438509 |
| 1991 | male | 2.405562889 | 2.397006688 | 2.414119091 | 2.39488668 | 2.416239099 | 2.392411221 | 2.418714558 | 2.389288334 | 2.421837444 | 2.380681354 | 2.430444424 |
| 1991 | female | 1.839465426 | 1.832854244 | 1.846076609 | 1.831216161 | 1.847714691 | 1.829303431 | 1.849627422 | 1.826890447 | 1.852040405 | 1.820240029 | 1.858690823 |
| 1992 | male | 2.428640036 | 2.420275098 | 2.437004974 | 2.41820248 | 2.439077592 | 2.415782357 | 2.441497715 | 2.412729278 | 2.444550794 | 2.404314697 | 2.452965375 |
| 1992 | female | 1.854464581 | 1.847933804 | 1.860995357 | 1.846315644 | 1.862613517 | 1.844426177 | 1.864502985 | 1.84204254 | 1.866886621 | 1.835473005 | 1.873456156 |
| 1993 | male | 2.470569374 | 2.46224818 | 2.478890568 | 2.460186401 | 2.480952347 | 2.457778934 | 2.483359815 | 2.454741821 | 2.486396927 | 2.446371244 | 2.494767505 |
| 1993 | female | 1.884982387 | 1.878452764 | 1.891512009 | 1.87683489 | 1.893129883 | 1.874945756 | 1.895019017 | 1.872562541 | 1.897402232 | 1.865994167 | 1.903970606 |
| 1994 | male | 2.518142627 | 2.509806919 | 2.526478335 | 2.507741544 | 2.52854371 | 2.505329877 | 2.530955376 | 2.502287468 | 2.533997786 | 2.49390229 | 2.542382964 |
| 1994 | female | 1.919893039 | 1.913338393 | 1.926447685 | 1.911714319 | 1.928071759 | 1.909817945 | 1.929968133 | 1.907425596 | 1.932360481 | 1.90083205 | 1.938954027 |
| 1995 | male | 2.556200137 | 2.547862377 | 2.564537896 | 2.545796493 | 2.56660378 | 2.543384234 | 2.56901604 | 2.540341075 | 2.572059199 | 2.531953833 | 2.58044644 |
| 1995 | female | 1.94645783 | 1.939896754 | 1.953018907 | 1.938271087 | 1.954644574 | 1.936372853 | 1.956542808 | 1.933978157 | 1.958937504 | 1.927378143 | 1.965537518 |
| 1996 | male | 2.578244816 | 2.569952542 | 2.58653709 | 2.567897928 | 2.588591704 | 2.565498828 | 2.590990805 | 2.562472271 | 2.594017362 | 2.554130784 | 2.602358848 |
| 1996 | female | 1.955908393 | 1.949388226 | 1.96242856 | 1.947772695 | 1.964044091 | 1.945886296 | 1.965930489 | 1.943506532 | 1.968310254 | 1.936947669 | 1.974869116 |
| 1997 | male | 2.59999826 | 2.591749168 | 2.608247352 | 2.589705254 | 2.610291266 | 2.587318647 | 2.612677873 | 2.584307851 | 2.615688669 | 2.576009803 | 2.623986717 |
| 1997 | female | 1.970031619 | 1.963542061 | 1.976521178 | 1.961934114 | 1.978129124 | 1.960056572 | 1.980006667 | 1.957687979 | 1.98237526 | 1.951159907 | 1.988903331 |
| 1998 | male | 2.626535035 | 2.618303334 | 2.634766736 | 2.616263728 | 2.636806341 | 2.613882153 | 2.639187916 | 2.610877704 | 2.642192365 | 2.60259715 | 2.650472919 |
| 1998 | female | 1.998413533 | 1.991921872 | 2.004905194 | 1.990313404 | 2.006513662 | 1.988435253 | 2.008391814 | 1.986065893 | 2.010761174 | 1.979535706 | 2.017291361 |
| 1999 | male | 2.638734467 | 2.630546313 | 2.646922621 | 2.628517498 | 2.648951437 | 2.626148521 | 2.651320413 | 2.623159967 | 2.654308968 | 2.614923218 | 2.662545717 |
| 1999 | female | 2.017633872 | 2.011152897 | 2.024114847 | 2.009547077 | 2.025720667 | 2.007672017 | 2.027595727 | 2.005306557 | 2.029961187 | 1.998787119 | 2.036480625 |
| 2000 | male | 2.656523268 | 2.64837283 | 2.664673707 | 2.64635336 | 2.666693177 | 2.643995295 | 2.669051242 | 2.641020506 | 2.672026031 | 2.632821697 | 2.68022484 |
| 2000 | female | 2.025803666 | 2.019359129 | 2.032248204 | 2.017762337 | 2.033844996 | 2.015897819 | 2.035709514 | 2.013545658 | 2.038061674 | 2.007062874 | 2.044544458 |
| 2001 | male | 2.665181509 | 2.657100793 | 2.673262226 | 2.655098597 | 2.675264422 | 2.652760704 | 2.677602315 | 2.649811363 | 2.680551656 | 2.641682689 | 2.68868033 |
| 2001 | female | 2.01277884 | 2.006430417 | 2.019127262 | 2.00485744 | 2.02070024 | 2.00302073 | 2.022536949 | 2.00070365 | 2.024854029 | 1.994317552 | 2.031240128 |
| 2002 | male | 2.669346784 | 2.661344835 | 2.677348734 | 2.659362156 | 2.679331413 | 2.657047052 | 2.681646517 | 2.654126459 | 2.68456711 | 2.64607702 | 2.692616548 |
| 2002 | female | 1.997585806 | 1.991333099 | 2.003838513 | 1.989783837 | 2.005387774 | 1.98797482 | 2.007196791 | 1.985692674 | 2.009478937 | 1.979402859 | 2.015768752 |
| 2003 | male | 2.663972569 | 2.656058818 | 2.671886321 | 2.654097992 | 2.673847147 | 2.651808405 | 2.676136734 | 2.648920003 | 2.679025136 | 2.640959286 | 2.686985853 |
| 2003 | female | 1.98084849 | 1.974687241 | 1.987009738 | 1.973160641 | 1.988536338 | 1.971378084 | 1.990318895 | 1.96912932 | 1.99256766 | 1.962931506 | 1.998765473 |
| 2004 | male | 2.641792744 | 2.633997404 | 2.649588084 | 2.632065918 | 2.65151957 | 2.62981059 | 2.653774899 | 2.626965406 | 2.656620082 | 2.619123803 | 2.664461685 |
| 2004 | female | 1.953248662 | 1.94719936 | 1.959297964 | 1.945700497 | 1.960796827 | 1.943950328 | 1.962546996 | 1.941742422 | 1.964754902 | 1.935657219 | 1.970840105 |
| 2005 | male | 2.624161549 | 2.616476226 | 2.631846873 | 2.614571998 | 2.6337511 | 2.6123485 | 2.635974599 | 2.609543471 | 2.638779628 | 2.601812537 | 2.646510562 |
| 2005 | female | 1.927873235 | 1.921929323 | 1.933817147 | 1.920456573 | 1.935289896 | 1.918736895 | 1.937009574 | 1.916567455 | 1.939179014 | 1.910588268 | 1.945158201 |
| 2006 | male | 2.602956451 | 2.59539822 | 2.610514683 | 2.593525483 | 2.61238742 | 2.591338754 | 2.614574148 | 2.588580112 | 2.617332791 | 2.580977025 | 2.624935878 |
| 2006 | female | 1.90525686 | 1.89942116 | 1.911092559 | 1.897975222 | 1.912538497 | 1.896286852 | 1.914226867 | 1.894156908 | 1.916356811 | 1.888286575 | 1.922227144 |
| 2007 | male | 2.604697845 | 2.597236295 | 2.612159394 | 2.595387514 | 2.614008175 | 2.593228757 | 2.616166932 | 2.590505403 | 2.618890287 | 2.582999571 | 2.626396118 |
| 2007 | female | 1.889875918 | 1.88413695 | 1.895614886 | 1.882714981 | 1.897036856 | 1.881054597 | 1.89869724 | 1.878959958 | 1.900791878 | 1.873186931 | 1.906564905 |
| 2008 | male | 2.606143544 | 2.598765291 | 2.613521797 | 2.596937148 | 2.61534994 | 2.59480249 | 2.617484598 | 2.592109537 | 2.620177551 | 2.584687496 | 2.627599593 |
| 2008 | female | 1.876916918 | 1.871263831 | 1.882570006 | 1.86986314 | 1.883970697 | 1.868227603 | 1.885606234 | 1.86616431 | 1.887669527 | 1.860477673 | 1.893356164 |
| 2009 | male | 2.604926272 | 2.597632485 | 2.612220059 | 2.59582527 | 2.614027273 | 2.59371505 | 2.616137494 | 2.591052926 | 2.618799618 | 2.583715852 | 2.626136692 |
| 2009 | female | 1.872508617 | 1.86692431 | 1.878092924 | 1.865540661 | 1.879476573 | 1.863925023 | 1.881092211 | 1.861886834 | 1.883130401 | 1.856269385 | 1.888747849 |
| 2010 | male | 2.618036072 | 2.610806502 | 2.625265642 | 2.609015199 | 2.627056945 | 2.606923557 | 2.629148586 | 2.604284872 | 2.631787272 | 2.597012396 | 2.639059747 |
| 2010 | female | 1.878746426 | 1.873213423 | 1.88427943 | 1.871842486 | 1.885650367 | 1.870241691 | 1.887251161 | 1.868222227 | 1.889270625 | 1.862656387 | 1.894836465 |
| 2011 | male | 2.627056193 | 2.619902498 | 2.634209887 | 2.618129995 | 2.63598239 | 2.616060306 | 2.638052079 | 2.613449313 | 2.640663072 | 2.606253164 | 2.647859222 |
| 2011 | female | 1.879260712 | 1.873791236 | 1.884730187 | 1.87243604 | 1.886085383 | 1.870853625 | 1.887667798 | 1.868857348 | 1.889664075 | 1.863355413 | 1.89516601 |
| 2012 | male | 2.631341034 | 2.624272256 | 2.638409813 | 2.622520793 | 2.640161276 | 2.620475671 | 2.642206398 | 2.617895672 | 2.644786397 | 2.610784942 | 2.651897127 |
| 2012 | female | 1.876329468 | 1.870932134 | 1.881726802 | 1.869594812 | 1.883064124 | 1.868033269 | 1.884625667 | 1.866063322 | 1.886595614 | 1.860633956 | 1.89202498 |
| 2013 | male | 2.639492757 | 2.632497328 | 2.646488186 | 2.63076404 | 2.648221475 | 2.62874014 | 2.650245375 | 2.626186912 | 2.652798603 | 2.619149967 | 2.659835548 |
| 2013 | female | 1.868168345 | 1.862848875 | 1.873487815 | 1.861530846 | 1.874805845 | 1.85999183 | 1.87634486 | 1.858050302 | 1.878286388 | 1.852699262 | 1.883637428 |
| 2014 | male | 2.653635743 | 2.646698285 | 2.6605732 | 2.644979361 | 2.662292125 | 2.642972233 | 2.664299253 | 2.640440164 | 2.666831322 | 2.633461534 | 2.673809951 |
| 2014 | female | 1.867225941 | 1.861968601 | 1.87248328 | 1.860665967 | 1.873785915 | 1.859144926 | 1.875306955 | 1.857226075 | 1.877225806 | 1.851937535 | 1.882514346 |
| 2015 | male | 2.676940189 | 2.670046452 | 2.683833925 | 2.66833836 | 2.685542017 | 2.666343882 | 2.687536496 | 2.66382777 | 2.690052607 | 2.656893121 | 2.696987256 |
| 2015 | female | 1.874030521 | 1.868820891 | 1.879240151 | 1.867530077 | 1.880530964 | 1.86602284 | 1.882038202 | 1.864121402 | 1.883939639 | 1.858880854 | 1.889180187 |
| 2016 | male | 2.697840918 | 2.690995343 | 2.704686492 | 2.689299185 | 2.70638265 | 2.68731864 | 2.708363195 | 2.684820108 | 2.710861728 | 2.677933907 | 2.717747928 |
| 2016 | female | 1.880336831 | 1.875176412 | 1.88549725 | 1.873897792 | 1.88677587 | 1.872404793 | 1.88826887 | 1.870521317 | 1.890152346 | 1.865330272 | 1.895343391 |
| 2017 | male | 2.701535636 | 2.694776793 | 2.708294479 | 2.693102125 | 2.709969148 | 2.691146673 | 2.711924599 | 2.688679796 | 2.714391477 | 2.681880841 | 2.721190431 |
| 2017 | female | 1.870205314 | 1.86512977 | 1.875280857 | 1.86387218 | 1.876538448 | 1.862403736 | 1.878006891 | 1.860551238 | 1.879859389 | 1.855445572 | 1.884965055 |
| 2018 | male | 2.701570861 | 2.694901179 | 2.708240542 | 2.693248602 | 2.709893119 | 2.691318947 | 2.711822775 | 2.688884612 | 2.71425711 | 2.682175348 | 2.720966374 |
| 2018 | female | 1.863645952 | 1.85864283 | 1.868649074 | 1.857403184 | 1.86988872 | 1.855955693 | 1.871336211 | 1.854129627 | 1.873162277 | 1.849096813 | 1.878195091 |
| 2019 | male | 2.702597123 | 2.696007924 | 2.709186322 | 2.694375288 | 2.710818957 | 2.692468918 | 2.712725327 | 2.690063958 | 2.715130287 | 2.683435654 | 2.721758591 |
| 2019 | female | 1.857608634 | 1.852673608 | 1.862543659 | 1.851450834 | 1.863766433 | 1.850023045 | 1.865194222 | 1.848221834 | 1.866995433 | 1.84325752 | 1.871959747 |
| 2020 | male | 2.681589698 | 2.675082822 | 2.688096573 | 2.673470585 | 2.689708811 | 2.671588032 | 2.691591364 | 2.669213119 | 2.693966277 | 2.662667627 | 2.700511769 |
| 2020 | female | 1.83707975 | 1.83221759 | 1.84194191 | 1.831012871 | 1.843146629 | 1.829606163 | 1.844553337 | 1.827831547 | 1.846327953 | 1.822940532 | 1.851218968 |
| 2021 | male | 2.67149754 | 2.664763042 | 2.678232038 | 2.663094405 | 2.679900674 | 2.661145997 | 2.681849082 | 2.658688006 | 2.684307074 | 2.65191354 | 2.691081539 |
| 2021 | female | 1.82973371 | 1.824697568 | 1.834769853 | 1.82344974 | 1.83601768 | 1.821992696 | 1.837474725 | 1.820154578 | 1.839312842 | 1.815088548 | 1.844378873 |
| 2022 | male | 2.676678056 | 2.657758114 | 2.695597998 | 2.653070235 | 2.700285877 | 2.647596364 | 2.705759747 | 2.640690866 | 2.712665245 | 2.621658639 | 2.731697472 |
| 2022 | female | 1.826914489 | 1.811857615 | 1.841971364 | 1.808126905 | 1.845702073 | 1.803770688 | 1.85005829 | 1.798275152 | 1.855553826 | 1.783128919 | 1.870700059 |
| 2023 | male | 2.682073688 | 2.65761689 | 2.706530485 | 2.65155712 | 2.712590255 | 2.64448134 | 2.719666035 | 2.635554972 | 2.728592403 | 2.610953031 | 2.753194345 |
| 2023 | female | 1.826133043 | 1.806189573 | 1.846076513 | 1.80124809 | 1.851017996 | 1.795478095 | 1.856787991 | 1.788199025 | 1.864067062 | 1.768137196 | 1.884128891 |
| 2024 | male | 2.687705232 | 2.658515588 | 2.716894876 | 2.651283139 | 2.724127325 | 2.642838064 | 2.7325724 | 2.632184277 | 2.743226187 | 2.602821401 | 2.772589064 |
| 2024 | female | 1.825933198 | 1.801891758 | 1.849974638 | 1.795934902 | 1.855931493 | 1.788979293 | 1.862887103 | 1.780204524 | 1.871661871 | 1.756020405 | 1.895845991 |
| 2025 | male | 2.69365663 | 2.660177956 | 2.727135303 | 2.651882795 | 2.735430464 | 2.642196829 | 2.74511643 | 2.62997761 | 2.757335649 | 2.59630025 | 2.791013009 |
| 2025 | female | 1.826203957 | 1.798493311 | 1.853914602 | 1.79162732 | 1.860780593 | 1.783610146 | 1.868797768 | 1.773496171 | 1.878911742 | 1.745621071 | 1.906786843 |
| 2026 | male | 2.700229784 | 2.662709312 | 2.737750255 | 2.653412697 | 2.747046871 | 2.642557367 | 2.7579022 | 2.628862952 | 2.771596615 | 2.591119807 | 2.809339761 |
| 2026 | female | 1.826810636 | 1.79568938 | 1.857931892 | 1.787978327 | 1.865642945 | 1.778974403 | 1.87464687 | 1.767615606 | 1.886005666 | 1.736309654 | 1.917311618 |
| 2027 | male | 2.707610311 | 2.666170865 | 2.749049757 | 2.655903228 | 2.759317394 | 2.643914071 | 2.771306551 | 2.628789288 | 2.786431334 | 2.587103911 | 2.828116711 |
| 2027 | female | 1.82772403 | 1.793346181 | 1.86210188 | 1.784828227 | 1.870619834 | 1.774882113 | 1.880565948 | 1.762334708 | 1.893113353 | 1.727752836 | 1.927695225 |
| 2028 | male | 2.715508784 | 2.670293255 | 2.760724314 | 2.659090001 | 2.771927568 | 2.646008357 | 2.785009212 | 2.629505359 | 2.801512209 | 2.584021489 | 2.84699608 |
| 2028 | female | 1.829300437 | 1.791795339 | 1.866805535 | 1.782502532 | 1.876098341 | 1.771651651 | 1.886949222 | 1.757962847 | 1.900638027 | 1.720235166 | 1.938365707 |
| 2029 | male | 2.723615318 | 2.674749081 | 2.772481556 | 2.662641274 | 2.784589363 | 2.648503416 | 2.798727221 | 2.630667964 | 2.816562673 | 2.581511719 | 2.865718918 |
| 2029 | female | 1.831577544 | 1.79104462 | 1.872110468 | 1.781001596 | 1.882153492 | 1.769274712 | 1.893880377 | 1.754480796 | 1.908674293 | 1.713707321 | 1.949447768 |
| 2030 | male | 2.732243766 | 2.679808483 | 2.784679048 | 2.666816359 | 2.797671173 | 2.651645913 | 2.812841618 | 2.632507813 | 2.831979718 | 2.579761342 | 2.884726189 |
| 2030 | female | 1.83452286 | 1.791033553 | 1.878012167 | 1.780258012 | 1.888787707 | 1.767675794 | 1.901369925 | 1.751802843 | 1.917242877 | 1.708055439 | 1.96099028 |
| 2031 | male | 2.741792106 | 2.685814697 | 2.797769516 | 2.671944924 | 2.811639289 | 2.655749679 | 2.827834533 | 2.635318756 | 2.848265457 | 2.579009136 | 2.904575076 |
| 2031 | female | 1.838027408 | 1.791625112 | 1.884429705 | 1.780127807 | 1.89592701 | 1.766702811 | 1.909352006 | 1.749766661 | 1.926288156 | 1.70308898 | 1.972965837 |
| 2032 | male | 2.75220369 | 2.692665331 | 2.811742049 | 2.677913245 | 2.826494135 | 2.660687756 | 2.843719625 | 2.638957138 | 2.865450242 | 2.579065436 | 2.925341944 |
| 2032 | female | 1.8419216 | 1.792621348 | 1.891221853 | 1.780406004 | 1.903437197 | 1.766142578 | 1.917700623 | 1.748148717 | 1.935694484 | 1.698555882 | 1.985287319 |
| 2033 | male | 2.763117794 | 2.700044205 | 2.826191383 | 2.684416179 | 2.841819409 | 2.666167885 | 2.860067703 | 2.643146961 | 2.883088627 | 2.579699047 | 2.946536541 |
| 2033 | female | 1.846610636 | 1.794427035 | 1.898794237 | 1.78149727 | 1.911724001 | 1.766399641 | 1.926821631 | 1.747353401 | 1.945867871 | 1.694860105 | 1.998361167 |
| 2034 | male | 2.774399825 | 2.70782157 | 2.840978081 | 2.691325177 | 2.857474474 | 2.672062922 | 2.876736729 | 2.647762846 | 2.901036805 | 2.580789467 | 2.968010184 |
| 2034 | female | 1.852162325 | 1.797102295 | 1.907222356 | 1.783459824 | 1.920864827 | 1.767529993 | 1.936794658 | 1.747433899 | 1.956890752 | 1.692047102 | 2.012277549 |
| 2035 | male | 2.786527418 | 2.716445961 | 2.856608875 | 2.699081564 | 2.873973272 | 2.678805772 | 2.894249064 | 2.65322708 | 2.919827756 | 2.582729709 | 2.990325127 |
| 2035 | female | 1.858638184 | 1.800695465 | 1.916580904 | 1.786338738 | 1.930937631 | 1.769574894 | 1.947701474 | 1.748426661 | 1.968849707 | 1.690140068 | 2.027136301 |

Supplementary Table S14. Predictions of the age-standardized disability-adjusted life years rates of multiple myeloma in diffent sex by Bayesian Age-Period-Cohort (BAPC) model.

| time | sex | val | low_50 | up_50 | low_60 | up_60 | low_70 | up_70 | low_80 | up_80 | low_95 | up_95 |
| --- | --- | --- | --- | --- | --- | --- | --- | --- | --- | --- | --- | --- |
| 1990 | male | 51.79506067 | 51.7475666 | 51.84255474 | 51.73579878 | 51.85432256 | 51.72205791 | 51.86806343 | 51.70472328 | 51.88539806 | 51.65694734 | 51.933174 |
| 1990 | female | 39.57361725 | 39.53694788 | 39.61028663 | 39.52786214 | 39.61937237 | 39.51725305 | 39.62998146 | 39.50386927 | 39.64336524 | 39.46698228 | 39.68025223 |
| 1991 | male | 51.87139289 | 51.82472372 | 51.91806206 | 51.81316029 | 51.92962549 | 51.79965808 | 51.9431277 | 51.78262452 | 51.96016125 | 51.73567838 | 52.00710739 |
| 1991 | female | 39.89261708 | 39.85638109 | 39.92885307 | 39.84740273 | 39.93783142 | 39.83691903 | 39.94831513 | 39.82369343 | 39.96154072 | 39.78724239 | 39.99799176 |
| 1992 | male | 52.2595938 | 52.21341889 | 52.30576871 | 52.20197793 | 52.31720968 | 52.18861872 | 52.33056889 | 52.17176556 | 52.34742205 | 52.12531661 | 52.39387099 |
| 1992 | female | 40.11784582 | 40.08191621 | 40.15377542 | 40.07301378 | 40.16267786 | 40.06261871 | 40.17307293 | 40.04950494 | 40.1861867 | 40.0133621 | 40.22232954 |
| 1993 | male | 53.27782528 | 53.23179787 | 53.32385269 | 53.22039345 | 53.3352571 | 53.20707692 | 53.34857364 | 53.1902776 | 53.36537296 | 53.14397703 | 53.41167353 |
| 1993 | female | 40.87730912 | 40.84142157 | 40.91319667 | 40.83252955 | 40.92208869 | 40.82214665 | 40.93247159 | 40.80904823 | 40.94557001 | 40.7729477 | 40.98167054 |
| 1994 | male | 54.49931181 | 54.45337091 | 54.54525271 | 54.44198792 | 54.5566357 | 54.42869642 | 54.56992721 | 54.41192867 | 54.58669495 | 54.36571512 | 54.6329085 |
| 1994 | female | 41.72598588 | 41.69010051 | 41.76187125 | 41.68120903 | 41.77076273 | 41.67082677 | 41.78114499 | 41.65772914 | 41.79424262 | 41.6216308 | 41.83034096 |
| 1995 | male | 55.38261828 | 55.33688522 | 55.42835133 | 55.32555374 | 55.43968282 | 55.31232236 | 55.45291419 | 55.29563048 | 55.46960608 | 55.24962601 | 55.51561055 |
| 1995 | female | 42.32445334 | 42.28867527 | 42.36023141 | 42.27981038 | 42.36909631 | 42.26945916 | 42.37944753 | 42.25640069 | 42.39250599 | 42.22041029 | 42.4284964 |
| 1996 | male | 55.65491976 | 55.60959608 | 55.70024344 | 55.59836603 | 55.71147349 | 55.5852531 | 55.72458642 | 55.56871063 | 55.74112889 | 55.52311797 | 55.78672155 |
| 1996 | female | 42.47415692 | 42.43868974 | 42.50962409 | 42.42990188 | 42.51841195 | 42.4196406 | 42.52867323 | 42.40669561 | 42.54161822 | 42.37101795 | 42.57729589 |
| 1997 | male | 56.14658933 | 56.10164232 | 56.19153634 | 56.0905056 | 56.20267306 | 56.07750164 | 56.21567702 | 56.06109665 | 56.23208201 | 56.01588289 | 56.27729576 |
| 1997 | female | 42.72446164 | 42.68926983 | 42.75965346 | 42.6805502 | 42.76837309 | 42.67036859 | 42.7785547 | 42.6575241 | 42.79139919 | 42.62212343 | 42.82679986 |
| 1998 | male | 56.74607594 | 56.70143752 | 56.79071436 | 56.69037726 | 56.80177462 | 56.67746259 | 56.8146893 | 56.66117023 | 56.83098166 | 56.61626689 | 56.87588499 |
| 1998 | female | 43.33664453 | 43.30157358 | 43.37171547 | 43.2928839 | 43.38040515 | 43.28273726 | 43.39055179 | 43.26993689 | 43.40335216 | 43.23465781 | 43.43863124 |
| 1999 | male | 56.83870116 | 56.79457094 | 56.88283137 | 56.7836366 | 56.89376572 | 56.77086896 | 56.90653336 | 56.75476208 | 56.92264023 | 56.71036996 | 56.96703235 |
| 1999 | female | 43.6470027 | 43.61218986 | 43.68181553 | 43.60356413 | 43.69044126 | 43.59349217 | 43.70051322 | 43.580786 | 43.71321939 | 43.54576657 | 43.74823883 |
| 2000 | male | 57.15867953 | 57.11494659 | 57.20241247 | 57.10411069 | 57.21324837 | 57.09145799 | 57.22590108 | 57.07549611 | 57.24186295 | 57.03150363 | 57.28585543 |
| 2000 | female | 43.75506253 | 43.72058091 | 43.78954414 | 43.71203725 | 43.7980878 | 43.70206111 | 43.80806394 | 43.68947584 | 43.82064921 | 43.65478958 | 43.85533547 |
| 2001 | male | 57.23241342 | 57.18917705 | 57.2756498 | 57.17846418 | 57.28636267 | 57.16595514 | 57.29887171 | 57.1501745 | 57.31465234 | 57.10668153 | 57.35814531 |
| 2001 | female | 43.30493199 | 43.27102536 | 43.33883862 | 43.26262416 | 43.34723982 | 43.25281438 | 43.3570496 | 43.24043896 | 43.36942502 | 43.2063311 | 43.40353287 |
| 2002 | male | 57.25809283 | 57.21539064 | 57.30079502 | 57.20481012 | 57.31137553 | 57.19245563 | 57.32373002 | 57.17686996 | 57.33931569 | 57.13391435 | 57.3822713 |
| 2002 | female | 42.96636047 | 42.93299706 | 42.99972388 | 42.92473046 | 43.00799048 | 42.91507784 | 43.0176431 | 42.90290069 | 43.02982025 | 42.86933928 | 43.06338166 |
| 2003 | male | 57.13396339 | 57.09185486 | 57.17607192 | 57.08142144 | 57.18650534 | 57.06923871 | 57.19868807 | 57.05386972 | 57.21405706 | 57.01151129 | 57.25641549 |
| 2003 | female | 42.58044035 | 42.54762408 | 42.61325662 | 42.53949304 | 42.62138765 | 42.52999872 | 42.63088198 | 42.51802127 | 42.64285943 | 42.48501024 | 42.67587046 |
| 2004 | male | 56.48610805 | 56.44483186 | 56.52738425 | 56.43460468 | 56.53761143 | 56.42266275 | 56.54955336 | 56.40759755 | 56.56461855 | 56.3660764 | 56.60613971 |
| 2004 | female | 41.93493171 | 41.90277215 | 41.96709127 | 41.89480383 | 41.97505959 | 41.88549951 | 41.98436391 | 41.87376175 | 41.99610167 | 41.84141133 | 42.02845209 |
| 2005 | male | 56.19887536 | 56.15828576 | 56.23946496 | 56.1482287 | 56.24952202 | 56.13648541 | 56.26126531 | 56.12167081 | 56.27607991 | 56.08084033 | 56.31691039 |
| 2005 | female | 41.50999053 | 41.47838325 | 41.5415978 | 41.47055177 | 41.54942928 | 41.46140723 | 41.55857382 | 41.44987104 | 41.57011001 | 41.41807619 | 41.60190487 |
| 2006 | male | 55.66598897 | 55.62618613 | 55.70579181 | 55.616324 | 55.71565394 | 55.60480835 | 55.7271696 | 55.5902809 | 55.74169704 | 55.55024184 | 55.7817361 |
| 2006 | female | 40.98325685 | 40.95226036 | 41.01425334 | 40.94458022 | 41.02193348 | 40.93561239 | 41.0309013 | 40.92429913 | 41.04221456 | 40.89311869 | 41.07339501 |
| 2007 | male | 55.72924374 | 55.69000399 | 55.7684835 | 55.68028138 | 55.77820611 | 55.66892863 | 55.78955885 | 55.6546067 | 55.80388078 | 55.61513407 | 55.84335341 |
| 2007 | female | 40.64209797 | 40.6116537 | 40.67254224 | 40.60411039 | 40.68008555 | 40.59530233 | 40.68889361 | 40.58419063 | 40.70000532 | 40.55356568 | 40.73063027 |
| 2008 | male | 55.84248804 | 55.80382769 | 55.8811484 | 55.79424864 | 55.89072745 | 55.78306352 | 55.90191256 | 55.76895306 | 55.91602302 | 55.73006327 | 55.95491281 |
| 2008 | female | 40.34150517 | 40.31158446 | 40.37142587 | 40.30417087 | 40.37883946 | 40.29551429 | 40.38749604 | 40.28459368 | 40.39841666 | 40.2544954 | 40.42851493 |
| 2009 | male | 55.73492863 | 55.69690818 | 55.77294908 | 55.68748769 | 55.78236958 | 55.67648771 | 55.79336956 | 55.66261081 | 55.80724646 | 55.62436472 | 55.84549255 |
| 2009 | female | 40.17495474 | 40.14550822 | 40.20440125 | 40.13821213 | 40.21169735 | 40.12969274 | 40.22021674 | 40.1189452 | 40.23096428 | 40.08932393 | 40.26058555 |
| 2010 | male | 56.01110462 | 55.97354471 | 56.04866453 | 55.96423833 | 56.05797092 | 55.95337159 | 56.06883766 | 55.93966278 | 56.08254647 | 55.90187996 | 56.12032928 |
| 2010 | female | 40.37723043 | 40.3481021 | 40.40635876 | 40.34088484 | 40.41357602 | 40.33245751 | 40.42200335 | 40.3218261 | 40.43263476 | 40.2925249 | 40.46193596 |
| 2011 | male | 56.14134229 | 56.1042976 | 56.17838699 | 56.09511887 | 56.18756572 | 56.08440119 | 56.1982834 | 56.07088043 | 56.21180416 | 56.03361588 | 56.24906871 |
| 2011 | female | 40.40316324 | 40.37442992 | 40.43189656 | 40.36731054 | 40.43901594 | 40.35899749 | 40.44732899 | 40.34851025 | 40.45781623 | 40.31960641 | 40.48672007 |
| 2012 | male | 56.20377476 | 56.16729243 | 56.24025708 | 56.15825304 | 56.24929647 | 56.14769807 | 56.25985144 | 56.13438256 | 56.27316695 | 56.09768373 | 56.30986578 |
| 2012 | female | 40.34156565 | 40.313262 | 40.36986929 | 40.30624908 | 40.37688221 | 40.29806034 | 40.38507095 | 40.28772993 | 40.39540136 | 40.25925831 | 40.42387298 |
| 2013 | male | 56.32697469 | 56.29100989 | 56.36293949 | 56.28209873 | 56.37185065 | 56.27169349 | 56.3822559 | 56.25856687 | 56.39538252 | 56.22238863 | 56.43156076 |
| 2013 | female | 40.15136489 | 40.12352366 | 40.17920611 | 40.11662532 | 40.18610446 | 40.10857036 | 40.19415941 | 40.09840873 | 40.20432105 | 40.07040227 | 40.2323275 |
| 2014 | male | 56.5338209 | 56.49831433 | 56.56932747 | 56.48951671 | 56.57812509 | 56.47924404 | 56.58839776 | 56.46628466 | 56.60135713 | 56.43056737 | 56.63707442 |
| 2014 | female | 40.14859888 | 40.12114938 | 40.17604839 | 40.11434809 | 40.18284968 | 40.10640647 | 40.1907913 | 40.0963878 | 40.20080996 | 40.06877539 | 40.22842238 |
| 2015 | male | 57.06038089 | 57.02522173 | 57.09554005 | 57.01651018 | 57.10425159 | 57.00633802 | 57.11442376 | 56.99350545 | 57.12725633 | 56.95813763 | 57.16262415 |
| 2015 | female | 40.33563861 | 40.30849109 | 40.36278613 | 40.30176462 | 40.36951259 | 40.29391037 | 40.37736684 | 40.28400193 | 40.38727529 | 40.2566933 | 40.41458392 |
| 2016 | male | 57.5229794 | 57.48819215 | 57.55776664 | 57.47957276 | 57.56638603 | 57.4695082 | 57.57645059 | 57.45681137 | 57.58914742 | 57.42181767 | 57.62414112 |
| 2016 | female | 40.49280569 | 40.46597522 | 40.51963616 | 40.45932731 | 40.52628407 | 40.45156479 | 40.53404659 | 40.44177206 | 40.54383932 | 40.41478236 | 40.57082902 |
| 2017 | male | 57.60417833 | 57.56989742 | 57.63845925 | 57.56140348 | 57.64695318 | 57.55148541 | 57.65687125 | 57.53897339 | 57.66938328 | 57.50448902 | 57.70386764 |
| 2017 | female | 40.31714525 | 40.29074638 | 40.34354412 | 40.28420542 | 40.35008508 | 40.27656776 | 40.35772274 | 40.26693257 | 40.36735793 | 40.24037703 | 40.39391347 |
| 2018 | male | 57.60181927 | 57.56805163 | 57.63558691 | 57.55968487 | 57.64395367 | 57.5499153 | 57.65372324 | 57.53759062 | 57.66604792 | 57.50362258 | 57.70001596 |
| 2018 | female | 40.30157726 | 40.27554301 | 40.3276115 | 40.26909239 | 40.33406213 | 40.26156023 | 40.34159429 | 40.25205812 | 40.3510964 | 40.22586937 | 40.37728515 |
| 2019 | male | 57.71950001 | 57.68620722 | 57.75279279 | 57.67795812 | 57.76104189 | 57.66832593 | 57.77067408 | 57.65617456 | 57.78282545 | 57.62268419 | 57.81631582 |
| 2019 | female | 40.25336252 | 40.22769531 | 40.27902973 | 40.22133563 | 40.28538941 | 40.21390965 | 40.29281538 | 40.2045415 | 40.30218353 | 40.17872196 | 40.32800307 |
| 2020 | male | 57.14236463 | 57.10966411 | 57.17506515 | 57.10156176 | 57.1831675 | 57.09210093 | 57.19262833 | 57.08016572 | 57.20456354 | 57.04727114 | 57.23745812 |
| 2020 | female | 39.78271938 | 39.75751676 | 39.807922 | 39.75127219 | 39.81416657 | 39.74398063 | 39.82145813 | 39.73478205 | 39.83065671 | 39.70942986 | 39.8560089 |
| 2021 | male | 57.00160298 | 56.96926657 | 57.03393939 | 56.96125443 | 57.04195153 | 56.95189894 | 57.05130702 | 56.94009663 | 57.06310933 | 56.90756831 | 57.09563765 |
| 2021 | female | 39.83507802 | 39.81009084 | 39.86006519 | 39.80389966 | 39.86625637 | 39.79667043 | 39.8734856 | 39.78755049 | 39.88260555 | 39.76241502 | 39.90774101 |
| 2022 | male | 57.03188723 | 56.45317193 | 57.61060252 | 56.30978105 | 57.7539934 | 56.14234859 | 57.92142587 | 55.93112609 | 58.13264837 | 55.34897628 | 58.71479817 |
| 2022 | female | 39.42296913 | 39.05203554 | 39.79390272 | 38.96012767 | 39.8858106 | 38.85281009 | 39.99312818 | 38.71742483 | 40.12851343 | 38.34428986 | 40.50164841 |
| 2023 | male | 57.30065451 | 56.57027131 | 58.03103771 | 56.38930099 | 58.21200803 | 56.17798834 | 58.42332068 | 55.91140931 | 58.68989971 | 55.17669149 | 59.42461753 |
| 2023 | female | 39.51460239 | 39.02271886 | 40.00648592 | 38.90084268 | 40.12836211 | 38.75853216 | 40.27067262 | 38.57900197 | 40.45020282 | 38.08419925 | 40.94500554 |
| 2024 | male | 57.57939835 | 56.71541681 | 58.44337989 | 56.50134423 | 58.65745246 | 56.25137925 | 58.90741745 | 55.93603881 | 59.22275789 | 55.06692978 | 60.09186692 |
| 2024 | female | 39.61564404 | 39.02138808 | 40.2099 | 38.87414662 | 40.35714146 | 38.70221797 | 40.52907011 | 38.48532336 | 40.74596472 | 37.88754066 | 41.34374742 |
| 2025 | male | 57.85968999 | 56.87253337 | 58.84684661 | 56.62794115 | 59.09143882 | 56.34233946 | 59.37704052 | 55.98204194 | 59.73733804 | 54.98902682 | 60.73035315 |
| 2025 | female | 39.72404588 | 39.03747221 | 40.41061956 | 38.86735677 | 40.58073499 | 38.66871899 | 40.77937278 | 38.41812978 | 41.02996198 | 37.72748148 | 41.72061028 |
| 2026 | male | 58.16949369 | 57.06489891 | 59.27408847 | 56.79120852 | 59.54777887 | 56.47162991 | 59.86735748 | 56.0684692 | 60.27051819 | 54.95731896 | 61.38166842 |
| 2026 | female | 39.85666857 | 39.08346971 | 40.62986743 | 38.89189077 | 40.82144637 | 38.6681908 | 41.04514634 | 38.38598469 | 41.32735245 | 37.60819712 | 42.10514002 |
| 2027 | male | 58.51611144 | 57.29683849 | 59.73538438 | 56.99473377 | 60.03748911 | 56.64197676 | 60.39024612 | 56.19696022 | 60.83526265 | 54.97045123 | 62.06177164 |
| 2027 | female | 40.01201525 | 39.15534114 | 40.86868935 | 38.94307916 | 41.08095133 | 38.69522834 | 41.32880215 | 38.38255501 | 41.64147549 | 37.52079678 | 42.50323371 |
| 2028 | male | 58.89127279 | 57.55979099 | 60.22275459 | 57.22988378 | 60.5526618 | 56.84466279 | 60.9378828 | 56.35869168 | 61.4238539 | 55.01930791 | 62.76323767 |
| 2028 | female | 40.18905383 | 39.25137498 | 41.12673268 | 39.01904209 | 41.35906556 | 38.74775519 | 41.63035247 | 38.40551632 | 41.97259134 | 37.46227261 | 42.91583504 |
| 2029 | male | 59.27644713 | 57.83495395 | 60.71794031 | 57.47778872 | 61.07510553 | 57.06073951 | 61.49215475 | 56.53461589 | 62.01827837 | 55.08456785 | 63.46832641 |
| 2029 | female | 40.37598987 | 39.35936681 | 41.39261294 | 39.10747355 | 41.64450619 | 38.8133467 | 41.93863305 | 38.44229437 | 42.30968538 | 37.41963793 | 43.33234182 |
| 2030 | male | 59.65889534 | 58.10914179 | 61.20864889 | 57.72515241 | 61.59263827 | 57.27678157 | 62.04100911 | 56.71114452 | 62.60664616 | 55.15219362 | 64.16559706 |
| 2030 | female | 40.57089521 | 39.47682218 | 41.66496824 | 39.2057388 | 41.93605162 | 38.88920431 | 42.2525861 | 38.48988389 | 42.65190653 | 37.38931784 | 43.75247258 |
| 2031 | male | 60.07397874 | 58.41590129 | 61.7320562 | 58.00507201 | 62.14288548 | 57.52536117 | 62.62259632 | 56.9201875 | 63.22776999 | 55.25226982 | 64.89568767 |
| 2031 | female | 40.79499179 | 39.62369444 | 41.96628913 | 39.33347685 | 42.25650672 | 38.99460003 | 42.59538354 | 38.56709387 | 43.0228897 | 37.38884521 | 44.20113836 |
| 2032 | male | 60.53555881 | 58.76756707 | 62.30355055 | 58.32950383 | 62.74161378 | 57.81799287 | 63.25312475 | 57.17270212 | 63.8984155 | 55.39421784 | 65.67689977 |
| 2032 | female | 41.04931457 | 39.80012058 | 42.29850855 | 39.49060218 | 42.60802695 | 39.12918849 | 42.96944064 | 38.67325122 | 43.42537791 | 37.41664362 | 44.68198551 |
| 2033 | male | 61.03016019 | 59.15135564 | 62.90896474 | 58.68583582 | 63.37448456 | 58.14226477 | 63.91805561 | 57.45652898 | 64.60379139 | 55.56657426 | 66.49374612 |
| 2033 | female | 41.32798681 | 40.00049085 | 42.65548277 | 39.67157123 | 42.9844024 | 39.2875034 | 43.36847022 | 38.80298707 | 43.85298655 | 37.4676128 | 45.18836083 |
| 2034 | male | 61.53471738 | 59.54488631 | 63.52454845 | 59.05185694 | 64.01757781 | 58.47616398 | 64.59327078 | 57.74990516 | 65.3195296 | 55.748265 | 67.32116976 |
| 2034 | female | 41.61593275 | 40.21012437 | 43.02174112 | 39.86180093 | 43.37006456 | 39.45507596 | 43.77678954 | 38.94197676 | 44.28988873 | 37.5278253 | 45.70404019 |
| 2035 | male | 62.03250837 | 59.93174108 | 64.13327566 | 59.41122456 | 64.65379219 | 58.8034358 | 65.26158094 | 58.03668691 | 66.02832984 | 55.92345216 | 68.14156459 |
| 2035 | female | 41.91082289 | 40.42658201 | 43.39506377 | 40.058825 | 43.76282079 | 39.62940812 | 44.19223766 | 39.08768222 | 44.73396356 | 37.59463279 | 46.22701299 |

Supplementary Table S15. All ages incidence, mortality and DALYs rate per 100 000 persons of MM in 1990 and 2021, along with EAPC per 100 000 persons from 1990 to 2021, categorized by global, SDI, and GBD regions.

| location | All ages incidence rate per 100,000 persons | | | | | | EAPC per 100,000 persons | All ages mortality rate per 100,000 persons | | | | | | EAPC per 100,000 persons | All ages DALYs rate per 100,000 persons | | | | | | EAPC per 100,000 persons |
| --- | --- | --- | --- | --- | --- | --- | --- | --- | --- | --- | --- | --- | --- | --- | --- | --- | --- | --- | --- | --- | --- |
|  | 1990-Male | 1990-Female | 2021-Male | 2021-Female | 1990-Both | 2021-Both |  | 1990-Male | 1990-Female | 2021-Male | 2021-Female | 1990-Both | 2021-Both |  | 1990-Male | 1990-Female | 2021-Male | 2021-Female | 1990-Both | 2021-Both |  |
| Global | 1.06(0.98,1.16) | 1.03(0.95,1.13) | 2.08(1.80,2.29) | 1.69(1.42,1.91) | 1.04(0.98,1.12) | 1.89(1.67,2.05) | 1.88(1.80,1.96) | 0.90(0.82,0.99) | 0.89(0.81,0.98) | 1.59(1.37,1.77) | 1.35(1.14,1.55) | 0.89(0.83,0.96) | 1.47(1.31,1.63) | 1.54(1.47,1.62) | 22.05(19.83,24.76) | 20.02(18.44,22.71) | 36.48(30.79,40.78) | 29.28(23.91,34.00) | 21.05(19.53,23.02) | 32.89(28.77,36.62) | 1.34(1.26,1.42) |
| High SDI | 3.91(3.80,4.01) | 3.67(3.39,3.84) | 7.08(6.55,7.50) | 5.41(4.64,5.86) | 3.79(3.59,3.92) | 6.24(5.61,6.63) | 1.59(1.46,1.73) | 3.25(3.14,3.32) | 3.15(2.89,3.29) | 5.16(4.74,5.46) | 4.25(3.63,4.62) | 3.20(3.02,3.29) | 4.70(4.18,5.01) | 1.13(1.04,1.22) | 73.99(72.19,75.56) | 64.81(60.76,67.15) | 101.75(94.96,107.02) | 76.90(68.92,82.33) | 69.33(66.62,71.06) | 89.30(81.97,94.50) | 0.69(0.60,0.79) |
| High-middle SDI | 1.17(1.10,1.29) | 1.19(1.10,1.31) | 2.88(2.36,3.27) | 2.46(2.05,2.79) | 1.18(1.11,1.27) | 2.67(2.32,2.96) | 2.66(2.59,2.73) | 0.94(0.87,1.04) | 0.97(0.90,1.07) | 2.05(1.70,2.33) | 1.85(1.52,2.11) | 0.95(0.90,1.03) | 1.95(1.70,2.16) | 2.31(2.23,2.38) | 24.45(22.52,27.10) | 23.33(21.72,26.29) | 48.47(38.59,55.55) | 40.99(32.74,47.21) | 23.88(22.54,26.06) | 44.73(38.66,50.03) | 1.98(1.90,2.05) |
| Middle SDI | 0.32(0.25,0.44) | 0.29(0.24,0.41) | 1.29(0.93,1.56) | 1.04(0.75,1.29) | 0.30(0.26,0.39) | 1.16(0.94,1.37) | 4.27(4.12,4.43) | 0.30(0.23,0.41) | 0.27(0.22,0.38) | 1.06(0.77,1.27) | 0.85(0.61,1.07) | 0.28(0.24,0.36) | 0.96(0.77,1.12) | 3.88(3.71,4.04) | 8.45(6.51,11.60) | 7.29(5.97,10.44) | 27.84(20.12,33.52) | 21.87(15.64,27.22) | 7.89(6.80,10.20) | 24.88(19.91,29.19) | 3.61(3.44,3.78) |
| Low-middle SDI | 0.30(0.18,0.45) | 0.25(0.17,0.37) | 0.73(0.58,1.08) | 0.64(0.48,0.92) | 0.28(0.20,0.37) | 0.69(0.59,0.96) | 2.96(2.82,3.10) | 0.29(0.18,0.44) | 0.25(0.17,0.37) | 0.69(0.55,1.00) | 0.59(0.45,0.86) | 0.27(0.20,0.36) | 0.64(0.55,0.90) | 2.79(2.66,2.91) | 8.19(5.04,12.17) | 6.77(4.56,10.22) | 18.25(14.71,26.89) | 15.40(11.61,22.18) | 7.49(5.40,9.94) | 16.83(14.29,23.41) | 2.63(2.50,2.76) |
| Low SDI | 0.27(0.12,0.43) | 0.23(0.12,0.38) | 0.35(0.21,0.52) | 0.33(0.21,0.44) | 0.25(0.14,0.35) | 0.34(0.23,0.46) | 0.94(0.67,1.21) | 0.27(0.12,0.42) | 0.22(0.12,0.37) | 0.33(0.21,0.50) | 0.32(0.20,0.43) | 0.25(0.14,0.35) | 0.33(0.22,0.44) | 0.84(0.58,1.09) | 7.49(3.33,11.84) | 6.18(3.22,10.51) | 9.24(5.67,14.16) | 8.63(5.41,11.51) | 6.84(3.77,9.67) | 8.93(5.92,12.18) | 0.76(0.50,1.01) |
| Andean Latin America | 0.74(0.50,0.97) | 0.48(0.33,0.71) | 1.79(1.35,2.43) | 1.42(1.07,1.90) | 0.61(0.45,0.78) | 1.60(1.25,2.09) | 3.47(3.28,3.66) | 0.72(0.49,0.94) | 0.46(0.31,0.68) | 1.52(1.14,2.07) | 1.15(0.89,1.50) | 0.59(0.43,0.74) | 1.34(1.05,1.73) | 3.00(2.82,3.17) | 19.23(13.14,25.07) | 11.85(8.10,17.66) | 38.59(28.69,52.27) | 28.58(21.88,37.21) | 15.52(11.36,19.50) | 33.59(26.12,43.91) | 2.77(2.59,2.95) |
| Australasia | 5.35(4.95,5.82) | 4.39(4.00,4.77) | 11.53(10.00,13.04) | 7.82(6.38,9.38) | 4.87(4.54,5.22) | 9.66(8.41,10.94) | 2.37(2.23,2.51) | 3.56(3.31,3.79) | 3.05(2.81,3.26) | 6.26(5.51,6.94) | 4.46(3.69,5.28) | 3.31(3.10,3.50) | 5.35(4.64,5.99) | 1.60(1.48,1.73) | 82.63(77.06,87.36) | 65.05(60.66,69.14) | 124.74(111.18,136.77) | 83.92(71.66,97.79) | 73.78(69.55,77.67) | 104.13(92.37,115.85) | 1.20(1.10,1.30) |
| Caribbean | 1.63(1.50,1.79) | 1.85(1.64,2.18) | 3.51(2.98,4.03) | 3.70(3.12,4.34) | 1.74(1.61,1.92) | 3.61(3.09,4.13) | 2.47(2.36,2.58) | 1.32(1.21,1.48) | 1.31(1.18,1.58) | 2.46(2.10,2.83) | 2.22(1.89,2.60) | 1.32(1.23,1.47) | 2.34(2.02,2.65) | 1.98(1.89,2.07) | 32.40(29.51,36.33) | 31.69(28.56,39.17) | 60.13(51.15,69.93) | 53.30(45.49,62.97) | 32.04(29.60,36.13) | 56.68(48.62,64.70) | 1.96(1.86,2.05) |
| Central Asia | 0.21(0.17,0.24) | 0.19(0.16,0.21) | 0.45(0.40,0.50) | 0.47(0.41,0.53) | 0.20(0.17,0.22) | 0.46(0.41,0.51) | 3.16(2.71,3.62) | 0.19(0.16,0.22) | 0.17(0.15,0.19) | 0.40(0.35,0.45) | 0.41(0.36,0.46) | 0.18(0.16,0.20) | 0.40(0.36,0.45) | 2.96(2.54,3.38) | 6.39(5.32,7.42) | 5.37(4.67,5.98) | 12.52(11.11,14.02) | 12.40(10.84,14.02) | 5.87(5.09,6.58) | 12.46(11.10,14.01) | 2.85(2.45,3.26) |
| Central Europe | 1.78(1.64,1.91) | 1.71(1.59,1.84) | 4.39(3.91,4.81) | 4.21(3.76,4.67) | 1.74(1.64,1.84) | 4.30(3.91,4.66) | 2.97(2.77,3.17) | 1.65(1.52,1.76) | 1.60(1.49,1.72) | 3.88(3.49,4.25) | 3.79(3.40,4.19) | 1.62(1.53,1.71) | 3.84(3.49,4.15) | 2.81(2.65,2.98) | 43.10(39.75,45.98) | 38.25(35.65,40.81) | 87.32(78.39,95.46) | 77.97(70.68,85.82) | 40.62(38.31,42.69) | 82.53(75.47,89.53) | 2.28(2.11,2.45) |
| Central Latin America | 0.59(0.57,0.61) | 0.53(0.51,0.55) | 1.75(1.52,1.99) | 1.53(1.35,1.72) | 0.56(0.54,0.58) | 1.64(1.46,1.84) | 3.44(3.32,3.55) | 0.55(0.53,0.57) | 0.48(0.46,0.50) | 1.44(1.26,1.64) | 1.20(1.06,1.34) | 0.51(0.50,0.53) | 1.32(1.18,1.48) | 3.06(2.94,3.17) | 15.53(14.98,15.99) | 13.12(12.62,13.59) | 38.93(33.88,44.46) | 31.44(27.72,35.44) | 14.31(13.87,14.69) | 35.09(31.28,39.39) | 2.88(2.76,2.99) |
| Central Sub-Saharan Africa | 0.17(0.10,0.25) | 0.12(0.06,0.18) | 0.18(0.10,0.31) | 0.17(0.06,0.28) | 0.15(0.10,0.20) | 0.17(0.10,0.25) | 0.56(0.17,0.95) | 0.17(0.10,0.24) | 0.12(0.06,0.18) | 0.17(0.09,0.30) | 0.16(0.06,0.27) | 0.15(0.10,0.20) | 0.17(0.09,0.24) | 0.43(0.07,0.79) | 4.85(2.76,7.04) | 3.52(1.70,5.20) | 5.08(2.75,9.01) | 4.63(1.65,7.69) | 4.18(2.74,5.71) | 4.86(2.64,7.08) | 0.52(0.14,0.89) |
| East Asia | 0.17(0.12,0.36) | 0.14(0.09,0.34) | 1.50(0.88,2.09) | 0.96(0.43,1.35) | 0.16(0.11,0.30) | 1.23(0.81,1.60) | 6.39(5.78,7.00) | 0.15(0.10,0.33) | 0.14(0.09,0.32) | 1.08(0.64,1.52) | 0.76(0.34,1.07) | 0.14(0.10,0.28) | 0.93(0.61,1.20) | 5.56(4.90,6.22) | 4.56(3.14,9.73) | 3.94(2.49,9.12) | 28.39(16.45,39.49) | 19.53(8.57,27.34) | 4.26(3.04,8.08) | 24.06(15.53,31.51) | 5.21(4.57,5.85) |
| Eastern Europe | 1.22(1.14,1.33) | 1.37(1.29,1.44) | 2.73(2.41,3.02) | 3.20(2.87,3.53) | 1.30(1.23,1.38) | 2.98(2.76,3.24) | 2.80(2.51,3.08) | 1.00(0.94,1.08) | 1.16(1.10,1.21) | 2.00(1.78,2.22) | 2.46(2.21,2.73) | 1.09(1.03,1.14) | 2.25(2.08,2.44) | 2.45(2.22,2.68) | 29.83(27.70,32.15) | 31.12(29.48,32.59) | 54.00(47.74,59.92) | 60.44(54.06,67.36) | 30.52(28.86,32.16) | 57.44(52.74,62.29) | 2.04(1.83,2.26) |
| Eastern Sub-Saharan Africa | 0.37(0.16,0.58) | 0.31(0.16,0.47) | 0.49(0.28,0.74) | 0.48(0.26,0.64) | 0.34(0.18,0.48) | 0.48(0.29,0.66) | 1.13(0.87,1.39) | 0.37(0.16,0.57) | 0.31(0.16,0.47) | 0.47(0.26,0.71) | 0.46(0.25,0.62) | 0.34(0.18,0.48) | 0.46(0.28,0.63) | 1.01(0.76,1.26) | 10.18(4.44,16.01) | 8.44(4.30,13.15) | 13.43(7.46,20.81) | 12.64(6.83,16.92) | 9.30(5.00,13.42) | 13.03(7.79,18.13) | 1.05(0.80,1.29) |
| High-income Asia Pacific | 2.34(2.17,2.49) | 2.24(2.00,2.45) | 5.60(4.93,6.31) | 4.92(3.81,5.88) | 2.29(2.12,2.42) | 5.25(4.40,5.88) | 2.80(2.59,3.02) | 1.78(1.69,1.87) | 1.71(1.55,1.87) | 3.98(3.53,4.39) | 3.55(2.72,4.11) | 1.75(1.64,1.84) | 3.76(3.14,4.19) | 2.37(2.21,2.53) | 42.14(39.93,44.50) | 37.75(34.55,40.88) | 71.27(62.78,79.51) | 56.52(45.39,65.12) | 39.92(37.82,41.90) | 63.77(54.68,70.26) | 1.39(1.20,1.58) |
| High-income North America | 4.58(4.41,4.72) | 3.86(3.55,4.03) | 6.75(6.28,7.06) | 4.58(4.06,4.89) | 4.21(3.98,4.35) | 5.65(5.14,5.95) | 0.81(0.73,0.88) | 4.37(4.21,4.49) | 3.96(3.60,4.16) | 5.98(5.55,6.25) | 4.52(3.95,4.84) | 4.16(3.91,4.30) | 5.23(4.74,5.53) | 0.52(0.43,0.62) | 98.50(95.56,100.94) | 81.50(76.33,84.63) | 118.48(112.54,123.33) | 84.18(76.40,89.07) | 89.81(86.22,92.22) | 101.04(94.28,105.57) | 0.18(0.10,0.26) |
| North Africa and Middle East | 0.45(0.28,0.66) | 0.36(0.24,0.60) | 1.03(0.70,1.39) | 0.84(0.53,1.19) | 0.40(0.28,0.55) | 0.94(0.70,1.28) | 2.84(2.69,2.99) | 0.42(0.26,0.63) | 0.33(0.22,0.55) | 0.84(0.58,1.14) | 0.67(0.43,0.92) | 0.38(0.26,0.52) | 0.76(0.57,1.04) | 2.31(2.18,2.43) | 11.74(7.16,17.14) | 9.23(6.01,15.86) | 22.18(15.27,30.02) | 17.74(11.26,24.58) | 10.51(7.25,14.38) | 20.05(15.01,27.58) | 2.15(2.01,2.29) |
| Oceania | 0.16(0.07,0.28) | 0.12(0.07,0.20) | 0.18(0.09,0.30) | 0.20(0.11,0.28) | 0.14(0.09,0.20) | 0.19(0.11,0.27) | 1.00(0.91,1.10) | 0.15(0.06,0.26) | 0.11(0.07,0.18) | 0.17(0.08,0.28) | 0.18(0.09,0.26) | 0.13(0.08,0.19) | 0.17(0.10,0.25) | 0.96(0.86,1.05) | 4.18(1.84,7.54) | 3.36(2.06,5.63) | 4.60(2.20,8.04) | 5.44(2.85,8.18) | 3.79(2.29,5.64) | 5.01(2.76,7.28) | 0.96(0.86,1.06) |
| South Asia | 0.37(0.19,0.50) | 0.28(0.15,0.41) | 0.97(0.65,1.34) | 0.75(0.49,1.13) | 0.32(0.21,0.41) | 0.86(0.68,1.17) | 3.08(2.85,3.31) | 0.36(0.19,0.49) | 0.27(0.15,0.40) | 0.90(0.61,1.25) | 0.70(0.46,1.06) | 0.32(0.20,0.40) | 0.80(0.63,1.08) | 2.90(2.69,3.11) | 10.18(5.18,13.99) | 7.47(4.14,11.22) | 23.64(15.98,32.66) | 17.79(11.67,26.39) | 8.88(5.58,11.29) | 20.77(16.39,27.72) | 2.64(2.43,2.86) |
| Southeast Asia | 0.16(0.12,0.29) | 0.16(0.12,0.29) | 0.51(0.36,0.87) | 0.50(0.34,0.83) | 0.16(0.13,0.26) | 0.50(0.39,0.81) | 3.62(3.52,3.71) | 0.15(0.11,0.27) | 0.15(0.12,0.27) | 0.43(0.30,0.74) | 0.42(0.29,0.71) | 0.15(0.12,0.25) | 0.43(0.33,0.69) | 3.31(3.22,3.39) | 4.27(3.17,7.82) | 4.28(3.25,7.61) | 11.95(8.31,20.39) | 11.13(7.80,18.63) | 4.28(3.40,6.91) | 11.54(9.01,18.39) | 3.17(3.09,3.24) |
| Southern Latin America | 2.17(1.99,2.38) | 1.99(1.79,2.22) | 3.35(3.07,3.65) | 2.67(2.36,2.96) | 2.08(1.93,2.25) | 3.00(2.77,3.24) | 1.28(1.13,1.43) | 2.00(1.83,2.20) | 1.87(1.68,2.09) | 2.69(2.49,2.89) | 2.27(2.00,2.47) | 1.93(1.80,2.09) | 2.47(2.28,2.65) | 0.87(0.72,1.02) | 49.98(45.87,55.16) | 43.52(39.15,48.48) | 63.84(58.82,68.65) | 48.20(43.73,52.16) | 46.68(43.67,50.32) | 55.84(52.26,59.51) | 0.65(0.51,0.78) |
| Southern Sub-Saharan Africa | 0.77(0.45,1.11) | 0.77(0.51,1.05) | 1.63(0.97,2.05) | 1.73(1.03,2.28) | 0.77(0.53,0.99) | 1.68(1.11,2.04) | 2.63(2.54,2.72) | 0.73(0.42,1.06) | 0.74(0.49,1.01) | 1.47(0.88,1.84) | 1.60(0.95,2.09) | 0.74(0.50,0.96) | 1.54(1.02,1.86) | 2.46(2.35,2.58) | 21.32(12.53,30.38) | 20.04(12.97,27.10) | 43.08(25.82,54.97) | 43.38(26.16,56.92) | 20.66(14.33,26.02) | 43.23(28.82,53.03) | 2.50(2.40,2.61) |
| Tropical Latin America | 0.79(0.75,0.83) | 0.78(0.74,0.82) | 2.43(2.28,2.58) | 2.33(2.11,2.51) | 0.79(0.75,0.82) | 2.38(2.22,2.50) | 3.60(3.46,3.74) | 0.73(0.70,0.76) | 0.70(0.67,0.74) | 2.09(1.96,2.22) | 1.94(1.76,2.08) | 0.72(0.69,0.74) | 2.02(1.87,2.12) | 3.40(3.28,3.52) | 20.81(19.78,21.69) | 19.15(18.23,20.02) | 53.18(49.95,56.08) | 46.72(43.00,49.58) | 19.97(19.27,20.67) | 49.88(47.17,51.99) | 2.95(2.82,3.08) |
| Western Europe | 5.42(5.19,5.63) | 5.36(4.93,5.64) | 10.69(9.76,11.49) | 8.19(6.93,8.93) | 5.39(5.08,5.60) | 9.42(8.44,10.07) | 1.85(1.66,2.04) | 3.97(3.82,4.09) | 4.04(3.69,4.24) | 6.73(6.10,7.18) | 5.58(4.73,6.12) | 4.00(3.77,4.14) | 6.14(5.43,6.60) | 1.40(1.29,1.50) | 89.10(86.34,92.09) | 80.73(75.44,84.38) | 128.35(118.68,136.66) | 97.24(84.64,105.06) | 84.81(80.92,87.60) | 112.52(102.28,119.70) | 0.89(0.77,1.01) |
| Western Sub-Saharan Africa | 0.09(0.05,0.13) | 0.13(0.05,0.20) | 0.12(0.06,0.17) | 0.24(0.07,0.39) | 0.11(0.06,0.16) | 0.18(0.07,0.27) | 1.69(1.48,1.90) | 0.09(0.05,0.13) | 0.13(0.05,0.21) | 0.12(0.06,0.17) | 0.23(0.07,0.37) | 0.11(0.06,0.16) | 0.18(0.07,0.25) | 1.51(1.32,1.70) | 2.54(1.39,3.54) | 3.27(1.27,5.13) | 3.28(1.56,4.59) | 6.02(1.70,10.01) | 2.91(1.50,4.05) | 4.69(1.93,6.92) | 1.65(1.45,1.85) |
